# Pilot implementation of intermittent preventive treatment with dihydroartemisinin–piperaquine to prevent adverse birth outcomes in Papua, Indonesia: a mixed-method evaluation

**DOI:** 10.1016/j.lanprc.2025.100011

**Published:** 2025-07

**Authors:** Firdaus Hafidz, Freis Candrawati, Jenna Hoyt, Enny Kenangalem, James Dodd, Maia Lesosky, Ida Safitri Laksanawati, Reynold Ubra, Minerva Simatupang, Feiko O ter Kuile, Eve Worrall, Jeanne Rini Poespoprodjo, Jenny Hill

**Affiliations:** a Department of Clinical Sciences, Liverpool School of Tropical Medicine, Liverpool, UK; b Department of Health Policy and Management, Faculty of Medicine, Public Health and Nursing, Universitas Gadjah Mada, Yogyakarta, Indonesia; c Timika Research Facility, Papuan Health and Community Development Foundation, Timika, Indonesia; d National Heart and Lung Institute, Imperial College London, London, UK; e Department of Child Health, Faculty of Medicine, Public Health and Nursing, Universitas Gadjah Mada and Dr Sardjito Hospital, Yogyakarta, Indonesia; f Centre for Child Health-PRO, Faculty of Medicine, Public Health and Nursing, Universitas Gadjah Mada, Yogyakarta, Indonesia; g Mimika District Health Office, Timika, Indonesia; h Indonesian Ministry of Health, Jakarta, Indonesia

## Abstract

**Background:**

A previous trial showed that intermittent preventive treatment with dihydroartemisinin–piperaquine (IPTp-DP) was more effective than the current policy of single screening and treatment in preventing malaria during pregnancy in Papua, Indonesia. The STOPMiP-2 study evaluated the Ministry of Health pilot implementation of IPTp-DP through routine antenatal care in Papua.

**Methods:**

A mixed-method evaluation was conducted in ten primary health-care facilities in the Mimika district in Papua, Indonesia from June 8, 2022, to Dec 27, 2023. Pregnant women aged 15–49 years who were HIV negative (when status known), in their second or third trimester of pregnancy, and provided written informed consent were eligible. IPTp-DP delivery effectiveness (3-day doses of three tablets [ie, nine tablets] with the first dose by directly observed therapy during antenatal care) and adherence (completion of all nine tablets, ascertained by pill count) were coprimary outcomes. Analyses were done in the modified intention-to-treat (mITT) population (defined for delivery effectiveness as all women who completed exit interviews, and for treatment adherence as all women who had a home visit). The mITT population excluded women with fever or malaria infection, those with a positive malaria test, or those who received IPTp-DP outside the designated timeframe (ie, less than 4 weeks between courses). We explored predictors of delivery effectiveness and adherence using multivariable logistic regression, and used qualitative data to provide explanatory insights. We used routine health information to assess monthly coverage by facility. This study was registered at ClinicalTrials.gov (NCT05294406) and is now complete.

**Findings:**

From June 8, 2022, to Dec 27, 2023, we enrolled 1420 pregnant women in exit interviews, of whom 1366 had data available and were eligible for the effectiveness analysis. 490 women were visited at home, of whom 484 had data available and were eligible for the adherence analysis. 556 (41%) of 1366 women had effective delivery of IPTp-DP, and among those with available data, 437 (90%) of 484 had full adherence. Predictors of full effective delivery versus partial or non-effective delivery were older maternal age (≥35 years *vs* 20–34 years: adjusted odds ratio 1·26 [95% CI 1·04–1·51], p=0·017), having a lower level of education (no education or primary education *vs* diploma or university: 2·01 [1·08–3·75], p=0·028), being in the second trimester (*vs* third trimester; 3·13 [2·11–4·63], p<0·0001), had previous IPTp-DP (*vs* no previous IPTp-DP: 4·30 [3·07–6·01], p<0·0001), and not having health insurance (*vs* health insurance: 1·33 [1·09–1·63], p=0·0044). No difference was seen by younger age (age 15–19 years), middle or high school education, ethnicity, marital status, previous malaria test within past 28 days, and location. Predictors of adherence were being married (*vs* being single, divorced, or widowed: 3·50 [1·55–7·89], p=0·0028), having attended four or more antenatal care visits (*vs* attending three or fewer: 1·95 [1·22–3·13], p=0·0054), and full delivery effectiveness (*vs* partial delivery effectiveness: 3·18 [1·82–5·54], p<0·0001). No difference was seen by gestational age for adherence. Between Dec 1, 2022, and Nov 22, 2023, across facilities, 1630 (43%) of 3815 women attending their first antenatal care visit received one course of IPTp-DP, 949 (25%) received two courses, and 880 (23%) received three or more courses.

**Interpretation:**

Among those who received IPTp-DP, adherence to the 3-day IPTp-DP regimen was high; however, the sample size for adherence was smaller than anticipated owing to lower-than-expected full delivery effectiveness. Future studies should investigate strategies to improve treatment delivery in this setting.

**Funding:**

UK Medical Research Council.

## Introduction

Malaria during pregnancy remains a prevalent public health concern. In southeast Asia in 2020, approximately 52·9 million people were pregnant and at risk of malaria, equating to 43% of the global total of people who are pregnant and at risk of malaria.^1^ Malaria in pregnancy is a threat to health, and can lead to severe maternal anaemia; stillbirth; preterm birth; poor fetal growth; low birthweight; and increased perinatal, neonatal, and infant mortality.^2^ In this region, malaria during pregnancy has been linked to an increased risk of stillbirth and neonatal death, primarily due to small-for-gestational-age status and preterm birth.^3^

**Research in context**

**Evidence before this study**

Malaria in pregnancy causes serious health risks including maternal anaemia, low birthweight, and preterm delivery. Monthly intermittent preventive treatment with dihydroartemisinin–piperaquine (IPTp-DP), a multiday regimen, showed superior efficacy compared to standard of care with single screening and treatment in a clinical trial in Papua, Indonesia. Nested acceptability, feasibility, and economic analyses showed IPTp-DP was acceptable and cost effective in this setting with some concerns for scalability. However, there is limited evidence on its delivery effectiveness, adherence, and coverage in routine health-care settings. We conducted a search of two databases during writing of the report: the Malaria in Pregnancy Library from its inception up until May 27, 2024 (date of last update), and PubMed from its inception up until Oct 17, 2024 using the following terms: “intermittent” AND “prevent∗” AND “treat∗” AND “dihydroartemisinin-piperaquine” AND “pregnan∗” AND (“adherence” OR “effective∗” OR “delivery” OR “efficacy”). The results showed that up until Dec 31, 2021, before we started our study, five studies had focused on IPTp-DP efficacy, safety, and cost-effectiveness, yet none specifically examined its real-word implementation in routine health-care settings. During analysis, we found four new studies. Among these four studies, we identified only one real-world implementation trial in Kenya, which showed that targeted information transfer (intervention) to health providers and pregnant women significantly improved adherence rates in pregnant women by 16% compared with the control group. Studies on IPTp with sulphadoxine–pyrimethamine in sub-Saharan Africa have shown that uptake and adherence are influenced by factors such as patient autonomy, household decision makers, and motivation and training of health-care providers.

**Added value of this study**

To our knowledge, this is the first study to assess the delivery effectiveness and adherence to IPTp-DP pilot implementation through routine antenatal care in a high-burden setting. Previous research focused on the clinical safety and efficacy of IPTp-DP, whereas the feasibility of implementation in real-world, resource-constrained settings remains largely unexplored. We anticipated challenges related to delivery and adherence outside of trial settings. Despite challenges in IPTp-DP delivery, our findings reveal that adherence rates among pregnant women were remarkably high. A separate in-depth thematic analysis of the qualitative data, as well as cost and cost-effectiveness analyses, will be reported elsewhere.

**Implications of all the available evidence**

Our findings indicate that IPTp-DP is a viable addition to single screening and treatment (ie, the current national recommendation in Indonesia), supporting policy adoption and expansion of IPTp-DP for prevention of malaria in the second and third trimesters of pregnancy in Indonesia. This study shows that implementing a complex, multiday IPTp-DP regimen in routine antenatal care is feasible with tailored adaptations for delivery to suit local conditions, showing high adherence. Efforts to scale- up coverage in other high-incidence areas of Indonesia will require strengthening health-care worker capacity for effective delivery of IPTp-DP, consistent drug availability, and adaptations to routine health management information systems.

The health implications of malaria in pregnancy underscore the potential effect of effective malaria control in pregnancy on improving pregnancy outcomes in southeast Asia. Traditionally, malaria prevention in the Asia–Pacific region has relied on long-lasting insecticide-treated nets and passive case detection for managing febrile cases.^4^ In Indonesia, the current national malaria policy recommends single screening and treatment (SST) at the first antenatal care visit.^5^ WHO recommends intermittent preventive treatment in pregnancy (IPTp) using sulphadoxine–pyrimethamine (IPTp-SP) at every scheduled antenatal care visit during the second and third trimesters in areas with moderate-to-high malaria transmission.^6^ However, this policy has not been adopted in Indonesia due to the widespread drug resistance to sulphadoxine–pyrimethamine.^7^

A previous trial in Papua, Indonesia, showed that IPTp with dihydroartemisinin–piperaquine (IPTp-DP) is safe, well tolerated, and efficacious in preventing malaria infection and anaemia at delivery.^8^ Nested studies showed that IPTp-DP was also cost-effective^9^ and acceptable to pregnant women (ie, women were happy to take anti-malarial drugs presumptively to protect themselves and their babies), although health-care providers expressed unsubstantiated concerns that it could cause harm to the mother or baby, or both.^10^ A key difference between IPTp-SP and IPTp-DP is in the regimen. Sulphadoxine–pyrimethamine is administered as a single dose under directly observed therapy (DOT) at health facilities, whereas dihydroartemisinin–piperaquine is taken over 3 consecutive days, with only the first dose observed and the remainder self-administered at home.^11^

The Indonesian Ministry of Health (MOH) initiated a pilot programme of IPTp-DP as a new malaria prevention intervention strategy for pregnant women in the second and third trimester in Papua, Indonesia. IPTp-DP is delivered in addition to SST, which occurs in the first trimester. Implementing IPTp-DP would represent a substantial task-shifting to allow midwives to prescribe antimalarials, which is currently restricted to clinicians. In this study, we aimed to evaluate the pilot programme, specifically the feasibility of routine antenatal care services to deliver IPTp-DP effectively and pregnant women's adherence to the multiday regimen. Results from nested acceptability, affect on maternal and pregnancy outcomes, and cost-effectiveness studies are presented elsewhere.

## Methods

### Study design and participants

This was a mixed-method evaluation study of the IPTp-DP pilot programme in Papua, Indonesia undertaken between June 8, 2022, and Dec 27, 2023. Qualitative data collection was undertaken at two timepoints, at study midline 4 months after implementation began, from June 8 to July 27, 2022, and again at study endline, from May 2 to Dec 27, 2023. Cross-sectional surveys involving exit interviews at antenatal care clinics and home visits were conducted from May 6 to Oct 25, 2023. Routine Health Management Information System (HMIS) data were collected from Feb 7, 2022, to Nov 22, 2023. The study protocol is provided in appendix 2 (pp 57–89), and the study is registered with ClinicalTrials.gov (NCT05294406). We have adhered to the CRISP reporting guidelines^12^ (appendix 2 pp 43–49) for primary care research and the ASSESS guidance^13^ (appendix 2 pp 50–56) for mixed-methods analysis in implementation research.

The IPTp-DP pilot programme was launched on Feb 7, 2022, in ten primary health-care facilities (*puskesmas*) in Mimika district, Papua, Indonesia (appendix 2 p 1). Although the district has a network of 26 primary health-care facilities and 193 auxiliary health centres, the pilot was restricted to public facilities including their health posts (*posyandu*) and auxiliary health centres (*pustu*) that were accessible by road all year round. Secondary level health-care services are primarily delivered by the Mimika District Public Hospital, supervised by the District Health Office (DHO). Community-based health initiatives are represented by integrated health posts, which are managed by local communities with technical support from *puskesmas*. These centres focus on maternal and child health, immunisation, and nutrition.

Per protocol, health facilities were eligible for the study if they had an operational and accessible antenatal clinic, and if they had midwives or nurses who had been trained as part of the pilot implementation to prescribe IPTp-DP. Women aged 15–49 years who were in the second or third trimester of pregnancy and provided written informed consent were eligible for inclusion. Eligibility of inclusion was also assessed based on HIV-negative status (if status was known). However, women with unknown HIV status were also included in the study. The eligibility assessment took place during the consenting process for exit interviews, before recruitment into the study. Women speaking languages other than Indonesian, those who had a severe illness that could interfere with participation, or those who moved outside the pilot implementation area were excluded. Eligibility criteria for health-care providers, community leaders, husbands of pregnant women, and community health workers who participated in in-depth interviews are provided in the protocol (pp 65–67).

Mimika is a highly malaria-endemic region, where both *Plasmodium falciparum* and *Plasmodium vivax* are widely prevalent. In 2023, the annual parasite incidence was 525 per 1000 population and this region contributed 35% of all malaria cases in Indonesia.^14^ Dihydroartemisinin–piperaquine has been the first-line treatment for uncomplicated malaria in the general population since 2006, and in pregnant women in all trimesters since 2019.^15^ The pilot implementation of IPTp-DP was expected to adhere to the delivery algorithm as outlined in the decree by the DHO (appendix 2 p 34).

SST was to be given to women attending antenatal care visits in their first trimester. IPTp-DP involves the administration of a fixed-dose (40 mg dihydroartemisinin and 320 mg piperaquine) treatment regimen of three tablets per day for 3 consecutive days (totalling nine tablets) to pregnant women attending routine, monthly antenatal care visits in their second and third trimester. The first dose was to be administered by an antenatal care nurse under DOT, and the remaining doses were to be given to women to take at home with health-care providers or community health workers making follow-up contacts at home or phone calls to promote adherence. Women were expected to receive at least three courses of IPTp-DP over the course of their pregnancy.

Health-care providers in the pilot facilities were trained by the study team before pilot implementation. Continuous quality improvement (CQI) practices that had been previously used by the MOH were refocused to support the introduction of IPTp, whereas pharmacovigilance activities were strengthened through refresher training and using standardised national reporting procedures. Health promotion materials (eg, job aids, leaflets, and informational videos) were codesigned by the MOH with support from the study team and distributed by the DHO. Intervention fidelity and any adaptations or modifications reported during CQI activities were tracked and are reported using the Template for Intervention Description and Replication checklist^16^ (appendix 2 pp 35–42).

The pilot programme was rolled out in the ten facilities through a series of codesign workshops, meetings, and training workshops in January, 2022, led by the Mimika DHO with support from the Provincial Health Office and the study team. A technical training webinar for all facility staff and district managers was followed by CQI training to establish an IPTp-DP team in each facility and identify key performance indicators (aligning with phase one of the SQALE initiative, which focuses on enhancing maternal and child health by reinforcing community health systems in Kenya through structured quality improvement processes process).^17^ Four further CQI sessions were held to review progress. These sessions were planned to take place four times a year, but scheduling was adjusted based on the needs of the DHO and took place in May and December, 2022, and in March and November, 2023. Stakeholder engagement, including meetings with community health workers, was led by district and subdistrict staff. Communication training was provided for facility staff to support mobilisation in the community. The timeline of pilot and study activities is provided in the appendix 2 (p 2).

The pilot was halted temporarily, varying from facility to facility based on available stocks between June and September, 2022, due to a nationwide stockout of dihydroartemisinin–piperaquine owing to cessation of manufacturing during the COVID-19 pandemic, when MOH prioritised the limited supplies for case management. Dihydroartemisinin–piperaquine for IPTp was supplied by the study team from Oct 7, 2022, to April 28, 2023, until MOH stocks stabilised.

During the pilot, routine HMIS records were modified to collect IPTp-DP coverage data and data on side-effects. Stickers in maternal health books served to remind providers to record women’s IPTp-DP administration.

The study received ethical approval from the Liverpool School of Tropical Medicine (number 21-054) and the Universitas Gadjah Mada Research Ethics Committees (number 1198). Two protocol amendments were made, V2.2 approved on June 7, 2022, and V2.3 approved on Sept 8, 2023. Full details of protocol amendments are provided in the appendix 2 (pp 57–58). All study participants provided written informed consent. Consent was not required for routine HMIS data because no individual-level data were collected.

### Procedures

19 trained data collectors conducted interviews, comprising ten research midwives and nine locally recruited fieldworkers trained by the study team (appendix 2 p 3). Pregnant women were interviewed after they completed their antenatal care visit at one of the ten pilot facilities, including their affiliated lower-level facilities, using a structured questionnaire. Each morning, based on the estimated number of antenatal care attendees, the study team randomly selected which women would be approached for participation by lottery. Women were screened by antenatal care midwives and then selected from a predetermined randomisation list. Electronic data from exit interviews and home visits were collected using questionnaires adapted from a previous study,^5^ programmed in the Open Data Kit platform (version 2023). Daily data validity checks were carried out by the site investigator, and any discrepancies were resolved to ensure data integrity. Interviews to collect electronic data were conducted in a private area. Questions covered socioeconomic information; obstetric history; antenatal care services, drugs, or tests received, including IPTp-DP; knowledge, acceptability, or any experience of taking IPTp-DP; and antenatal care visit costs. Permission was obtained to review the maternal health cards and observe any medication provided. Pregnant women who received IPTp-DP and consented to a home visit were visited at home 3–4 days after the exit interview to assess adherence via in-person interview and pill count.

We conducted in-depth interviews with policy makers, health managers, health-care providers, pregnant women, husbands, community health workers, and community leaders. Interviews were used to provide explanatory insights for key study outcomes. In-depth interviews with health workers, health managers, and policy makers explored health system perspectives of the drivers of successful integration of IPTp-DP in antenatal care. In-depth interviews with pregnant women aimed to assess acceptability of IPTp-DP to refine strategies for improving uptake and adherence. Data were collected at two timepoints, with themes emerging from study midline collection informing the selection of additional qualitative participants (ie, husbands, community health workers, and community leaders) and themes at study endline. Additional data collection at endline included in-depth interviews with husbands of pregnant women and community health workers and focus group discussions with community leaders. These additional interviews aimed to capture perspectives on IPT-p-DP implementation from community leaders and adherence support from partners and community health workers. All interviews were conducted in person (unless there were availability constraints) in Indonesian, recorded digitally, and transcribed and translated into English. The approximate duration of interviews with health managers was 40–50 min, 60 min for health workers, 45–60 min for pregnant women, 45 min for community health workers, 130 min for community leaders, and 30 min for husbands.

Routine HMIS data on IPTp-DP coverage (ie, the ratio of the number of IPTp-DP courses administered to the number of first antenatal care visits) and side-effects were collected retrospectively from IPTp registers and health centre monthly reports by the study team. Data included health facility name; dates of IPTp-DP administration; number of first or other antenatal care visits; number of IPTp-DP courses administered; the total number of IPTp-DP courses administered (ranging from one to six courses), and side-effects experienced. Data were entered into Google Sheets and displayed on a Looker Studio dashboard on Google Cloud for real-time monitoring.

During the four CQI workshops held at district level for all pilot facilities, facility representatives reported progress on key indicators, including inputs such as IPTp-DP readiness (eg, regulations and supplies) and outputs (eg, proportion of integrated health posts providing IPTp-DP, number of days of IPTp-DP services, and overall IPTp-DP coverage). Summary reports were prepared by the study team to provide context for the evaluation findings.

### Outcomes

The coprimary outcomes were delivery effectiveness and adherence. These coprimary outcomes are a deviation from the protocol because adherence could only be assessed among those with full or partial delivery effectiveness and was revised in the statistical analysis plan before analysis (appendix 2 pp 91–98). Delivery effectiveness was defined as the proportion of women attending antenatal care who were administered with an appropriate dose of IPTp-DP (any course) according to the MOH guidelines. Adherence was defined as the proportion of pregnant women receiving a correct dose who completed all three IPTp-DP doses, verified by pill counts at home visits by the study team. When no pills remained, women were asked additional questions during the home visit interview to verify whether the course had been completed as instructed.

We categorised IPTp-DP delivery effectiveness into three levels to capture the range of delivery and adherence practices observed: (1) full delivery effectiveness (three tablets given by DOT and six tablets for subsequent doses), (2) partial delivery effectiveness (nine tablets given without DOT), and (3) non-effective delivery (any deviation from the previous two definitions). We categorised adherence into three levels: (1) full adherence (women who reported they had completed the remaining six or nine tablets at home, depending on whether they received full or partial delivery effectiveness), (2) partial adherence (women who reported partial completion of the remaining six or nine tablets), and (3) non-adherence (women who reported they had not taken any tablets at home). This categorisation was developed during analysis.

Qualitative outcomes explored acceptability, feasibility, and fidelity of IPTp-DP from the perspectives of health providers, pregnant women, and community members. Fidelity was also assessed through CQI documentation.

### Statistical analysis

For exit interviews and home visits, a sample size of 1080 pregnant women would allow a point estimate of 60% of women attaining the adherence outcome^18^ to be measured with a 95% CI and an SD of 6 assuming an intra-cluster correlation coefficient (ICC) of 0·03.^19^ We aimed to enrol a total of 1440 women for the delivery effectiveness outcome. Allowing for an expected delivery effectiveness of 75%, we estimated that 1080 women would be available for the analysis of adherence. We aimed to complete 108 interviews per facility on average, with actual numbers adjusted according to facility antenatal care volume.

For qualitative data collection, all participants were purposively selected as follows: health providers in antenatal care clinics in the pilot health centres and health posts to include the head of the facility (two to three per health facility, approximately 60 in total for both rounds of data collection), together with district and provincial managers (approximately five in total); three to five pregnant women per health facility (approximately 100 in total) to include at least one primigravidae, two multigravidae, and a mix of first versus subsequent visits during the current pregnancy; community leaders to include representatives from religious leaders, tribal leaders, and kinship leaders (approximately ten in total); two to three husbands per health facility (approximately 25 in total) to include a mix of first-time fathers and experienced fathers; and one to two community health workers to include a mix of new and experienced community health workers to gain insights into different levels of experience and perspectives on implementing the pilot programme and supporting pregnant women (approximately ten in total; appendix 2 pp 4, 72–73).

Quantitative and qualitative data were synthesised to explore: (1) IPTp-DP delivery effectiveness and adherence including predictors (exit interviews and home visits); (2) coverage and fidelity, including any adaptations, and contextual factors influencing implementation (routine HMIS data, CQI data, and qualitative interviews); and (3) qualitative themes to help explain findings.

The coprimary outcomes were assessed in the modified intention-to-treat (mITT) population. The intention-to-treat (ITT) population defined for delivery effectiveness was all women who completed an exit interview, and for treatment adherence, all women who had a home visit. In the mITT population, women were excluded if they did not meet any of the initial eligibility criteria, or if they were not eligible for IPTp-DP according to guidelines (ie, had fever or malaria, had a positive malaria test, or because their IPTp-DP administration interval was less than the recommended 4 weeks). There was no safety analysis population.

Data were analysed using Stata 18. Descriptive data were presented as proportions for categorical variables and medians (IQR) for continuous variables. Predictors for delivery effectiveness (full *vs* partial or not effective) and adherence (full *vs* partial or non-adherence) were analysed using logistic regression with univariable (crude odds ratio [OR] and 95% CI) and multivariable (adjusted OR [aOR] and 95% CI) logistic regression models. To account for clustering at the facility level, we applied robust standard errors clustered at facility level, which adjusted 95% CIs and p values to reflect within-cluster correlation. Initially, delivery effectiveness was assessed at the cluster level, whereas adherence was analysed at the individual level. However, in post-hoc analysis, both delivery effectiveness and adherence were assessed at the cluster level to ensure consistency in the analytical approach. Additionally, adherence was assessed across all three levels (full adherence, partial adherence, and non-adherence) among participants with full and partial delivery effectiveness.

We did not adhere to the protocol-specified predictor analyses. Potential predictors included in the models were based on theoretical relevance drawn from previous literature, data availability, and observed variability within the study population. The modelling used an iterative stepwise selection method that adds or removes covariates from the model on the basis of their statistical significance, their effect size, whether they affect the intervention effect or its precision, and model fit following the Akaike information criterion (AIC). Variables considered for the initial full model included those associated with the endpoint with p values of 0·2 or less or effect sizes greater than 1·10 or less than 0·90 at univariable analyses. The final model retained variables with p values less than 0·05 and variables whose retention improved the model according to their AIC value. For effective delivery, variables considered were age group, education, ethnic group (Papuan *vs* non-Papuan, referring to the indigenous inhabitants who are typically Melanesian), marital status, gestational age, previous malaria test within past 28 days, had a previous course of IPTp-DP, health insurance ownership, and urban or semi-urban location of health facility. Socioeconomic status (based on self-reported household expenditure per capita divided into below or above the poverty line for Papua),^20^ religion, gravidity, number of antenatal care visits, has an illness at antenatal care visit, and side-effects taking dihydroartemisinin–piperaquine previously were excluded by the model. For adherence, variables considered were marital status, gestational age, antenatal care visit, and full or partial effective delivery. Age group, education, socioeconomic status, ethnic group, religion, gravidity, acceptance of IPTp-DP, side-effects of taking dihydroartemisinin–piperaquine previously, first antenatal care visit, has an illness at antenatal care visit, previous malaria test within past 28 days, had a previous course of IPTp-DP, health insurance ownership, and urban or semi-urban location of health facility were excluded by the model. We conducted post-hoc analyses, including comparing full or partial effective delivery versus non-effective delivery, and p_interaction_ values between selected variables.

The coverage of IPTp-DP courses delivered was estimated from HMIS across all ten facilities using routine data for the 12-month period Dec 1, 2022, to Nov 22, 2023. Data from the months when IPTp-DP was halted before this period were excluded. Coverage was calculated using the formula:

$${IPTp\;coverage}_{i}=\frac{{IPTp}_{i}}{K1}\times 100$$

where ${IPTp}_{i}$ is number of IPTp-DP courses administered and K1 is the number of first antenatal care visits.^21^

In the exit interviews and home visits, we reported the proportion of women who had received fewer than three courses of IPTp-DP versus those who received three or more courses. These proportions were presented as: (1) the number of women receiving IPTp-DP by course among women exiting antenatal care, and (2) the number of women receiving IPTp-DP by course among women who received fully or partially effective IPTp-DP delivery and were followed up at home, respectively.

CQI analysis to understand context assessed four of the five-step SQALE processes: (1) monitor (IPTp-DP coverage and implementation progress); (2) improve (targeted activities to enhance service delivery and adherence); (3) define (causes of low coverage), and (4) plan (strategies developed to address these root causes). Analysis prioritised ongoing improvement through real-time monitoring and problem-solving during the workshops.^17^

For qualitative analyses, transcripts of interviews were translated into English and data were coded deductively using the WHO health system building blocks^22^ (health providers and managers) and Sekhon’s acceptability of health-care interventions framework^23^ (pregnant women). Themes and subthemes were added inductively as they emerged. Thematic framework analysis identified key drivers of effective delivery and adherence to IPTp-DP. A more in-depth thematic analysis of the qualitative data has been reported separately.

### Role of the funding source

The funder of the study had no role in study design, data collection, data analysis, data interpretation, or writing of the report.

## Results

Between June 8, 2022, and Dec 27, 2023, we enrolled 1420 pregnant women in exit interviews, of whom 490 (34·5%) were visited at home (figure). 1366 women who were interviewed and 484 women who were visited at home were eligible for the delivery effectiveness and adherence analyses, respectively (mITT population). The characteristics of pregnant women are provided in table 1. Health facility characteristics, health-care provider characteristics, and acceptability of IPTp-DP among pregnant women in exit interviews are provided in the appendix 2 (pp 5–11).

**Figure:**
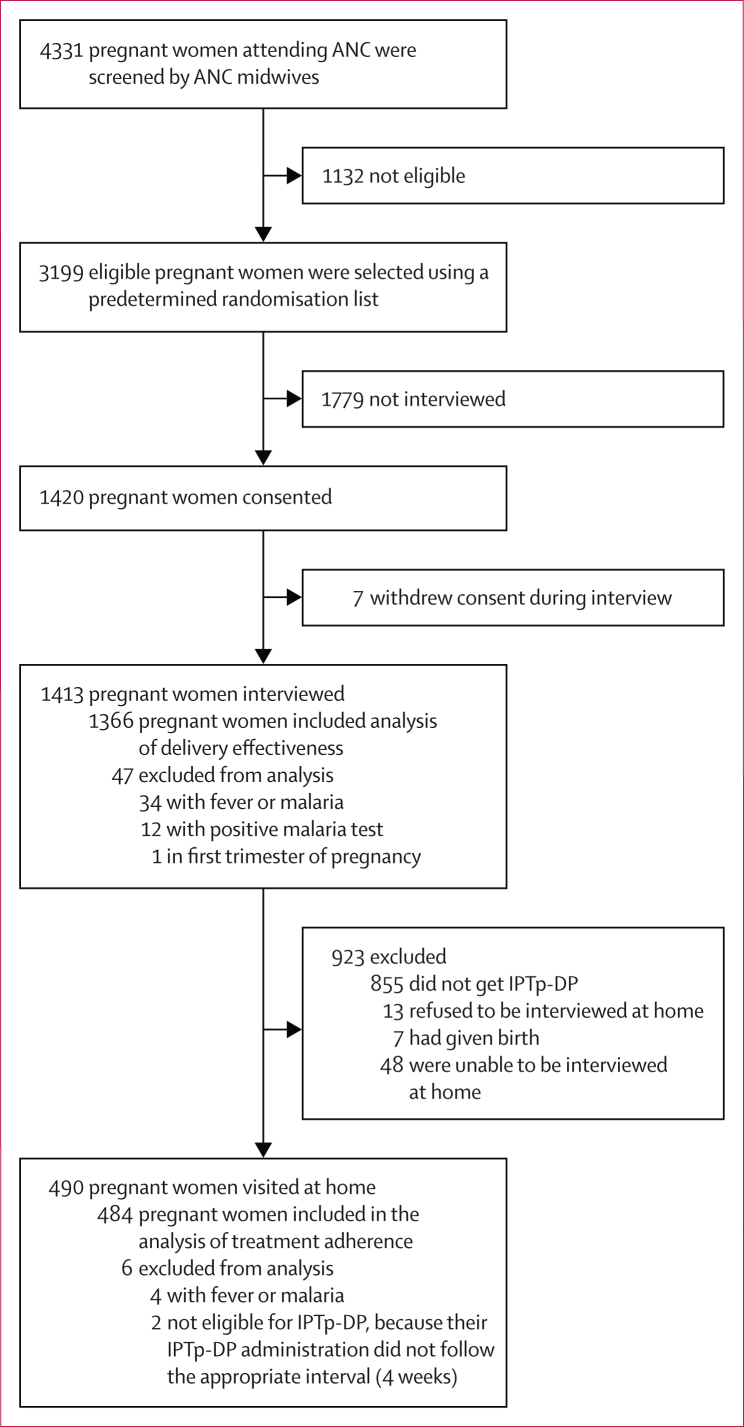
Study profile Due to operational constraints and the fact that initial eligibility screening was conducted by antenatal care midwives (before research team involvement), detailed records of screening exclusions at the antenatal care level were not available. Therefore, detailed exclusion data begin from the point of consent and research-team verification. ANC=antenatal care. IPTp-DP=intermittent preventive treatment with dihydroartemisinin–piperaquine.

**Table 1:** Characteristics of pregnant women at exit interview and home visit, modified intention-to-treat population

	Women who had an exit interview (N=1366)	Women who had a home visit (N=484)
Age (years)	27 (23–32)	27 (23–32)
Age group (years)		
15–19	101 (7%)	49 (10%)
20–34	1061 (78%)	354 (73%)
≥35	204 (15%)	81 (17%)
Weight (kg)	59 (52–68)	59 (51–65)
IPTp courses	0 (0–2)	2 (2–3)
Marital status		
Single, divorced, or widowed	86 (6%)	48 (10%)
Married	1280 (94%)	436 (90%)
Highest education completed		
No education or primary school	151/1314 (12%)	72/462 (16%)
Middle school or high school	872/1314 (66%)	305/462 (66%)
Diploma or university	291/1314 (22%)	85/462 (18%)
Ethnic group		
Non-Papuan	1007 (74%)	332 (69%)
Papuan	359 (26%)	152 (31%)
Religion		
Catholic or Protestant	860 (63%)	310 (64%)
Islamic or other	504 (37%)	174 (36%)
Health insurance ownership		
No	395 (29%)	167 (35%)
Yes	971 (71%)	317 (65%)
Socioeconomic status∗		
Above poverty line	273 (20%)	96 (20%)
Below poverty line	1093 (80%)	388 (80%)
Gravidity		
Primigravidae	414 (30%)	148 (31%)
Multigravida	952 (70%)	336 (69%)
Number of children who are currently younger than 5 years, excluding women experiencing their first pregnancy		
None	321/952 (34%)	114/336 (34%)
One	534/952 (56%)	185/336 (55%)
Two	80/952 (8%)	28/336 (8%)
Three or more	17/952 (2%)	9/336 (3%)
ANC visit		
One	4 (<1%)	0
Two	333 (24%)	128 (26%)
Three	267 (20%)	91 (19%)
Four or more	762 (56%)	265 (55%)
IPTp courses (previous and administered during antenatal care at the time of the exit interview)		
None	693 (51%)	0
One	71 (5%)	1 (<1%)
Two	373 (27%)	311 (64%)
Three	127 (9%)	92 (19%)
Four	58 (4%)	44 (9%)
Five	30 (2%)	25 (5%)
Six	14 (1%)	11 (2%)
Gestational age		
Second trimester	784 (57%)	302 (62%)
Third trimester	582 (43%)	182 (38%)
Illness was reason for visit		
No illness (routine ANC)	1329 (97·3%)	474 (98%)
Has illness	37 (2·7%)	10 (2%)
Payment for malaria test		
No or not sure	1362 (100%)	484 (100%)
Yes	4 (<1%)	0
Other payment		
No or not sure	1228 (90%)	442 (91%)
Yes	138 (10%)	42 (9%)
Received SST during pregnancy		
No	428/1364 (31%)	159 (33%)
Yes	936/1364 (69%)	325 (67%)
Previous malaria test in past 28 days		
No previous test	801 (59%)	299 (62%)
Had previous test	565 (41%)	185 (38%)
Previous malaria test result in past 28 days†		
Negative	541/563 (96%)	175/185 (95%)
Positive	22/563 (4%)	10/185 (5%)
Health facility		
One	3 (<1%)	2 (<1%)
Two	57 (4%)	13 (3%)
Three	98 (7%)	31 (6%)
Four	41 (3%)	30 (6%)
Five	70 (5%)	19 (4%)
Six	122 (9%)	89 (18%)
Seven	289 (21%)	68 (14%)
Eight	266 (19%)	86 (18%)
Nine	167 (12%)	93 (19%)
Ten	253 (19%)	53 (11%)
Location‡		
Urban	975 (71%)	300 (62%)
Semi-urban	391 (29%)	184 (38%)

Data are median (IQR), n (%), or n/N (%). Unless otherwise specified, different denominators indicate data were missing for some participants. ANC=antenatal care. IPTp=intermittent preventive treatment. SST=single screening and treatment.

∗ Based on household expenditure, which was ascertained through self-reported monthly household spending.

† Results of pregnant women who had previous test in past 28 days.

‡ Semi-urban locations are health facilities one, two, three, four, five, and six.

Overall, 556 (41%) of 1366 pregnant women had effective delivery of IPTp-DP. Specifically, 402 (29%) of 1366 women received full effective delivery and 154 (11%) received partial effective delivery (table 2). Of those with full effective delivery, 371 (92%) of 402 women received the first three tablets by DOT during consultation and six tablets to take home, and 31 (8%) received the first three tablets by DOT and zero tablets at antenatal care and were provided further doses during follow-up contacts. Of the 1366 pregnant women who were interviewed, 810 (59%) did not receive effective delivery. Of those who did not receive effective delivery, 534 (66%) of 810 women were not offered IPTp-DP and 235 (29%) received either inadequate or no doses to take.

**Table 2:** IPTp-DP delivery effectiveness among pregnant women with exit interviews, modified intention-to-treat population

	Pregnant women with exit interviews (N=1366)
Full delivery effectiveness (with DOT)∗	402 (29%)
Three DP tablets via DOT plus six DP tablets to take home	371 (27%)
Three DP tablets via DOT plus follow-up contacts on days 2–3 to deliver a further six DP tablets	31 (2%)
Partial delivery effectiveness (without DOT)†	154 (11%)
Nine DP tables to take home	151 (11%)
Three DP tablets to take home plus follow-up contacts on days 2–3 to deliver a further 6 DP tablets	3 (<1%)
Non-effective delivery‡	810 (59%)
With DOT and inadequate doses to take home	1 (<1%)
Without DOT and inadequate or no doses to take home	234 (17%)
Health workers did not offer IPTp	534 (39%)
Administer IPTp-DP to pregnant women who are not yet eligible due to timing that does not comply with the guidelines	41 (3%)

Data are n (%). Effective delivery was calculated as the proportion of women attending ANC treated appropriately according to the implementation of IPTp-DP guidelines; an appropriate dose of IPTp-DP is defined as the correct dose of three tablets per day for three days (ie, a total of nine tablets). ANC=antenatal care. DOT=directly observed therapy. DP=dihydroartemisinin–piperaquine. IPTp=intermittent preventive treatment. IPTp-DP=IPTp with DP.

∗ Full effective delivery is defined as the administration of three tablets under DOT for the first dose, followed by six tablets for subsequent doses.

† Partial effective delivery is defined as the receipt of all nine tablets without DOT.

‡ Any other scenario is classified as non-effective delivery.

437 (90%) of 484 women who had an eligible home visit reported full adherence, completing the 3-day regimen. 27 (6%) of 484 women reported partial adherence and 20 (4%) reported non-adherence (table 3). Among women who had an eligible home visit, 365 (75%) of 484 had full delivery effectiveness and 119 (25%) had partial delivery effectiveness.

**Table 3:** Adherence to IPTp-DP among pregnant women who had home visits, modified intention-to-treat population

	Pregnant women with full delivery effectiveness (N=365)	Pregnant women with partial delivery effectiveness (N=119)	Pregnant women with effective delivery (N=484)
Full adherence	339 (93%)	98 (82%)	437 (90%)
Partial adherence	14 (4%)	13 (11%)	27 (6%)
Non-adherence	12 (3%)	8 (7%)	20 (4%)

Data are n (%). The corresponding univariable and multivariable regression analyses of adherence predictors, comparing full adherence with partial or non-adherence, are available in the appendix 2 (pp 16–18). IPTp-DP=intermittent preventive treatment with dihydroartemisinin–piperaquine.

Multivariable analyses of predictors for full versus partial or non-effective delivery are shown in the appendix 2 (pp 12–14). Compared with women aged 20–34 years, full versus partial or non-effective delivery was more likely among women aged 35 years or older (aOR 1·26 [95% CI 1·04–1·51], p=0.0017), and no difference was seen with those aged 15–19 years (1·47 [0·66–3·26]; p=0·34). Compared with women with diploma or university education, full versus partial or non-effective delivery was more likely among those with no formal or only primary school education (2·01 [1·08–3·75], p=0·028) and no difference was seen with those with middle or high school education (1·30 [0·99–1·72, p=0·063]). Compared with women in the third trimester, full effective delivery, versus partial or non-effective delivery, was more likely among those in the second trimester (3·13 [2·11–4·63], p<0·0001). Women with a previous course of IPTp-DP were significantly more likely to receive full effective delivery compared to those without (4·30 [3·07–6·01], p<0·0001) and women without health insurance had higher odds of receiving full effective delivery than those with health insurance (1·33 [1·09–1·63], p=0·0044). No differences in the odds of full versus partial or non-effective delivery were seen according to ethnicity, marital status, previous malaria test within past 28 days, and urban or semi-urban location (appendix 2 pp 12–14). Additional post-hoc analyses of determinants of delivery effectiveness between full or partial delivery versus non-effective delivery are provided in the appendix 2 (pp 15–17).

Multivariable analyses of predictors for full adherence versus partial or non-adherence are shown in the appendix 2 (pp 18–20). Full adherence versus partial or non-adherence was more likely in women who were married compared with those who were single, divorced, or widowed (aOR 3·50 [95% CI 1·55–7·89], p=0·0028). Full adherence was also more likely in those who had attended four or more antenatal care visits versus those who had attended three or fewer visits (1·95 [1·22–3·13], p=0.0054). Additionally, women who received full effective delivery versus partial effective delivery were more likely to have full adherence (3·18 [1·82–5·54], p<0·0001). No differences in the likelihood of full adherence versus partial or non-adherence were seen by gestational age (appendix 2 pp 18–20).

The analysis of routine data highlighted varied performance across the ten health centres. Overall, across facilities from Dec 1, 2022, to Nov 22, 2023, among women attending their first antenatal care visit 1630 (42·7%) of 3815 received one course of IPTp-DP, 949 (24·9%) received two courses, and 880 (23·0%) received three or more courses. The highest performing health facility—facility four—had a coverage of 123 (103%) of 120 women attending their first antenatal care visit who received one course of IPTp-DP, 77 (64%) who received two courses, and 108 (90%) who received three or more courses. By contrast, the lowest performing facility—facility two—recorded 31 (19%) of 164 women attending their first antenatal care visit who received one course of IPTp-DP, ten (6%) who received two courses, and eight (5%) who received three or more courses.

CQI data provided information about implementation bottlenecks and mitigations that emerged, revealing that actions to address community reluctance and insufficient family support and resources varied by facility. Notably, coverage of the first course of IPTp-DP increased during periods when CQI workshops were held (appendix 2 p 23). Facility four initially reported no substantial barriers during the first CQI workshop but later identified declining coverage of three or more courses of IPTp-DP due to women delaying their antenatal care visits, limited family support in decision- making (ie, insufficient involvement or even refusal, particularly from husbands), and high population mobility (facility-level data are shown in the appendix 2 pp 24–33). Strategies to address these issues included personalised patient education, door-to-door visits for doses two and three with monitoring of side-effects, and close coordination with village midwives to improve the identification of pregnant women in the early stages of antenatal care and encourage timely IPTp-DP uptake. Facility two implemented targeted education campaigns to raise awareness among health workers, engaged trusted local midwives, and involved community leaders in the programme (appendix 2 pp 24–33).

Our assessment of implementation fidelity during CQI showed that local adaptations were made at some health facilities regarding the timing of when SST and IPTp-DP were delivered (appendix 2 pp 24–33). At the start of the pilot, health workers continued to test women for malaria if their first antenatal care visit was in the second or third trimester and treated them with dihydroartemisinin–piperaquine regardless of the test result. This practice deviated from the DHO delivery model, which stipulates that SST should be given in the first trimester, whereas IPTp-DP should be administered in the second and third trimester regardless of whether it is their first ANC visit. To address this deviation, the DHO re-emphasised the delivery model through a circular letter in late February, 2022. By December, 2022, health provider practices were aligned with the decree. However, the first dose was not consistently administered by DOT at the health facility due to a combination of provider-side challenges and patient-related factors. Innovations to ensure adherence included follow-up contact, through which health workers visited patients’ homes to deliver the subsequent doses. Adherence to doses given at the facility was also reinforced through telephone reminders.

Qualitative themes related to the delivery effectiveness of IPTp-DP by DOT and adherence are provided in the appendix 2 (pp 21–22). Delivery effectiveness was partly influenced by pregnant women’s hesitancy to take up IPTp-DP, which was exacerbated by challenges in health providers’ communication skills and insufficient engagement of husbands in the process. At health facility two, health providers reported not offering IPTp-DP at all after a community wide rejection of the programme since the beginning of the pilot implementation. Insufficient monthly IPTp-DP courses were attributed to late antenatal care initiation (ie, first antenatal visit attended in third trimester) and women giving birth before they were able to take up additional monthly courses of IPTp-DP. Additionally, supply chain issues, such as dihydroartemisinin–piperaquine stockouts, undermined women’s trust in the IPTp-DP programme. Side-effects of dihydroartemisinin–piperaquine were a notable challenge raised by health providers. Data from HMIS indicate that a total of 169 side-effects were reported. Some individuals might have had one or more side-effects, and potentially on one or more occasions. Among these, nausea was reported in 60 (36%) cases, vomiting in 49 (29%) cases, and dizziness in 30 (18%). However, both health providers and women reported that snacks provided at health facilities before taking dihydroartemisinin–piperaquine helped mitigate side-effects, reinforcing trust in the programme.

Adherence to IPTp-DP was thought to be driven by several factors. Both health providers and pregnant women noted that women were more likely to complete their doses at home if they were monitored regularly and provided with clear communication about the importance of IPTp-DP. Health-care providers and pregnant women emphasised that family support, particularly from husbands, played a crucial role in encouraging adherence. Full effective delivery also boosted adherence, because women said they valued being monitored during DOT and having side-effects managed by health-care providers.

## Discussion

To our knowledge, this is the first study to evaluate the delivery effectiveness of, and adherence to, IPTp-DP within a routine antenatal care setting in Papua, Indonesia. Adherence to the multiday dihydroartemisinin–piperaquine regimen for IPTp was higher than anticipated despite several challenges faced with IPTp-DP delivery. However, although adherence was much higher than estimated, improving power, the sample size was not met and therefore the adherence outcome should be interpreted with caution. Coverage of the IPTp-DP, particularly with two and three or more courses—based only on women attending antenatal care and not the wider population of pregnant women—was relatively low, which is to be expected so soon after the introduction of a new intervention. CQI data highlighted multiple operational challenges, such as drug stockouts and limited staff capacity, which affected IPTp-DP delivery and provided a mechanism for corrective action. The increased coverage in the number of women who received the first and second courses of IPTp-DP observed following consecutive CQI workshops probably reflects enhanced accountability because facilities were required to present, review, and reflect on their own performance, which in turn motivated action. Qualitative findings pointed to late antenatal care initiation, women’s concerns over side-effects, and the positive influence of husbands as key factors influencing adherence, issues which were also identified and addressed during CQI workshops. As a result of the findings from this study and the nested studies reported elsewhere, the Indonesian Government has adopted IPTp-DP as policy in areas with an annual parasite index of more than 100 in Indonesia.^24^

This pilot implementation of IPTp-DP in Papua presented unique challenges unlike those associated with IPTp-SP delivery. The use of dihydroartemisinin–piperaquine for IPTp adds complexity to the national programme, because it is also the first-line treatment for malaria in Indonesia. During the extended stockout period, the MOH rightly prioritised dihydroartemisinin–piperaquine for life-saving treatment over prevention. The stockout had a detrimental effect on coverage once delivery resumed, remaining lower in the post-stockout period than at the start of the pilot. The break in service delivery undermined women’s trust in the programme. To minimise bias, analysis of coverage trends was restricted to the 12-month period following the resumption of supply, and the main outcomes on delivery and adherence were measured at least 7 months after stock had stabilised. Furthermore, the 3-day regimen of dihydroartemisinin–piperaquine is inherently more complex than a single-dose sulphadoxine–pyrimethamine regimen. Although the pilot adopted a simplified regimen (a fixed dose of three tablets per day for 3 days^25^ instead of weight-based dosing), delivery remained a challenge, as evidenced by the low delivery effectiveness and low coverage with three or more courses. This complexity was also shown with IPTp-SP. Indeed, coverage of IPTp-SP remains low in Kenya even after decades of implementation.^19^

The effectiveness of IPTp-DP delivery in Papua was influenced by several factors at individual and health-care system levels. At the individual level, our findings showed that having no formal education or only primary school education was associated with higher IPTp-DP uptake, when compared with diploma or university level education. A study in Mali similarly found that women with higher than primary-level education were less likely to receive IPTp-SP than women with no education.^26^ However, this finding contrasts with findings from a systematic review of IPTp-SP in sub-Saharan Africa, in which higher education levels were shown to be associated with increased uptake.^27^

Adherence to IPTp-DP in our study was higher than adherence reported in the clinical trial conducted in Papua.^8^ In our qualitative analysis, we found that frequent antenatal care visits and family support (particularly from husbands and male partners) had a crucial role in boosting adherence, which is consistent with evidence on the role of partner involvement in malaria prevention.^28^ In addition, directly observing the first dose supported adherence by giving health-care workers the opportunity to provide advice to women. Women who felt cared for by health-care providers said they were more likely to complete the 3-day regimen. These findings suggest that comprehensive communication and support can be essential in facilitating adherence to complex regimens.^29^ Our findings reflect the need for flexibility in service delivery, allowing for adaptations such as visiting antenatal care clients at home to deliver doses (practised by one facility) or reminders using phone or video calls, which would be more feasible and scalable in busy clinics.

Although provider concerns about IPTp-DP were prominent in the previous clinical trial,^10^ in the context of this pilot, which was led and implemented by the MOH, we found providers were more positive towards IPTp-DP. The primary concerns of providers were pregnant women's acceptability because IPTp-DP is given presumptively. By contrast, SST involves prior testing, which women were familiar with. Indeed, hesitation about taking the drug without a confirmed malaria diagnosis was expressed by some women and husbands. This hesitation stemmed from the prevailing practice of testing before treating. This practice meant that pregnant women and health providers preferred to receive malaria treatment only after a confirmed malaria diagnosis, which was also noted in our previous study.^10^ This preference underscores the need for further training to ensure that health-care providers understand the preventive nature of IPTp-DP and can effectively communicate its benefits and address women’s concerns.

This study has several limitations. We could not employ interpreters, so participants who could not speak Bahasa Indonesian were excluded. Self-reported measures of service delivery and assessment of adherence could have led to an overestimation of study outcomes. However, we mitigated this overestimation by observing any medicines prescribed to women at antenatal care visits during the exit interviews and by doing pill counts during home visits. The collection of routine data at health facilities probably had a positive effect on the results observed. Furthermore, the sample size for adherence was smaller than anticipated due to lower-than-expected full delivery effectiveness, which reduced precision and means that findings should be interpreted with caution. The observed ICC was higher than anticipated, with values of 0·58 for effective delivery, and 0·06 compared with the expected 0·03 for adherence. This difference for adherence might have reduced statistical power, particularly affecting non-significant associations. Among those who received IPTp-DP, adherence rates were high, resulting in only a modest number of non-adherent or partial adherent cases for the comparison group in regression models. Importantly, the study was conducted as part of a pilot under real-world conditions, in which health-care workers faced typical resource constraints, including staff shortages, limited drug supplies, and poor quality of HMIS data. Lastly, the pilot was restricted to urban and semi-urban settings, thus the findings might not reflect challenges faced in more remote rural areas, affecting the generalisability of the results.

In conclusion, the implementation of IPTp-DP during routine antenatal care is feasible and adherence to the 3-day regimen was high. Although coverage was a challenge in the pilot, strategies to address these have been identified by stakeholders. This study supports the expansion of IPTp-DP in other regions in Indonesia and other countries in the region with high malaria endemicity.

## Data sharing

The data sharing agreement is available in the appendix 2 (p 90).

## Declaration of interests

HF is an employee of the Liverpool School of Tropical Medicine (LSTM; Liverpool, UK) and received support from the UK Medical Research Council (MRC) and LSTM for travel and attending meetings. All other authors declare no competing interests.

## References

[1] Reddy V, Weiss DJ, Rozier J, Ter Kuile FO, Dellicour S (2023). Global estimates of the number of pregnancies at risk of malaria from 2007 to 2020: a demographic study. Lancet Glob Health.

[2] WHO (2024).

[3] Moore KA, Fowkes FJI, Wiladphaingern J (2017). Mediation of the effect of malaria in pregnancy on stillbirth and neonatal death in an area of low transmission: observational data analysis. BMC Med.

[4] Rijken MJ, McGready R, Boel ME (2012). Malaria in pregnancy in the Asia-Pacific region. Lancet Infect Dis.

[5] Webster J, Ansariadi, Burdam FH (2018). Evaluation of the implementation of single screening and treatment for the control of malaria in pregnancy in Eastern Indonesia: a systems effectiveness analysis. Malar J.

[6] WHO (2023). WHO guidelines for malaria. https://iris.who.int/.

[7] Siswantoro H, Ratclif A, Kenangalem E (2006). Efficacy of existing antimalarial drugs for uncomplicated malaria in Timika, Papua, Indonesia. Med J Indones.

[8] Ahmed R, Poespoprodjo JR, Syafruddin D (2019). Efficacy and safety of intermittent preventive treatment and intermittent screening and treatment versus single screening and treatment with dihydroartemisinin-piperaquine for the control of malaria in pregnancy in Indonesia: a cluster-randomised, open-label, superiority trial. Lancet Infect Dis.

[9] Paintain L, Hill J, Ahmed R (2020). Cost-effectiveness of intermittent preventive treatment with dihydroartemisinin-piperaquine versus single screening and treatment for the control of malaria in pregnancy in Papua, Indonesia: a provider perspective analysis from a cluster-randomised trial. Lancet Glob Health.

[10] Hoyt J, Landuwulang CUR, Ansariadi (2018). Intermittent screening and treatment or intermittent preventive treatment compared to current policy of single screening and treatment for the prevention of malaria in pregnancy in Eastern Indonesia: acceptability among health providers and pregnant women. Malar J.

[11] Gutman J, van Eijk AM, Rodriguez E, Ahn J, ter Kuile FO (2022).

[12] Phillips WR, Sturgiss E, Glasziou P (2023). Improving the reporting of primary care research: consensus reporting items for studies inprimary care-the CRISP statement. Ann Fam Med.

[13] Ryan N, Vieira D, Gyamfi J (2022). Development of the ASSESS tool: a comprehensive tool to support reporting and critical appraisal of qualitative, quantitative, and mixed methods implementation research outcomes. Implement Sci Commun.

[14] Indonesia Ministry of Health (2024). Kasus Malaria di Indonesia Periode 2023. https://malaria.kemkes.go.id/case.

[15] Poespoprodjo JR, Kenangalem E, Wafom J (2018). Therapeutic response to dihydroartemisinin-piperaquine for *P falciparum* and *P vivax* nine years after its introduction in southern Papua, Indonesia. Am J Trop Med Hyg.

[16] Hoffmann TC, Glasziou PP, Boutron I (2014). Better reporting of interventions: template for intervention description and replication (TIDieR) checklist and guide. BMJ.

[17] Otiso L, Karuga R (2016). SQALE: putting quality at the heart of community health. http://usaidsqale.reachoutconsortium.org/.

[18] Permala J, Tarning J, Nosten F, White NJ, Karlsson MO, Bergstrand M (2017). Prediction of improved antimalarial chemoprevention with weekly dosing of dihydroartemisinin-piperaquine. Antimicrob Agents Chemother.

[19] Dellicour S, Hill J, Bruce J (2016). Effectiveness of the delivery of interventions to prevent malaria in pregnancy in Kenya. Malar J.

[20] BPS (2024). Garis Kemiskinan Menurut Kabupaten/Kota (Rupiah/Capita/Month). 2024/12/18. https://www.bps.go.id/en/statistics-table/2/NjI0IzI=/garis-kemiskinan-menurut-kabupaten-kota--rupiah-capita-month-.html.

[21] WHO (2020).

[22] WHO (2010).

[23] Sekhon M, Cartwright M, Francis JJ (2017). Acceptability of healthcare interventions: an overview of reviews and development of a theoretical framework. BMC Health Serv Res.

[24] Ministry of Health of the Republic of Indonesia (2024). National action plan for acceleration of malaria elimination 2020-2026 (revision). https://mesamalaria.org/resource-hub/national-malaria-strategic-plan-nmsp-of-indonesia-2020-2026/.

[25] Savic RM, Jagannathan P, Kajubi R (2018). Intermittent preventive treatment for malaria in pregnancy: optimization of target concentrations of dihydroartemisinin-piperaquine. Clin Infect Dis.

[26] Webster J, Kayentao K, Bruce J (2013). Prevention of malaria in pregnancy with intermittent preventive treatment and insecticide treated nets in Mali: a quantitative health systems effectiveness analysis. PLoS One.

[27] Darteh EKM, Dickson KS, Ahinkorah BO (2021). Factors influencing the uptake of intermittent preventive treatment among pregnant women in sub-Saharan Africa: a multilevel analysis. Arch Public Health.

[28] Chepkemoi Ng’etich Mutulei A (2013). Factors influencing the uptake of intermittent preventive treatment for malaria in pregnancy: evidence from Bungoma east district, Kenya. Am J Public Health Res.

[29] Barsosio HC, Webster J, Omiti F (2024). Delivery effectiveness of and adherence to intermittent preventive treatment for malaria in pregnancy with dihydroartemisinin-piperaquine with or without targeted information transfer or sulfadoxine-pyrimethamine in western Kenya: a three-armed, pragmatic, open-label, cluster-randomised trial. Lancet Glob Health.

