# Supplementary material for: Pilot implementation of intermittent preventive treatment with dihydroartemisinin–piperaquine to prevent adverse birth outcomes in Papua, Indonesia: a mixed-method evaluation
Source: Lancet Prim Care. 2025 Jul;1(1):None. doi: 10.1016/j.lanprc.2025.100011 (PMC12379630; doi:10.1016/j.lanprc.2025.100011)
Supplement: Supplementary appendix 1 [file mmc1.pdf]

THE LANCET
Primary Care

Supplementary appendix 1

This translation in Bahasa was submitted by the authors and we reproduce it as supplied. It has not been peer reviewed. *The Lancet*'s editorial processes have only been applied to the original in English, which should serve as reference for this manuscript.

Terjemahan dalam Bahasa Indonesia ini diserahkan oleh penulis dan diproduksi kembali apa adanya tanpa melalui proses peer review. Proses editorial *Lancet* hanya diterapkan pada versi original dalam Bahasa Inggris, yang harus dijadikan rujukan untuk naskah ini.

Supplement to: Hafidz F, Candrawati F, Hoyt J, et al. Pilot implementation of intermittent preventive treatment with dihydroartemisinin–piperaquine to prevent adverse birth outcomes in Papua, Indonesia: a mixed-method evaluation. *Lancet Prim Care* 2025. https://doi.org/10.1016/j.lanprc.2025.100011

Abstrak

Latar Belakang Sebuah uji coba sebelumnya menunjukkan bahwa pengobatan pencegahan malaria secara berkala dengan *dihidroartemisinin–piperaquine* (IPTp-DP) lebih efektif dibandingkan kebijakan saat ini, yaitu skrining dan pengobatan malaria pada kunjungan pertama kehamilan (SST), dalam mencegah malaria selama kehamilan di Papua, Indonesia. Studi STOPMiP-2 mengevaluasi implementasi percontohan IPTp-DP oleh Kementerian Kesehatan melalui layanan rutin *antenatal* di Papua.

Metode Evaluasi *mixed-method* dilakukan di sepuluh fasilitas kesehatan primer di Kabupaten Mimika, Papua, Indonesia, dari 8 Juni 2022 hingga 27 Des 2023. Ibu hamil berusia 15–49 tahun dengan status HIV negatif (jika status diketahui), berada pada trimester kedua atau ketiga kehamilan, dan memberikan persetujuan tertulis dianggap memenuhi syarat. Efektivitas pemberian IPTp-DP (3 hari pemberian dengan tiga tablet per hari [total sembilan tablet], di mana dosis pertama diberikan dengan pengawasan secara langsung selama kunjungan *antenatal*) dan kepatuhan (mengonsumsi seluruh sembilan tablet, dikonfirmasi melalui perhitungan pil) merupakan dua luaran utama. Analisis dilakukan pada populasi *modified intention-to-treat* (mITT), yang didefinisikan sebagai semua ibu hamil yang menyelesaikan wawancara setelah layanan (untuk efektivitas pemberian), dan semua yang menjalani kunjungan rumah (untuk kepatuhan). Populasi mITT mengecualikan wanita dengan demam atau infeksi malaria, hasil tes malaria positif, atau yang menerima IPTp-DP di luar waktu yang ditentukan (yaitu, kurang dari 4 minggu antar siklus). Kami mengeksplorasi faktor-faktor prediktor efektivitas pemberian dan kepatuhan menggunakan regresi logistik multivariabel, serta menggunakan data kualitatif untuk memberikan penjelasan lebih lanjut. Data informasi kesehatan rutin juga digunakan untuk menilai cakupan bulanan berdasarkan fasilitas. Studi ini terdaftar di ClinicalTrials.gov (NCT05294406) dan telah selesai.

Temuan Dari 8 Juni 2022 hingga 27 Des 2023, kami merekrut 1420 ibu hamil untuk wawancara setelah layanan, dan 1366 data tersedia dan memenuhi syarat untuk analisis efektivitas. Sebanyak 490 wanita menjalani kunjungan rumah, dan 484 data tersedia serta memenuhi syarat untuk analisis kepatuhan. Sebanyak 556 (41%) dari 1366 wanita menerima pemberian IPTp-DP yang efektif, dan dari mereka yang memiliki data kepatuhan, 437 (90%) dari 484 menunjukkan kepatuhan penuh. Faktor prediktor efektivitas penuh dalam pemberian IPTp-DP dibandingkan efektivitas parsial atau tidak efektif meliputi: usia ibu lebih tua (≥35 tahun *vs* 20-34 tahun: *adjusted odds ratio* 1·26 [95% CI 1·04-1·51], p=0·017), pendidikan lebih rendah (tidak berpendidikan atau sekolah dasar *vs* diploma atau universitas: 2·01 [1·08-3·75], p=0·028), kehamilan trimester kedua (*vs* trimester ketiga: 3·13 [2·11–4·63], p<0·0001), memiliki riwayat menerima IPTp-DP (*vs* tidak pernah menerima: 4·30 [3·07-6·01], p<0·0001), dan tidak memiliki asuransi kesehatan (*vs* memiliki: 1·33 [1·09–1·63], p=0·0044). Tidak ditemukan perbedaan berdasarkan usia muda (usia 15–19 tahun), pendidikan menengah atau tinggi, etnis, status pernikahan, riwayat tes malaria dalam 28 hari terakhir, dan lokasi. Faktor prediktor kepatuhan penuh meliputi: status menikah (*vs* lajang, bercerai, atau janda: 3·50 [1·55-7·89], p=0·0028), menghadiri empat atau lebih kunjungan *antenatal* (*vs* kurang atau sama dengan tiga kunjungan: 1·95 [1·22-3·13], p=0·0054), dan efektivitas penuh dalam pemberian IPTp-DP (*vs* efektivitas parsial: 3·18 [1·82–5·54], p<0·0001). Tidak ditemukan perbedaan berdasarkan usia kehamilan. Antara 1 Des 2022 hingga 22 Nov 2023, di seluruh fasilitas, sebanyak 1630 (43%) dari 3815 wanita yang datang untuk kunjungan *antenatal* pertama menerima satu siklus IPTp-DP, 949 (25%) menerima dua siklus, dan 880 (23%) menerima tiga atau lebih siklus.

Interpretasi Di antara mereka yang menerima IPTp-DP, kepatuhan terhadap rejimen 3-hari IPTp-DP sangat tinggi. Namun, ukuran sampel untuk kepatuhan lebih kecil dari yang diharapkan akibat rendahnya efektivitas pemberian secara penuh. Studi mendatang perlu mengeksplorasi strategi untuk meningkatkan efektivitas pemberian dalam konteks ini.

Pendanaan UK Medical Research Council.
