# Supplementary material for: Pilot implementation of intermittent preventive treatment with dihydroartemisinin–piperaquine to prevent adverse birth outcomes in Papua, Indonesia: a mixed-method evaluation
Source: Lancet Prim Care. 2025 Jul;1(1):None. doi: 10.1016/j.lanprc.2025.100011 (PMC12379630; doi:10.1016/j.lanprc.2025.100011)
Supplement: Supplementary appendix 2 [file mmc2.pdf]

THE LANCET
Primary Care

Supplementary appendix 2

This appendix formed part of the original submission and has been peer reviewed.
We post it as supplied by the authors.

Supplement to: Hafidz F, Candrawati F, Hoyt J, et al. Pilot implementation of intermittent preventive treatment with dihydroartemisinin–piperaquine to prevent adverse birth outcomes in Papua, Indonesia: a mixed-method evaluation. *Lancet Prim Care* 2025. https://doi.org/10.1016/j.lanprc.2025.100011

Table of Contents

Figure S1 a) Mimika District on Papua Island, Indonesia); b) The ten study health facilities in Timika City. Source: Google (2022) 1

Figure S2 Timeline for IPTp-DP Pilot Implementation and Evaluation in Mimika District . 2

Researcher Training in study methods 3

Exit Interviews and Home Visits 3

Qualitative interviewers 3

Qualitative interviewers and respondent selection 4

Study results of effectiveness and adherence of IPTp-DP 5

Table S1 Health facility characteristics by urban and semi-urban setting (annual data for 2023) 5

Table S2 Healthcare worker characteristics by health facility 6

Table S3 Acceptability of IPTp-DP among pregnant women in exit interviews 10

Table S4 Predictors of delivery effectiveness comparing full vs partial/ non-effective delivery 12

Table S5 Predictors of delivery effectiveness comparing full/partial vs non-effective delivery (post hoc) 15

Table S6 Predictors of adherence comparing full vs partial/ non-adherence 18

Table S7 Factors influencing effectiveness of IPTp-DP delivery by DOT and adherence 21

Figures S3-13 Health facility performance data showing IPTp-DP coverage and CQI data 23

Algorithm of IPTp-DP services. Source: Mimika District Health Office (2022) 34

TIDieR (Template for Intervention Description and Replication) Checklist*: 35

CRISP checklist 43

ASSESS guidance 50

STOPMiP-2 study protocol 57

Data sharing statement 90

STOPMiP-2 statistical analysis plan 91

Map of the ten health facilities

Figure S1 a) Mimika District on Papua Island, Indonesia); b) The ten study health facilities in Timika City. Source: Google (2022)

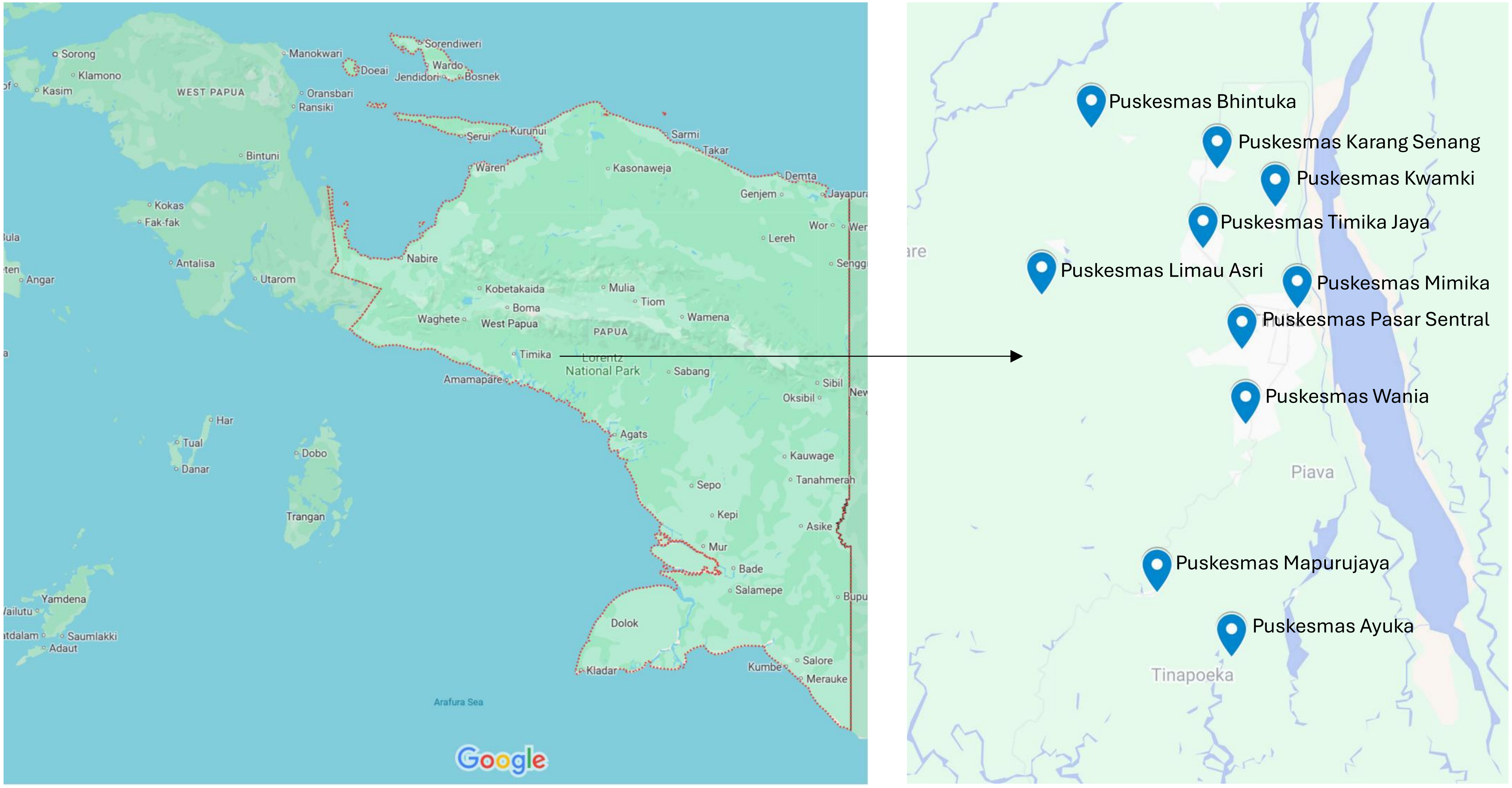

(a) Map data ©2024 Google 100 km (b)

Google. Map data Indonesia. 2022. https://www.google.co.id/ (accessed 10-05-2022 2022).

Timeline for IPTp-DP pilot implementation and evaluation in Mimika District

Figure S2 Timeline for IPTp-DP Pilot Implementation and Evaluation in Mimika District

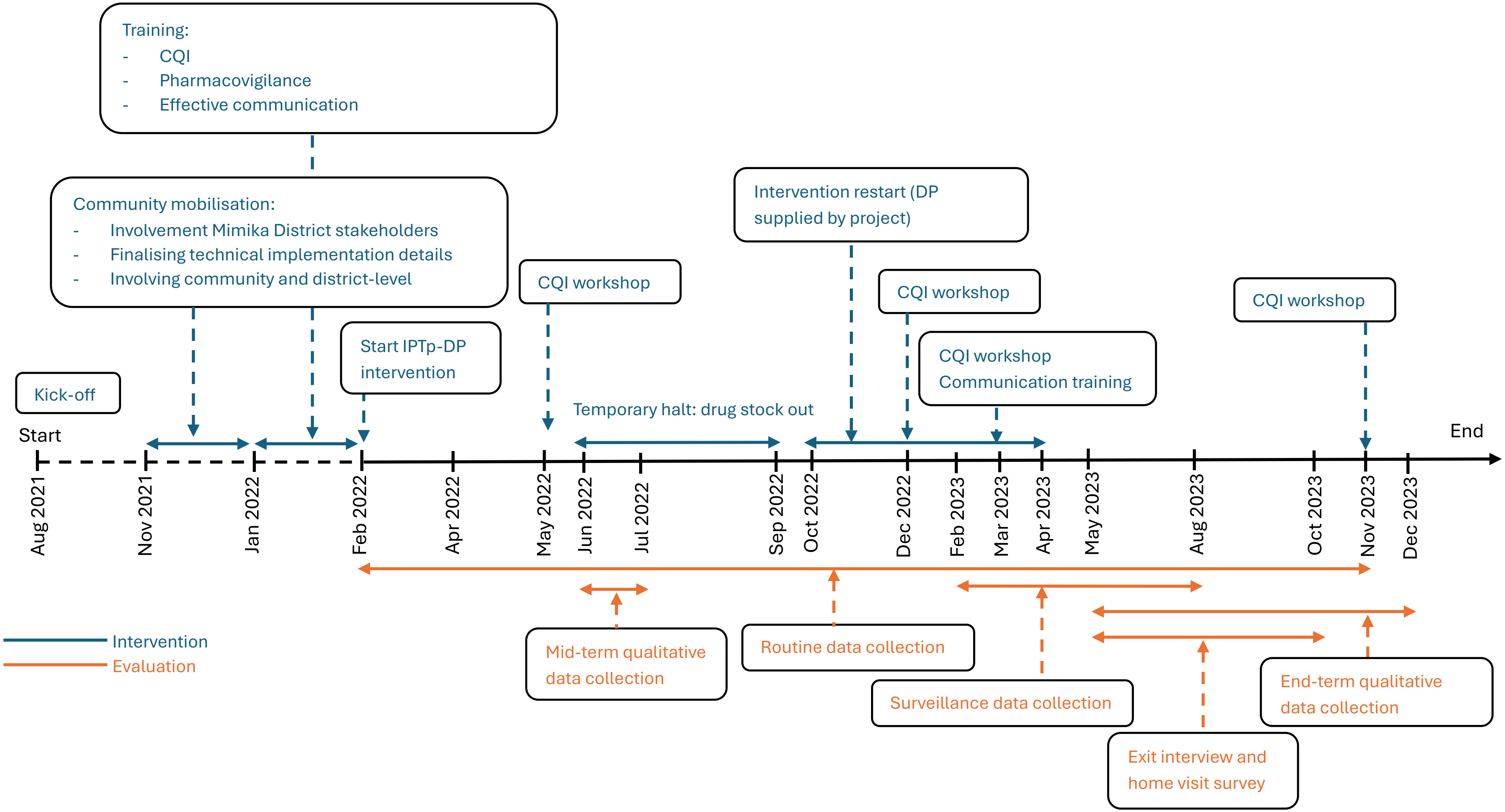

IPTp-DP: Intermittent Preventive Treatment in pregnancy with Dihydroartemisinin-Piperaquine; CQI: Continuous Quality Improvement

Researcher Training in study methods

Exit Interviews and Home Visits

Comprehensive training of data collectors for the exit interviews and home visits was conducted in two phases: 18–20 April and 25–28 April 2023, at Timika Research Team (YPKMP). The team comprised ten research midwives and nine fieldworkers recruited locally

The training introduced the study background and objectives, clarified field protocols, and reinforced ethical and safeguarding principles.

Key topics covered included:

- Overview of the IPTp-DP implementation study and expected study outcomes;
- Eligibility criteria and written informed consent procedures;
- Data collection tools for exit interviews and home visit interviews;
- Recording of pill counts and adherence monitoring;
- Good clinical practices and safeguarding procedures;
- Strategies for tracing participants and documenting outcomes accurately.

Staff also engaged in role-play and mock interviews to simulate field conditions. Trainers included members of the study team with experience in implementation research and malaria in pregnancy studies. The training concluded with a pilot to technically test and evaluate the procedures for conducting interviews.

Qualitative interviewers

A three-day training session on qualitative data collection was conducted for three locally recruited interviewers for in-depth interviews from 31 May to 4 June 2022. The training aimed to ensure consistency and quality in interviewing techniques, data collection, data management and security, data processing and transcription, with an additional module on ethical standards.

Qualitative interviewers training and respondent selection

Qualitative interviewers and respondent selection

Participant Selection Approach:
Participants were purposively selected. Pregnant women, their husbands, and community health workers were identified by midwives from pilot PHCs and referred to the study team. The team recruited them into the study based on the inclusion and exclusion criteria., and willingness to participate. Health providers were selected based on their role in IPTp-DP delivery, while health managers at district, provincial, and national level were selected based on policy roles. Interviews were scheduled at a convenient time and location for participants. Community leaders were identified and invited by the study team for focus group discussions.

Number of Participants
A total of 226 individuals were interviewed across midline and endline data collection.

Stakeholder Group	Mid-Term (n)	End-Term (n)	Total
Pregnant women	47	47	94
Health workers of PHC	29	40	69
Health facility managers (head of PHC)	10	10	20
District Health Office of Mimika staff	4	7	11
Provincial Health Office staff	0	1	1
Indonesia Ministry of Health staff	0	2	2
Community health workers	0	3	3
Husbands	0	13	13
Community leaders (via FGD)	0	13	13
**Total**	**90**	**136**	**226**

Study results of effectiveness and adherence of IPTp-DP

Table S1 Health facility characteristics by urban and semi-urban setting (annual data for 2023)

Characteristic	Semi urban							Urban					Average
Facility	Facility 4	Facility 6	Facility 1	Facility 5	Facility 3	Facility 2	Average	Facility 9	Facility 10	Facility 8	Facility 7	Average	All health facilities
Population coverage	14,369	11,000	1,730	10,000	10,000	35,704	13,801	19,298	14,706	68,084	36,000	34,522	22,089
Health facility satellite	2	2	1	4	2	2	2.2	-	2	2	-	1.0	2
Integrated health post	10	9	2	7	11	1	6.7	9	13	16	9	11.8	9
First ANC visit*	120	274	49	169	281	164	176.2	424	613	849	872	689.5	382
Total ANC visit*	453	1,064	133	904	1,132	557	707.2	1,919	3,327	3,639	3,221	3,026.5	1,635
Doctor involved in ANC	3	2	1	2	1	3	2.0	1	2	1	3	1.8	2
Midwives involved in ANC	22	7	6	5	6	11	9.5	2	8	4	22	9.0	9
Nurses involved in ANC	3	-	4	-	-	13	3.3	2	-	10	2	3.5	6
Nurse assistant involved in ANC	-	-	-	-	-	-	-	-	-	2	2	1.0	2
Other staff involved in ANC	-	-	1	-	-	9	1.7	-	-	1	1	0.5	3
Client of first ANC* to total ANC staff ratio	2	14	5	42	72	5	10.7	85	52	62	23	43.8	36
Ratio IPTp 1 to first ANC*	103%	79%	39%	44%	23%	19%	50%	67%	42%	33%	32%	40%	43%
Ratio IPTp 2 to first ANC*	64%	53%	20%	41%	14%	6%	33%	44%	20%	19%	14%	22%	25%
Ratio IPTp 3+ to first ANC*	90%	51%	31%	32%	17%	5%	35%	49%	20%	9%	11%	18%	23%
Number of IPTp-DP dose	308	504	44	198	154	49	209.5	676	508	520	498	482.3	346

*Annual data from 1 Dec 2022 to 22 Nov 2023 - average by region

Table S2 Healthcare worker characteristics by health facility

Characteristic				Semi urban					Urban		
	Facility 4	Facility 6	Facility 1	Facility 5	Facility 3	Facility 2		Facility 9	Facility 10	Facility 8	Facility 7
	**median (IQR)**	**median (IQR)**	**median (IQR)**	**median (IQR)**	**median (IQR)**	**median (IQR)**		**median (IQR)**	**median (IQR)**	**median (IQR)**	**median (IQR)**
Age (years)	33 (31-35.5)	35 (32-38)	32 (29.5-35.5)	36 (33.5-38)	43.5 (36-47)	31 (27.5-37)		37 (35.5-44)	42.5 (36.5-44)	41.5 (38-45)	36 (33.5-42)
Employment duration (years)	6.5 (4.5-10)	4.5 (3-9.5)	11 (5-19.5)	11.50 (9-13.5)	8 (6-10)	8 (5.50-8)		7.50 (6.5-15.50)	7.50 (6-9)	12 (8-16)	7 (6.5-7)
ANC days per week by midwife	4.5 (3-6)	4 (4-4)	2 (1-3)	6 (6-6)	6 (5-6)	4.50 (4-5)		4 (4-4)	6 (6-6)	5 (4-6)	5.50 (5-6)
Outreach days per month by midwife	14.5 (5-24)	5 (4-6)	9 (6-12)	15 (6-24)	18 (11-23)	5.50 (5-6)		3.50 (2-5)	15 (6-24)	3.50 (3-4)	27 (24-30)
	**n/N (%)**	**n/N (%)**	**n/N (%)**	**n/N (%)**	**n/N (%)**	**n/N (%)**		**n/N (%)**	**n/N (%)**	**n/N (%)**	**n/N (%)**
Type of health workers											
Midwife	2/4 (50%)	2/4 (50%)	2/4 (50%)	2/4 (50%)	4/6 (67%)	2/4 (50%)		2/4 (50%)	2/4 (50%)	2/4 (50%)	2/4 (50%)
Nurse	1/4 (25%)	1/4 (25%)	1/4 (25%)	1/4 (25%)	1/6 (17%)	1/4 (25%)		1/4 (25%)	1/4 (25%)	1/4 (25%)	1/4 (25%)
Pharmacist	1/4 (25%)	1/4 (25%)	1/4 (25%)	1/4 (25%)	1/6 (17%)	1/4 (25%)		1/4 (25%)	1/4 (25%)	1/4 (25%)	1/4 (25%)
Sex											
Female	3/4 (75%)	4/4 (100%)	3/4 (75%)	4/4 (100%)	6/6 (100%)	4/4 (100%)		4/4 (100%)	4/4 (100%)	4/4 (100%)	4/4 (100%)
Male	1/4 (25%)	0/4 (0%)	1/4 (25%)	0/4 (0%)	0/6 (0%)	0/4 (0%)		0/4 (0%)	0/4 (0%)	0/4 (0%)	0/4 (0%)
Ethnic											
non-Papua	4/4 (100%)	4/4 (100%)	4/4 (100%)	4/4 (100%)	4/6 (67%)	3/4 (75%)		3/4 (75%)	4/4 (100%)	4/4 (100%)	4/4 (100%)
Papua	0/4 (0%)	0/4 (0%)	0/4 (0%)	0/4 (0%)	2/6 (33%)	1/4 (25%)		1/4 (25%)	0/4 (0%)	0/4 (0%)	0/4 (0%)
Education											
Diploma	1/4 (25%)	3/4 (75%)	3/4 (75%)	2/4 (50%)	3/6 (50%)	3/4 (75%)		2/4 (50%)	0/4 (0%)	3/4 (75%)	1/4 (25%)
Bachelor	2/4 (50%)	1/4 (25%)	1/4 (25%)	2/4 (50%)	3/6 (50%)	1/4 (25%)		2/4 (50%)	3/4 (75%)	1/4 (25%)	3/4 (75%)
Master	1/4 (25%)	0/4 (0%)	0/4 (0%)	0/4 (0%)	0/6 (0%)	0/4 (0%)		0/4 (0%)	1/4 (25%)	0/4 (0%)	0/4 (0%)
Role in antenatal care											
History taking	2/4 (50%)	2/4 (50%)	2/4 (50%)	2/4 (50%)	4/6 (67%)	3/4 (75%)		2/4 (50%)	2/4 (50%)	2/4 (50%)	2/4 (50%)

Study results of effectiveness and adherence of IPTp-DP

Characteristic				Semi urban					Urban		
	Facility 4	Facility 6	Facility 1	Facility 5	Facility 3	Facility 2		Facility 9	Facility 10	Facility 8	Facility 7
Physical examination	2/4 (50%)	2/4 (50%)	2/4 (50%)	2/4 (50%)	3/5 (60%)	3/4 (75%)		2/4 (50%)	2/4 (50%)	2/4 (50%)	1/4 (25%)
Clinical investigation	2/4 (50%)	3/4 (75%)	2/4 (50%)	2/4 (50%)	3/5 (60%)	3/4 (75%)		2/4 (50%)	2/4 (50%)	2/4 (50%)	2/4 (50%)
Obstetric examination	2/4 (50%)	2/4 (50%)	2/4 (50%)	2/4 (50%)	3/5 (60%)	3/4 (75%)		2/4 (50%)	2/4 (50%)	2/4 (50%)	2/4 (50%)
Drug dispensing	3/4 (75%)	3/4 (75%)	2/4 (50%)	3/4 (75%)	2/5 (40%)	3/4 (75%)		2/4 (50%)	4/4 (100%)	2/4 (50%)	3/4 (75%)
Immunisation	2/4 (50%)	1/4 (25%)	1/4 (25%)	2/4 (50%)	3/5 (60%)	3/4 (75%)		0/4 (0%)	2/4 (50%)	1/4 (25%)	0/4 (0%)
PMTCT	2/4 (50%)	0/4 (0%)	1/4 (25%)	2/4 (50%)	3/5 (60%)	2/4 (50%)		1/4 (25%)	2/4 (50%)	2/4 (50%)	1/4 (25%)
Laboratory investigations	0/4 (0%)	0/4 (0%)	1/4 (25%)	1/4 (25%)	1/5 (20%)	1/4 (25%)		0/4 (0%)	1/4 (25%)	0/4 (0%)	0/4 (0%)
ANC outreaches in Posyandu											
No	3/4 (75%)	2/4 (50%)	2/4 (50%)	2/4 (50%)	2/6 (33%)	1/4 (25%)		3/4 (75%)	2/4 (50%)	3/4 (75%)	2/4 (50%)
Yes	1/4 (25%)	2/4 (50%)	2/4 (50%)	2/4 (50%)	4/6 (67%)	3/4 (75%)		1/4 (25%)	2/4 (50%)	1/4 (25%)	2/4 (50%)
Training											
Attended any training workshops on malaria in pregnancy	4/4 (100%)	3/4 (75%)	3/4 (75%)	4/4 (100%)	6/6 (100%)	3/4 (75%)		4/4 (100%)	2/4 (50%)	3/4 (75%)	4/4 (100%)
Had any on-the-job training from colleagues on malaria in pregnancy	2/4 (50%)	2/4 (50%)	1/4 (25%)	2/4 (50%)	2/6 (33%)	2/4 (50%)		0/4 (0%)	2/4 (50%)	2/4 (50%)	2/4 (50%)
Attended any training on malaria case management in pregnancy	4/4 (100%)	3/4 (75%)	3/4 (75%)	2/4 (50%)	4/6 (67%)	0/4 (0%)		3/4 (75%)	3/4 (75%)	2/4 (50%)	3/4 (75%)
Had any on-the-job training from colleagues on malaria case management	2/4 (50%)	1/4 (25%)	3/4 (75%)	3/4 (75%)	5/6 (83%)	1/4 (25%)		2/4 (50%)	4/4 (100%)	2/4 (50%)	2/4 (50%)
Attend the DHO training workshops on IPTp-DP	3/4 (75%)	1/4 (25%)	1/4 (25%)	2/4 (50%)	4/6 (67%)	2/4 (50%)		2/4 (50%)	1/4 (25%)	2/4 (50%)	4/4 (100%)
Had any on-the-job training from colleagues on IPTp-DP	4/4 (100%)	2/4 (50%)	3/4 (75%)	4/4 (100%)	6/6 (100%)	2/4 (50%)		2/4 (50%)	4/4 (100%)	4/4 (100%)	1/4 (25%)
Ever read guidelines on:											
Malaria in pregnancy	4/4 (100%)	4/4 (100%)	4/4 (100%)	4/4 (100%)	6/6 (100%)	3/4 (75%)		4/4 (100%)	4/4 (100%)	4/4 (100%)	4/4 (100%)
IPTp-DP drug administration & dosing	4/4 (100%)	4/4 (100%)	4/4 (100%)	4/4 (100%)	6/6 (100%)	4/4 (100%)		4/4 (100%)	4/4 (100%)	4/4 (100%)	4/4 (100%)
IPTp-DP side effects & management	4/4 (100%)	4/4 (100%)	4/4 (100%)	4/4 (100%)	6/6 (100%)	4/4 (100%)		4/4 (100%)	4/4 (100%)	4/4 (100%)	4/4 (100%)
SST-DP drug administration & dosing	4/4 (100%)	4/4 (100%)	4/4 (100%)	4/4 (100%)	6/6 (100%)	2/4 (50%)		4/4 (100%)	4/4 (100%)	4/4 (100%)	4/4 (100%)

Study results of effectiveness and adherence of IPTp-DP

Characteristic				Semi urban					Urban		
	Facility 4	Facility 6	Facility 1	Facility 5	Facility 3	Facility 2		Facility 9	Facility 10	Facility 8	Facility 7
SST-DP side effects & management	4/4 (100%)	4/4 (100%)	4/4 (100%)	4/4 (100%)	6/6 (100%)	4/4 (100%)		4/4 (100%)	4/4 (100%)	4/4 (100%)	4/4 (100%)
Malaria case management	4/4 (100%)	4/4 (100%)	4/4 (100%)	4/4 (100%)	6/6 (100%)	2/4 (50%)		4/4 (100%)	4/4 (100%)	4/4 (100%)	4/4 (100%)
Ever read job aides on:											
Malaria in pregnancy	3/4 (75%)	4/4 (100%)	4/4 (100%)	4/4 (100%)	6/6 (100%)	4/4 (100%)		4/4 (100%)	4/4 (100%)	4/4 (100%)	4/4 (100%)
IPTp-DP drug administration & dosing	3/4 (75%)	4/4 (100%)	4/4 (100%)	4/4 (100%)	6/6 (100%)	4/4 (100%)		4/4 (100%)	4/4 (100%)	4/4 (100%)	4/4 (100%)
IPTp-DP side effects & management	3/4 (75%)	4/4 (100%)	4/4 (100%)	4/4 (100%)	6/6 (100%)	4/4 (100%)		4/4 (100%)	4/4 (100%)	4/4 (100%)	4/4 (100%)
SST-DP drug administration & dosing	3/4 (75%)	4/4 (100%)	4/4 (100%)	4/4 (100%)	6/6 (100%)	4/4 (100%)		4/4 (100%)	4/4 (100%)	4/4 (100%)	4/4 (100%)
SST-DP side effects & management	3/4 (75%)	4/4 (100%)	4/4 (100%)	4/4 (100%)	6/6 (100%)	4/4 (100%)		4/4 (100%)	4/4 (100%)	4/4 (100%)	4/4 (100%)
Malaria case management	3/4 (75%)	4/4 (100%)	4/4 (100%)	4/4 (100%)	6/6 (100%)	3/4 (75%)		4/4 (100%)	4/4 (100%)	4/4 (100%)	4/4 (100%)
Have daily access to guidelines on:											
Malaria in pregnancy	3/4 (75%)	4/4 (100%)	3/4 (75%)	3/4 (75%)	6/6 (100%)	4/4 (100%)		4/4 (100%)	3/4 (75%)	4/4 (100%)	4/4 (100%)
IPTp-DP drug administration & dosing	3/4 (75%)	4/4 (100%)	3/4 (75%)	3/4 (75%)	6/6 (100%)	4/4 (100%)		4/4 (100%)	3/4 (75%)	4/4 (100%)	4/4 (100%)
IPTp-DP side effects & management	3/4 (75%)	4/4 (100%)	3/4 (75%)	3/4 (75%)	6/6 (100%)	4/4 (100%)		4/4 (100%)	3/4 (75%)	4/4 (100%)	4/4 (100%)
SST-DP drug administration & dosing	3/4 (75%)	4/4 (100%)	3/4 (75%)	3/4 (75%)	6/6 (100%)	4/4 (100%)		4/4 (100%)	3/4 (75%)	4/4 (100%)	4/4 (100%)
SST-DP side effects & management	3/4 (75%)	4/4 (100%)	3/4 (75%)	3/4 (75%)	6/6 (100%)	4/4 (100%)		4/4 (100%)	3/4 (75%)	4/4 (100%)	4/4 (100%)
Malaria case management	3/4 (75%)	4/4 (100%)	3/4 (75%)	3/4 (75%)	6/6 (100%)	3/4 (75%)		4/4 (100%)	3/4 (75%)	4/4 (100%)	4/4 (100%)
Have daily access to job aides on:											
Malaria in pregnancy	3/4 (75%)	4/4 (100%)	3/4 (75%)	3/4 (75%)	6/6 (100%)	3/4 (75%)		4/4 (100%)	3/4 (75%)	4/4 (100%)	4/4 (100%)
IPTp-DP drug administration & dosing	3/4 (75%)	4/4 (100%)	3/4 (75%)	3/4 (75%)	6/6 (100%)	4/4 (100%)		4/4 (100%)	3/4 (75%)	4/4 (100%)	4/4 (100%)
IPTp-DP side effects & management	3/4 (75%)	4/4 (100%)	3/4 (75%)	3/4 (75%)	6/6 (100%)	4/4 (100%)		4/4 (100%)	3/4 (75%)	4/4 (100%)	4/4 (100%)
SST-DP drug administration & dosing	3/4 (75%)	4/4 (100%)	3/4 (75%)	3/4 (75%)	6/6 (100%)	4/4 (100%)		3/4 (75%)	3/4 (75%)	4/4 (100%)	4/4 (100%)

Study results of effectiveness and adherence of IPTp-DP

Characteristic				Semi urban					Urban		
	Facility 4	Facility 6	Facility 1	Facility 5	Facility 3	Facility 2		Facility 9	Facility 10	Facility 8	Facility 7
SST-DP side effects & management	3/4 (75%)	4/4 (100%)	3/4 (75%)	3/4 (75%)	6/6 (100%)	4/4 (100%)		4/4 (100%)	3/4 (75%)	4/4 (100%)	4/4 (100%)
Malaria case management	3/4 (75%)	4/4 (100%)	3/4 (75%)	3/4 (75%)	6/6 (100%)	3/4 (75%)		4/4 (100%)	3/4 (75%)	4/4 (100%)	4/4 (100%)

Table S3 Acceptability of IPTp-DP among pregnant women in exit interviews

**Characteristic**	**n/N (%)**
Perception of IPTp-DP program	
Very good	179/1,366 (13.1%)
Good	907/1,366 (66.4%)
Don't mind	224/1,366 (16.4%)
Not good	56/1,366 (4.1%)
Perception of taking DP	
Very good	167/1,366 (12.2%)
Good	917/1,366 (67.1%)
Don't mind	226/1,366 (16.5%)
Not good	56/1,366 (4.1%)
Motivation of completing IPTp-DP	
Health benefits for baby	648/1,366 (47.4%)
Health benefits for pregnant women	474/1,366 (34.7%)
About drugs and self-efficacy	67/1,366 (4.9%)
Health workers competence	139/1,366 (10.2%)
Don't know / no response	545/1,366 (39.9%)
Demotivation of completing IPTp-DP	
Harm me and baby	18/1,366 (1.3%)
Uncertainty about drugs and efficacy	170/1,366 (12.4%)
Health workers incompetence	5/1,366 (0.4%)
Don't know / no response	1,116/1,366 (81.7%)
Side effects taking IPTp-DP	
No	643/694 (92.7%)
Yes	51/694 (7.3%)
Side effects taking DP previously	
No	607/733 (82.8%)
Yes	126/733 (17.2%)
Type of side effects after taking DP	
Nausea	78/1,366 (5.7%)
Vomiting	73/1,366 (5.3%)
Dizziness	55/1,366 (4.0%)
Other	34/1,366 (2.5%)
The main reason of side effects	
The medicine was too strong	88/148 (59.5%)
I was already feeling unwell	11/148 (7.4%)
Hadn't eaten before taking the medicine	9/148 (6.1%)
It was because the drug was fighting malaria	4/148 (2.7%)
Other	8/148 (5.4%)
Don't know	28/148 (18.9%)

Study results of effectiveness and adherence of IPTp-DP

**Characteristic**	**n/N (%)**
Have any problems finishing the medicine	
No	1,299/1,366 (95.1%)
Yes	67/1,366 (4.9%)
The main problem for you in finishing taking the medicine*	
Don't like the medicine	25/67 (37%)
Don't like one of the medicines	1/67 (1%)
Difficult to remember to take it	11/67 (16%)
Got another illness	2/67 (3%)
Made me feel sick	26/67 (39%)
Other	2/67 (3%)

*pregnant women who had problems finishing the medicine

Table S4 Predictors of delivery effectiveness comparing full vs partial/ non-effective delivery

Variables	n/N (%)	Univariable analysis			Multivariable analysis[1]	
		cOR (95%CI)	p-value		aOR (95%CI)	p-value
Age category						
15-19	42/101 (41.6%)	1.85 (1.21-2.82)	0.004		1.47 (0.66-3.26)	0.341
20-34	295/1,061 (27.8%)	Reference			Reference	
≥35	65/204 (31.9%)	1.21 (1.01-1.46)	0.038		1.26 (1.04-1.51)	0.017
Highest education completed						
No education/ Primary	68/151 (45.0%)	2.91 (1.23-6.87)	0.015		2.01 (1.08-3.75)	0.028
Middle / High school	247/872 (28.3%)	1.4 (0.97-2.03)	0.075		1.30 (0.99-1.72)	0.063
Diploma / University	64/291 (22.0%)	Reference			Reference	
Socioeconomic status						
Above poverty line	74/273 (27.1%)	Reference				
Below poverty line	328/1,093 (30.0%)	1.15 (0.83-1.6)	0.394			
Ethnic						
Non-Papua	260/1,007 (25.8%)	Reference			Reference	
Papua	142/359 (39.6%)	1.88 (0.74-4.75)	0.181		1.21 (0.72-2.02)	0.467
Marital status						
Single/ divorced / widow	43/86 (50.0%)	2.57 (1.26-5.24)	0.01		1.69 (0.73-3.91)	0.224
Married	359/1,280 (28.0%)	Reference			Reference	
Religion						
Catholic / Protestant	274/860 (31.9%)	1.37 (0.79-2.38)	0.256			
Islam / other	128/504 (25.4%)	Reference				

Study results of effectiveness and adherence of IPTp-DP

Variables	n/N (%)	Univariable analysis			Multivariable analysis[1]	
		cOR (95%CI)	p-value		aOR (95%CI)	p-value
Gestational Age						
Second Trimester	265/784 (33.8%)	1.66 (1.27-2.17)	<0.0001		3.13 (2.11-4.63)	<0.0001
Third Trimester	137/582 (23.5%)	Reference			Reference	
Gravidity						
Primigravidae	128/414 (30.9%)	1.11 (0.86-1.43)	0.431			
Multigravidae	274/952 (28.8%)	Reference				
Number of ANC visit						
≤ 3 times	274/910 (30.1%)	1.1 (0.67-1.81)	0.694			
> 3 times	128/456 (28.1%)	Reference				
Has an illness at ANC						
No	389/1,329 (29.3%)	Reference				
Yes	13/37 (35.1%)	1.31 (0.52-3.32)	0.571			
Previous malaria test within past 28 days						
No	263/801 (32.8%)	1.5 (0.83-2.71)	0.182		1.37 (0.76-2.46)	0.289
Yes	139/565 (24.6%)	Reference			Reference	
Had a previous course of IPTp-DP						
No	248/1,041 (23.8%)	Reference			Reference	
Yes	154/325 (47.4%)	2.88 (2.15-3.86)	<0.0001		4.30 (3.07-6.01)	<0.0001
Health insurance ownership						
No	144/395 (36.5%)	1.59 (1.16-2.17)	0.004		1.33 (1.09-1.63)	0.0044

Study results of effectiveness and adherence of IPTp-DP

Variables	n/N (%)	Univariable analysis			Multivariable analysis[1]	
		cOR (95%CI)	p-value		aOR (95%CI)	p-value
Yes	258/971 (26.6%)	Reference			Reference	
Side effects taking DP previously						
No	253/607 (41.7%)	1.05 (0.5-2.22)	0.896			
Yes	51/126 (40.5%)	Reference				
Location						
Urban	221/975 (22.7%)	Reference			Reference	
Semi urban[2]	181/391 (46.3%)	2.94 (0.7-12.35)	0.141		2.44 (0.72-8.26)	0.151

[1]n= 1,314

[3]Semi-urban health facilities are 1, 2, 3, 4, 5, 6

Table S5 Predictors of delivery effectiveness comparing full/partial vs non-effective delivery (post hoc)

Variables	n/N (%)	Univariable analysis			Multivariable analysis[1]	
		cOR (95%CI)	p-value		aOR (95%CI)	p-value
Age category						
15-19	54/101 (53.5%)	1.79 (1.14-2.8)	0.011		1.67 (0.99-2.8)	0.053
20-34	415/1,061 (39.1%)	Reference			Reference	
≥35	87/204 (42.6%)	1.16 (0.98-1.37)	0.084		1.07 (0.88-1.3)	0.514
Highest education completed						
No education/ Primary	82/151 (54.3%)	2.27 (1.05-4.92)	0.038		1.86 (0.72-4.79)	0.198
Middle / High school	350/872 (40.1%)	1.28 (0.93-1.76)	0.127		1.15 (0.88-1.5)	0.299
Diploma / University	100/291 (34.4%)	Reference			Reference	
Socioeconomic status						
Above poverty line	110/273 (40.3%)	Reference				
Below poverty line	446/1,093 (40.8%)	1.02 (0.75-1.39)	0.893			
Ethnic						
non-Papua	384/1,007 (38.1%)	Reference				
Papua	172/359 (47.9%)	1.49 (0.69-3.21)	0.307			
Marital status						
Single/ divorced / widow	49/86 (57.0%)	2.02 (1.05-3.89)	0.035		1.69 (0.94-3.05)	0.079
Married	507/1,280 (39.6%)	Reference			Reference	
Religion						
Catholic / Protestant	356/860 (41.4%)	1.07 (0.72-1.61)	0.73			
Islam / other	200/504 (39.7%)	Reference				

Study results of effectiveness and adherence of IPTp-DP

Variables	n/N (%)	Univariable analysis			Multivariable analysis[1]	
		cOR (95%CI)	p-value		aOR (95%CI)	p-value
Gestational Age						
Second Trimester	336/784 (42.9%)	1.23 (0.9-1.68)	0.184		2.13 (1.31-3.47)	0.0025
Third Trimester	220/582 (37.8%)	Reference			Reference	
Gravidity						
Primigravidae	175/414 (42.3%)	1.1 (0.85-1.42)	0.476			
Multigravidae	381/952 (40.0%)	Reference				
Number of ANC visit						
≤ 3 times	371/910 (40.8%)	1.01 (0.69-1.47)	0.966			
> 3 times	185/456 (40.6%)	Reference				
Has an illness at ANC						
No	540/1,329 (40.6%)	Reference				
Yes	16/37 (43.2%)	1.11 (0.51-2.44)	0.788			
Previous malaria test within past 28 days						
No	354/801 (44.2%)	1.42 (0.93-2.18)	0.105		1.13 (0.67-1.91)	0.655
Yes	202/565 (35.8%)	Reference			Reference	
Had a previous course of IPTp-DP						
No	346/1,041 (33.2%)	Reference			Reference	
Yes	210/325 (64.6%)	3.67 (2.39-5.64)	<0.0001		4.99 (2.97-8.4)	<0.0001
Health insurance ownership						
No	198/395 (50.1%)	1.72 (1.28-2.32)	<0.0001		1.62 (1.14-2.31)	0.0077

Study results of effectiveness and adherence of IPTp-DP

Variables	n/N (%)	Univariable analysis			Multivariable analysis[1]	
		cOR (95%CI)	p-value		aOR (95%CI)	p-value
Yes	358/971 (36.9%)	Reference			Reference	
Side effects taking DP previously						
No	328/607 (54.0%)	1.21 (0.59-2.5)	0.6			
Yes	62/126 (49.2%)	Reference				
Location						
Urban	357/975 (36.6%)	Reference				
Semi urban[2]	199/391 (50.9%)	1.79 (0.59-5.45)	0.302			

[1]n= 1,314

[2]Semi-urban health facilities are 1, 2, 3, 4, 5, 6

Table S6 Predictors of adherence comparing full vs partial/ non-adherence

Variables	n/N (%)	Univariable analysis			Multivariable analysis[1]	
		cOR (95%CI)	p-value		aOR (95%CI)	p-value
Age category						
15-19	42/49 (85.7%)	Reference				
20-34	319/354 (90.1%)	1.52 (0.63-3.66)	0.351			
≥35	76/81 (93.8%)	2.53 (0.43-14.86)	0.303			
Highest education completed						
No education/ Primary	66/72 (91.7%)	1.29 (0.54-3.09)	0.568			
Middle / High school	273/305 (89.5%)	Reference				
Diploma / University	77/85 (90.6%)	1.13 (0.46-2.75)	0.791			
Socioeconomic status						
Above poverty line	85/96 (88.5%)	Reference				
Below poverty line	352/388 (90.7%)	1.27 (0.83-1.93)	0.277			
Ethnic						
non-Papua	303/332 (91.3%)	Reference				
Papua	134/152 (88.2%)	1.49 (0.69-3.21)	0.307			
Marital status						
Single/ divorced / widow	38/48 (79.2%)	Reference			Reference	
Married	399/436 (91.5%)	2.84 (1.24-6.51)	0.014		3.50 (1.55-7.89)	0.0028
Religion						
Catholic / Protestant	277/310 (89.4%)	Reference				
Islam / other	160/174 (92.0%)	1.36 (0.85-2.19)	0.203			

Study results of effectiveness and adherence of IPTp-DP

Variables	n/N (%)	Univariable analysis			Multivariable analysis[1]	
		cOR (95%CI)	p-value		aOR (95%CI)	p-value
Gestational Age						
Second Trimester	280/302 (92.7%)	2.03 (0.99-4.15)	0.054		1.92 (0.88-4.21)	0.102
Third Trimester	157/182 (86.3%)	Reference			Reference	
Gravidity[2]						
Primigravidae	131/148 (88.5%)	Reference				
Multigravidae	306/336 (91.1%)	1.32 (0.87-2.03)	0.196			
Acceptance of IPTp-DP						
Not good or don't mind	13/16 (81.2%)	Reference				
Good or very good	424/468 (90.6%)	2.22 (0.6-8.19)	0.23			
Side effects taking DP previously						
No	260/285 (91.2%)	1.04 (0.34-3.19)	0.945			
Yes	50/55 (90.9%)	Reference				
Number of ANC visit						
≤ 3 times	285/319 (89.3%)	Reference			Reference	
> 3 times	152/165 (92.1%)	1.39 (0.95-2.05)	0.092		1.95 (1.22-3.13)	0.0054
First ANC visit						
No	346/384 (90.1%)	Reference				
Yes	91/100 (91.0%)	1.11 (0.65-1.9)	0.701			
Has an illness at ANC						
No	429/474 (90.5%)	2.38 (0.28-20.25)	0.426			

Study results of effectiveness and adherence of IPTp-DP

Variables	n/N (%)	Univariable analysis			Multivariable analysis[1]	
		cOR (95%CI)	p-value		aOR (95%CI)	p-value
Yes	8/10 (80.0%)	Reference				
Previous malaria test within past 28 days						
No	267/299 (89.3%)	Reference				
Yes	170/185 (91.9%)	1.36 (0.78-2.36)	0.278			
Had a previous course of IPTp-DP						
No	280/311 (90.0%)	Reference				
Yes	157/173 (90.8%)	1.09 (0.74-1.6)	0.673			
Health insurance ownership						
No	151/167 (90.4%)	1.02 (0.5-2.08)	0.95			
Yes	286/317 (90.2%)	Reference				
Location						
Urban	271/300 (90.3%)	1.01 (0.43-2.4)	0.976			
Semi urban[3]	166/184 (90.2%)	Reference				
Effective delivery						
Partial effective delivery	98/119 (82.4%)	Reference			Reference	
Full effective delivery	339/365 (92.9%)	2.79 (1.47-5.3)	0.002		3.18 (1.82-5.54)	<0.0001

[1]n= 484

[2]The variable 'gravidity' was excluded from the multivariable analysis based on the Akaike Information Criterion (AIC), as the model including gravidity had an AIC of 295.44, whereas the model without it had a lower AIC of 293.80, indicating better model fit.

[3]Semi-urban health facilities are 1, 2, 3, 4, 5, 6

Study results of effectiveness and adherence of IPTp-DP

Table S7 Factors influencing effectiveness of IPTp-DP delivery by DOT and adherence

Quantitative finding	Qualitative theme	Explanatory sub-themes
**IPTp-DP delivery**		
Delivery effectiveness of IPTp-DP: 41% (source: exit interview)	Service delivery: IPTp-DP delivery	• Women in one community reported not being offered IPTp-DP following community-wide rejection of the programme<br>• Not offered IPTp-DP by healthcare provider<br>• Food is provided to women prior to taking DP to reduce potential side effects
	Product and technology: Supply chain	• Stock outs of DP meant women were not offered IPTp-DP during ANC visits at some time points<br>• Stock outs of DP damaged women's trust in the IPTp program
	Product and technology: Acceptability & demand for DP	• Women need support from their families, husbands to take IPTp DP<br>• Husbands can be a barrier to IPTp-DP uptake – they are hesitant about it (and they are the decision makers)<br>• Women's reluctance to take IPTp-DP was fuelled by dislike of side effects and safety fears<br>• Women who previously experienced malaria in pregnancy valued IPTp-DP
	Human resources: staffing considerations	• Additional time requirements needed to give women information about IPTp<br>• Would be helpful to have dedicated IPTp staff
	Human resources: training	• Lack of interpersonal and counselling skills among some healthcare providers impeded their ability to successfully convince women to take up IPTp-DP<br>• Endline data suggests this improved following provider training on effective communication
IPTp-DP 1,2,3+coverage: 43%, 25%, 23% (source: routine data, see appendix 1, Table S2, pp 50)	Service delivery: IPTp delivery challenges	• Women often start ANC late, give birth before being able to take up multiple IPTp-DP courses<br>• Population movement means women don't consistently come to ANC to receive IPTp

	Service delivery: Factors influencing adherence to monthly IPTp regimen	• Some women who experience side effects do not want to continue with the next course of IPTp-DP – often because their husbands do not want them to<br>• On-going education can change women's minds about discontinuing with IPTp-DP
**Adherence**		
Full adherence: 90.3% (source: home visit interview).	Service delivery: factors influencing adherence to dose 2,3 (healthcare provider perspective)	• Women completed the doses because they understand the importance of IPTp-DP<br>• Family support has a positive influence on adherence to IPTp-DP
	Service delivery: factors influencing adherence to dose 2,3 (pregnant women perspective)	• Pregnant women have a personal motivation to protect themselves and their babies from malaria.<br>• Explanations from midwife – about importance of IPTp and how best to take it (e.g., to minimise side effects)<br>• Having a strategy for taking DP (to minimise negative effects)
	Service delivery: Follow up to check on adherence	• Women complete doses 2, 3 at home because healthcare providers check adherence to dose-2, dose-3 by either follow up contact via home visits when possible, phone/video calls, women send photo evidence.<br>• In situations where the woman does have access to a mobile phone, a CHW conducts a follow up-contact via home visit
	Service delivery: Challenges with follow up	• Issues when contacting women for follow up (e.g., phone number doesn't work, they are away at work, need data to make video calls)<br>• Women are not at home when the healthcare provider arrives for the follow up
Married ↑	Service delivery: factors influencing adherence to dose 2,3 (healthcare provider perspective)	• Family support, particularly from husbands, positively influenced women's adherence to IPTp-DP as they are reminded and encouraged to complete all doses.
Full delivery effectiveness ↑	Service delivery: IPTp-DP delivery	• Dose-1 taken at facility by DOT enhances adherence by allowing healthcare providers to monitor and manage side effects immediately, which encourages women to continue with subsequent doses.

↑ indicates that the category has a higher odds ratio compared to the reference category

Figures S3-13 Health facility performance data showing IPTp-DP coverage and CQI data

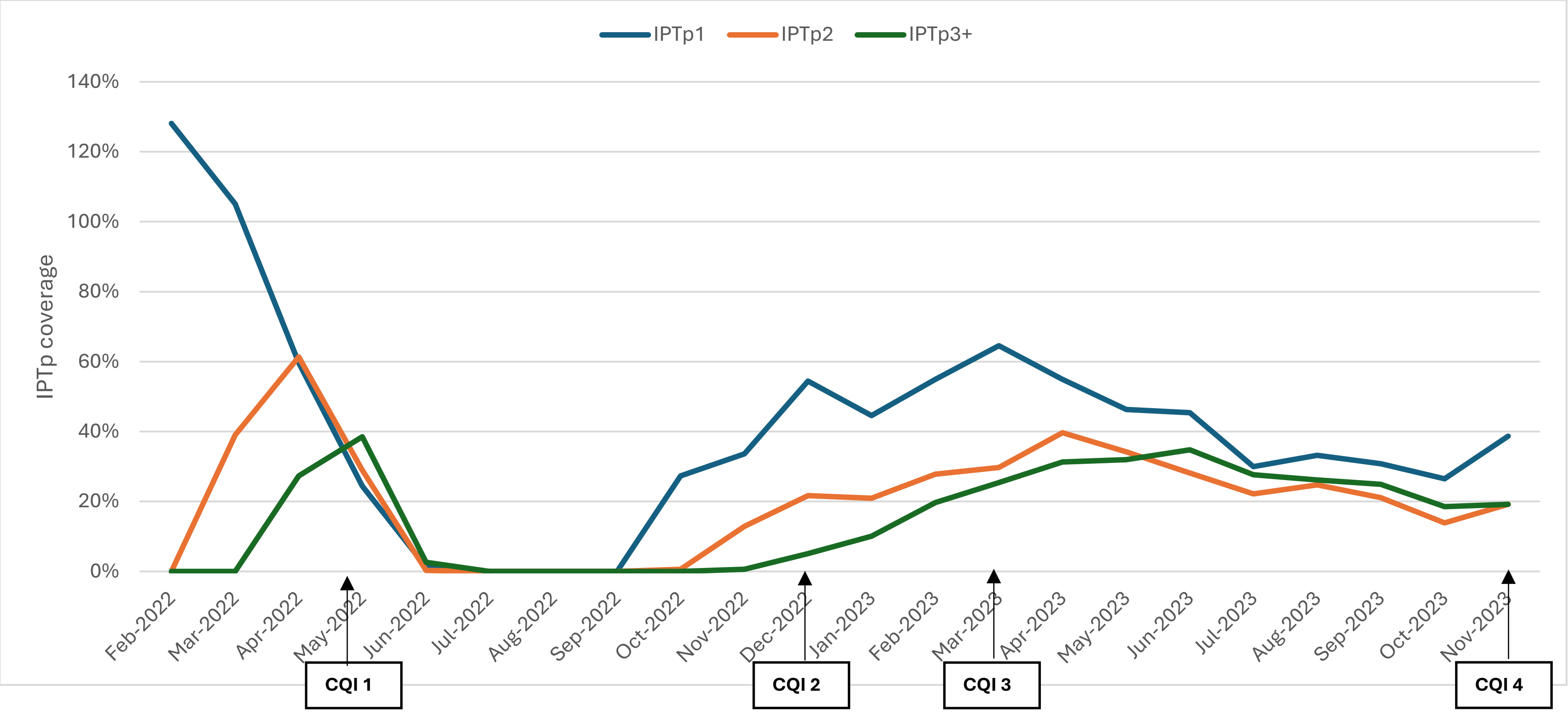

Figure S3 All health facilities IPTp-DP coverage

Figure S3 shows IPTp-1 coverage increased from 34% to 54% following the CQI cycle in December 2022, from 55% to 65% after the March 2023 cycle, and from 26% to 39% after the November 2023 cycle. Figures S4–S12 present not only IPTp-DP coverage trends but also summaries of the key findings from the CQI workshops. The CQI workshops utilised various quality improvement tools such as the Fishbone Diagram to categorise problems, and the 5 Whys technique to identify root causes of delivery challenges and design actionable improvements. The health facilities were responsible for identifying and reporting on their specific delivery challenges during the workshops, while the DHO facilitated the sessions. Unlike iterative CQI models, the DHO approach was to use each workshop for unique issues and solutions, not necessarily linked to previous workshops.

Health facility performance of IPTp-DP coverage and CQI data

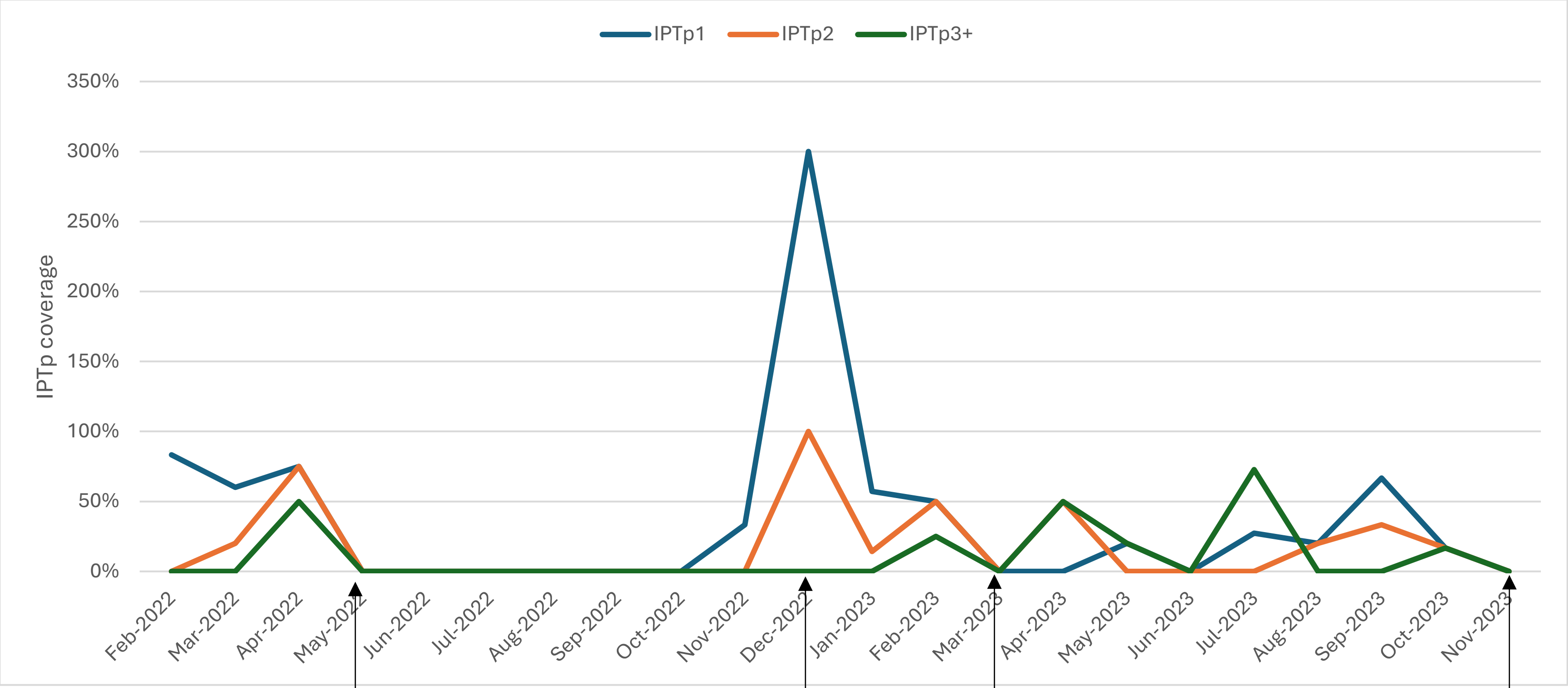

Figure S4 Health facility 1 IPTp-DP coverage and CQI notes

CQI 1
Monitor: IPTp-DP coverage monitoring initiated both at main health facility and at integrated health posts
Improve: Establishment of facility IPTp-DP team, development of SOPs, and provision of education campaign for community health workers and pregnant women at Integrated health post and MCH classes.
Define: Limited staff capacity and occasional stockouts of DP.
Plan: No specific solution plan

CQI 2
Monitor: Restarted IPTp-DP coverage monitoring in November 2022.
Improve: Structured service flows for eligible IPTp-DP pregnant women.
Define: Limited availability of DP and scheduling conflicts due to malaria-positive cases.
Plan: Empowered malaria community health workers and distributed educational materials

CQI 3
Monitor: Enhanced monitoring through home visits and community involvement in reminding and supporting pregnant women to complete their medication.
Improve: Completion of SOPs development, high achievement in DOT for the first dose
Define: Decline in subsequent visits for IPTp-DP 2 by pregnant women due to high community mobility.
Plan: Involved family members in monitoring and introduced rewards for completion.

CQI 4
Monitor: Moderate success in achieving IPTp-DP targets with significant efforts in monitoring and educating pregnant women.
Improve: Continued home visits and engaged community leaders to address refusal.
Define: Decline in attendance for IPTp-DP follow-up doses due to low motivation and lack of knowledge among pregnant women.
Plan: Improved education efforts and proposed rewards for completing IPTp-DP doses

Health facility performance of IPTp-DP coverage and CQI data

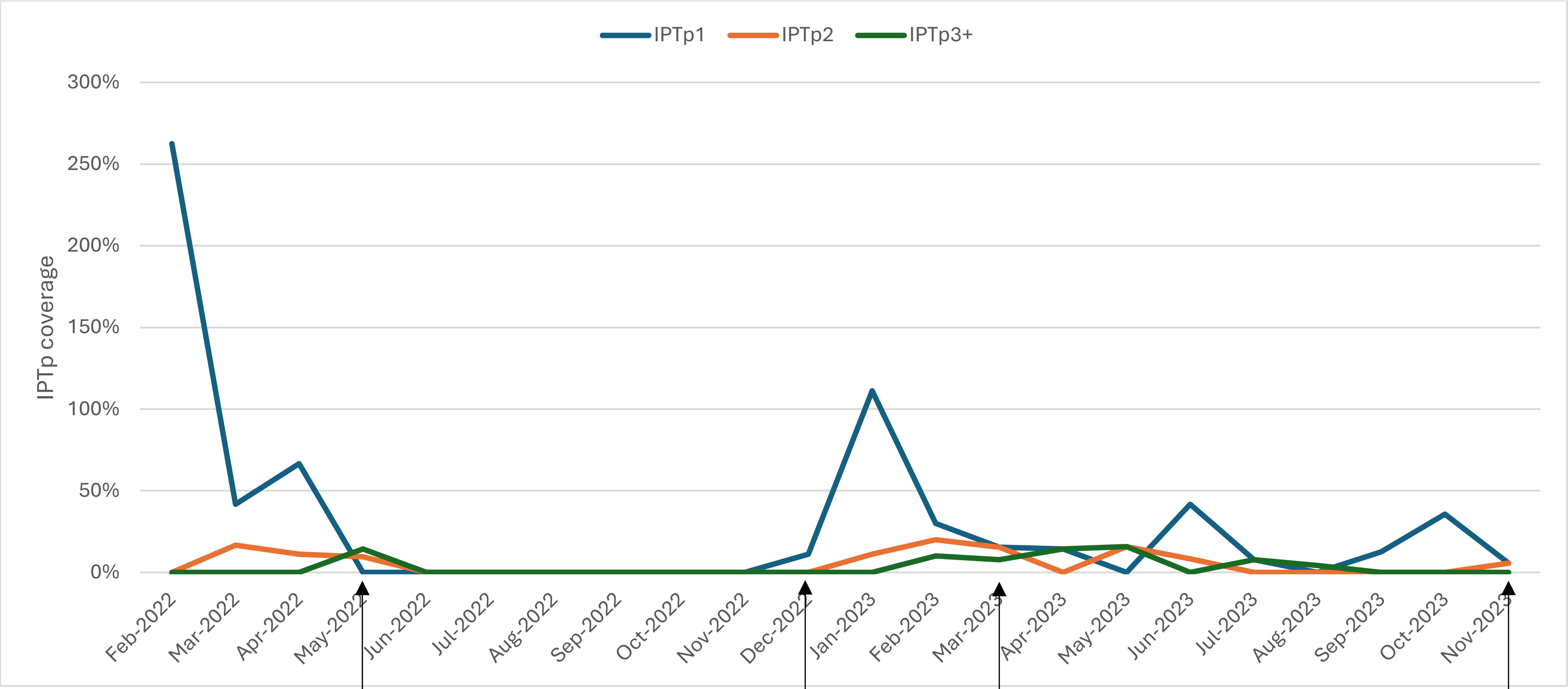

Figure S5 Health facility 2 IPTp-DP coverage and CQI notes

CQI 1
Monitor: Monitoring IPTp-DP coverage of IPTp-DP services initiated at the main health facility and one of integrated health post.
Improve: Completion of administrative and logistics setup, including decree issuance, SOP development, and initiation of IPTp-DP service.
Define: Some patients began refusing IPTp-DP services after initial success.
Plan: Education campaign to other health workers

CQI 2
Monitor: Resumed monitoring IPTp-DP that restarted in December 2022.
Improve: Involvement of local midwives, and intensified education.
Define: Programme halted due to material shortages and community reluctance, particularly at specific integrated health posts.
Plan: Enhance community engagement, secure funding, and optimize the use of trusted local midwives.

CQI 3
Monitor: High achievement in DOT for the first dose
Improve: Continued administration of doses at home, with active follow-up by health workers and community health workers.
Define: Low participation due to lack of support from families and inadequate resources.
Plan: Increase community awareness through banners, improve resource availability, and involve community leaders and families.

CQI 4
No data available because no representative attended the CQI workshop

Health facility performance of IPTp-DP coverage and CQI data

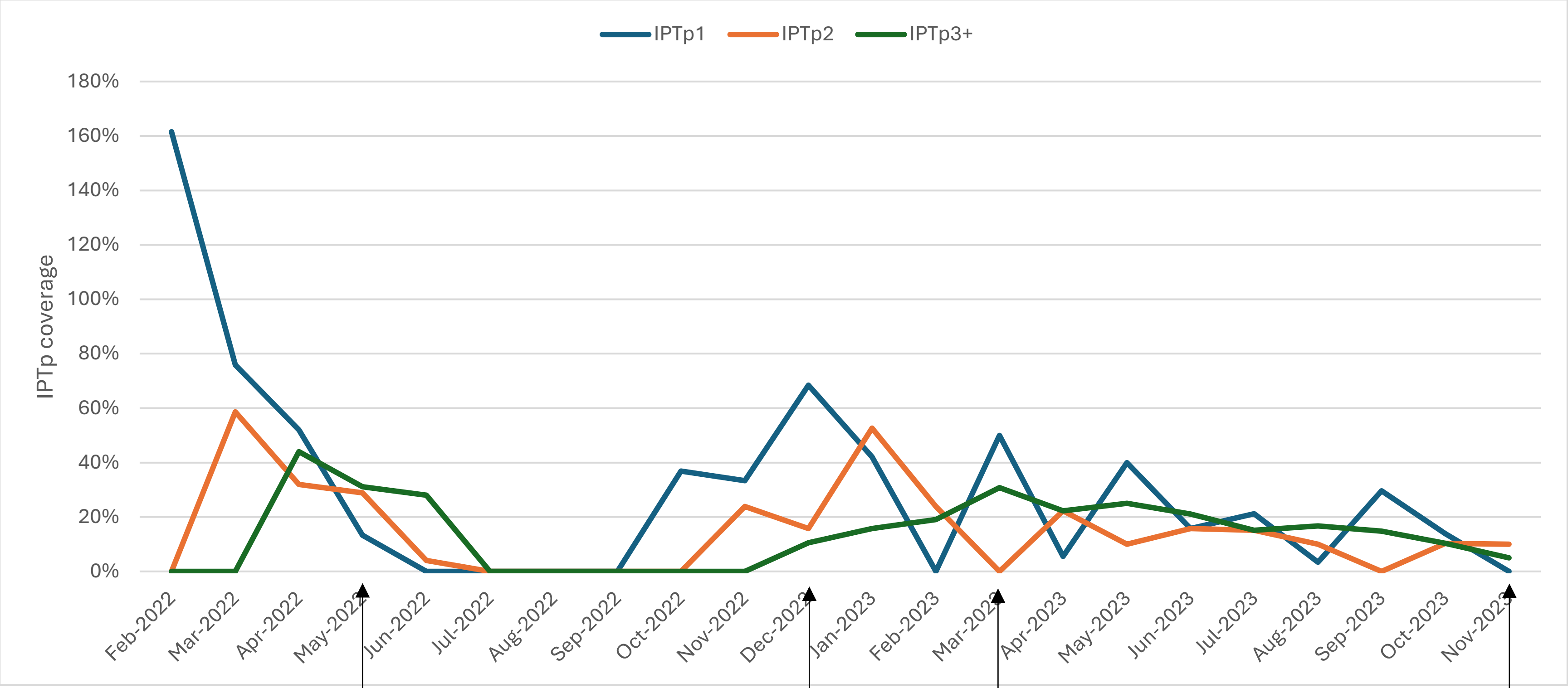

Figure S6 Health facility 3 IPTp-DP coverage and CQI notes

CQI 1
Monitor: Decreased participation in IPTp-DP that started across healthcare facilities.
Improve: Completion of administrative setup, including the issuance of the IPTp-DP decree and SOPs; initiation of the IPTp-DP programme in February 2022.
Define: fear of drug side effects and lack of support from husbands.
Plan: Implement home visits and encourage ANC visits with partners to increase participation.

CQI 2
Monitor: Decreased IPTp-DP participation
Improve: Continued counselling and administration of the first dose at health centres, with monitoring of subsequent doses at home
Define: fear of side effects and lack of support from husbands.
Plan: Face-to-face counselling sessions with pregnant women and their husbands, along with home visits to reinforce the importance of the programme.

CQI 3
Monitor: Ongoing IPTp-DP activities with updated monitoring and implementation indicators.
Improve: Intensive counselling and continuous monitoring through home visits and health worker reminders.
Define: Low coverage of IPTp-DP doses 2 to 6 due to trauma from side effects and lack of husband's agreement.
Plan: Conduct cross-sectoral education on IPTp-DP, create a WhatsApp group for better communication, and involve community leaders and cadres in the programme.

CQI 4
Monitor: Completion of IPTp-DP implementation with ongoing challenges in achieving target of IPTp coverage.
Improve: Reinforce education and campaign efforts through both direct and indirect methods, including the use of role models and social media.
Define: Persistent issues with fear of medication side effects and lack of community awareness.
Plan: Increase promotion and education efforts, involve community leaders and family members, and ensure consistent communication and support through

Health facility performance of IPTp-DP coverage and CQI data

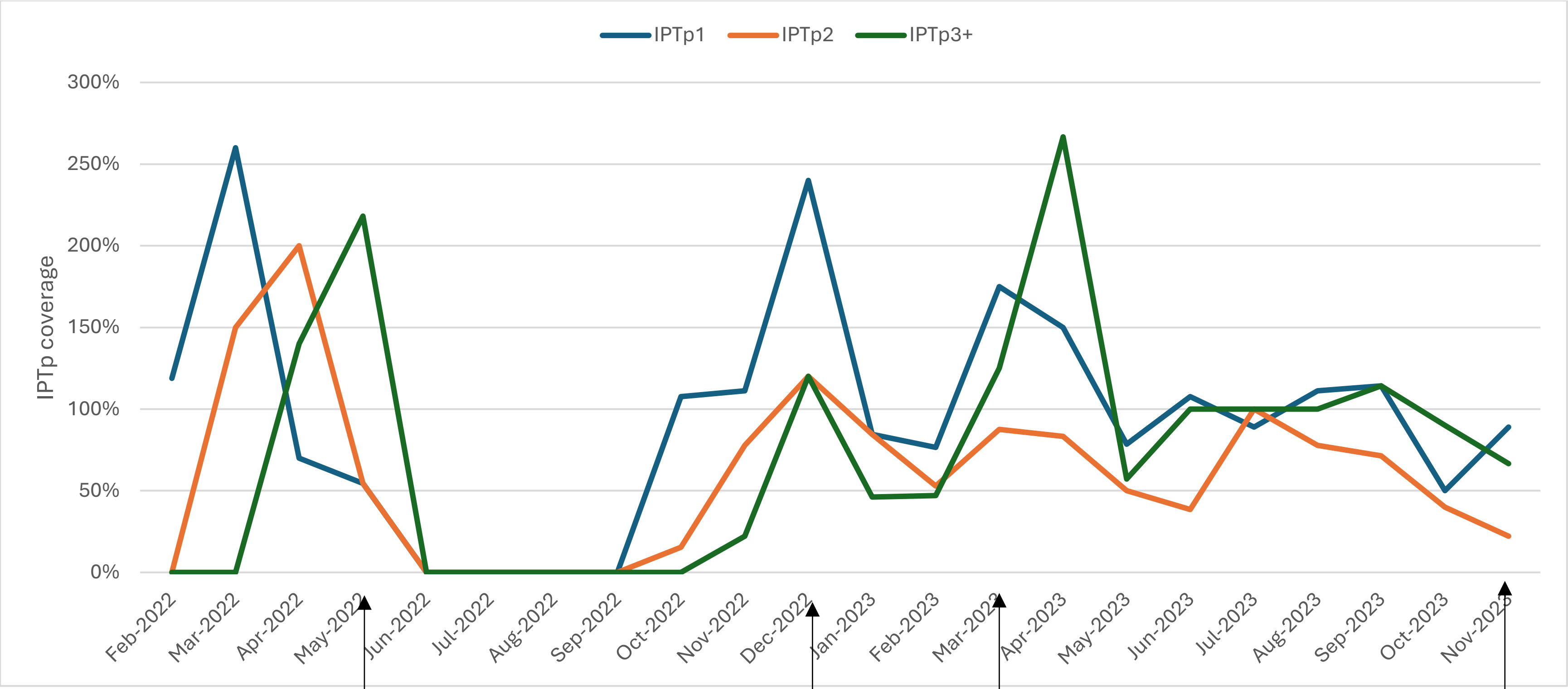

Figure S7 Health facility 4 IPTp-DP coverage and CQI notes

CQI 1
Monitor: Monitoring the coverage of IPTp-DP initiation, with personalized patient counseling, particularly for Indigenous Papuans.
Improve: Completion of administrative and logistics setup, including the issuance of the IPTp-DP decree, SOPs, and initiation of IPTp-DP services at the Health Centre.
Define: No significant challenges were perceived at this stage.
Plan: Continue the program with the current strategies, including personalized education and door-to-door monitoring for side effects.

CQI 2
Monitor: High achievement rates for IPTp-DP targets, with 100% compliance with process indicators such as SK and SOPs.
Improve: Continued personalized services, including door-to-door visits, and administration of IPTp-DP doses directly at the Health Centre.
Define: Decreased IPTp-DP achievement compared to earlier stages, issues with acceptance of education, and lack of family support, particularly from husbands.
Plan: Address the decline by standardizing screening processes, enhancing family engagement, and improving education strategies to be more culturally appropriate.

CQI 3
Monitor: Continued high achievement in initial IPTp-DP doses, but a noted decrease in subsequent doses (IPTp 3 and beyond).
Improve: Focused efforts on early pregnancy detection and personalized education, with continued home visits for medication administration.
Define: Decreasing coverage for IPTp-DP 3 due to lack of early ANC visit and high population mobility.
Plan: Coordination between village midwives to improve early ANC detection and ensure timely IPTp-DP administration.

CQI 4
Monitor: Surpassing targets for IPTp-DP administration, especially for the first dose, with significant success in early stages of the program.
Improve: Implemented home visits and malaria screening during the first ANC visit, with coordination between village midwives and the health promotion team.
Define: Persistent issues with low attendance during the first trimester due to population mobility and socio-cultural factors.
Plan: Continue and enhance current strategies, focusing on early identification of pregnant women, community engagement, and education to improve ANC uptake and timely IPTp-DP administration.

Health facility performance of IPTp-DP coverage and CQI data

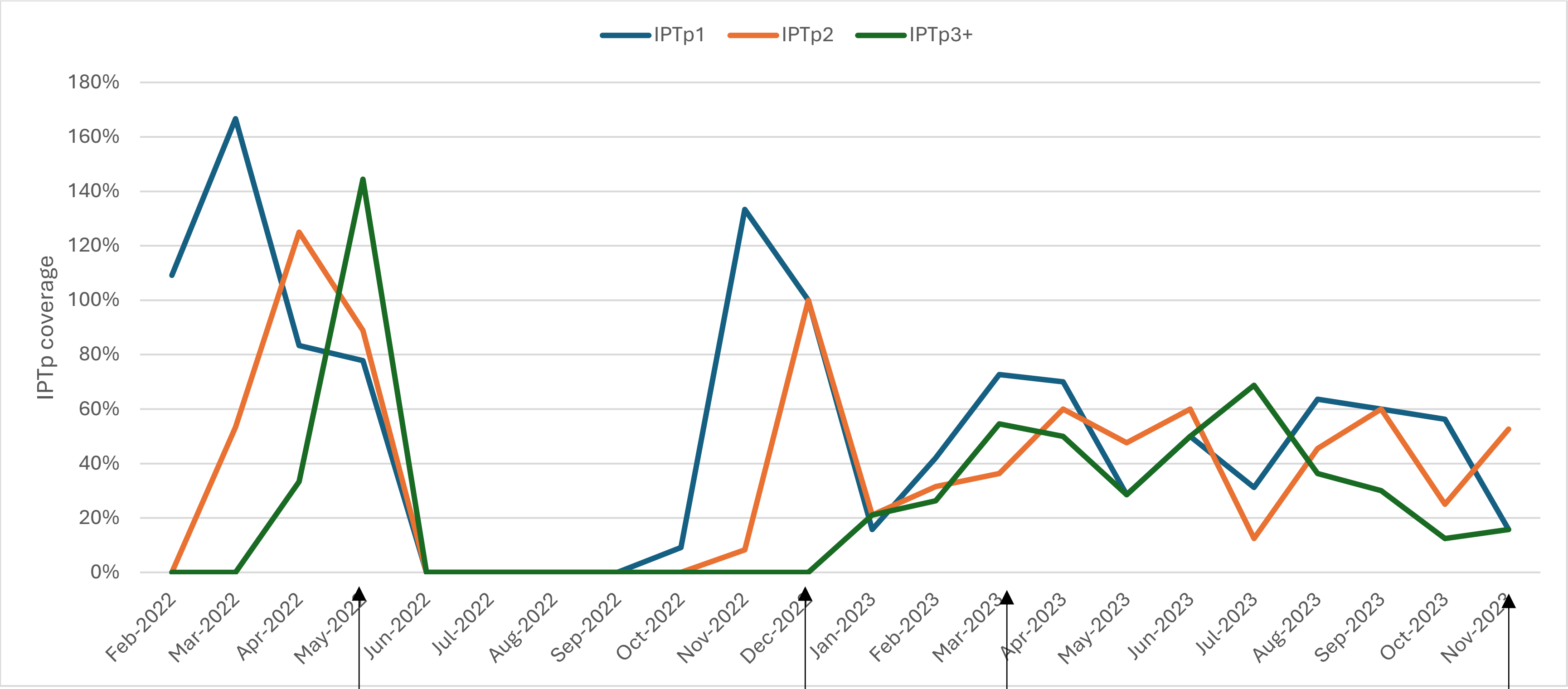

Figure S8 Health facility 5 IPTp-DP coverage and CQI notes

CQI 1
Monitor: IPTp-DP program implemented for 4 months, achieving target, with consistent monthly participation.
Improve: DOT via telephone, home visits, and providing medication for mothers with complaints. Conducted IPTp-DP counseling sessions at all integrated health posts.
Define: Shortage of MCH staff, no IPTp-DP community health workers, loss of contact with patients, side effects in patients, and transportation challenges.
Plan: Contact patients via telephone during home visits, ensure consistent follow-up, and manage complaints according to SOP.

CQI 2
Monitor: Performance indicators show some falling below targets while subsequent stages showed a decline.
Improve: Enhanced education and counseling, with a focus on addressing previous complaints from participants. Continued monitoring and follow-up activities, with attention to maintaining patient contact.
Define: Issues related to process, human factors, and environment, including lack of family support and low participation due to fear of side effects.
Plan: Prioritize addressing complaints from previous participants, increase knowledge among pregnant women, and strengthen family support.

CQI 3
Monitor: Achievement for IPTp-DP 3 and beyond remains low. IPTp-DP 1 and 2 show moderate success, with some improvement in patient adherence.
Improve: Conduct education and counseling in pregnant women's classes, share experiences from successful participants, and maintain consistent medication monitoring and follow-up.
Define: Pregnant women's nomadic living conditions, lack of mobile phones, and husbands' prohibition of participation are significant barriers.
Plan: Provide training in effective communication for all midwives, engage husbands and families in education sessions, and ensure continuous support for participating women

CQI 4
Monitor: Overall, IPTp-DP indicator achievements remain below targets, with significant drop-offs in IPTp-DP 3 and beyond.
Improve: Continue current outreach and education strategies, emphasizing the importance of IPTp-DP through both group and individual counseling. Ensure all midwives are trained in effective communication techniques.
Define: Persistent challenges include husbands' prohibition, myths, and late initiation of IPTp-DP in the second or third trimester.
Plan: Address root causes through targeted education, ongoing support for pregnant women, and continuous monitoring to ensure timely interventions

Health facility performance of IPTp-DP coverage and CQI data

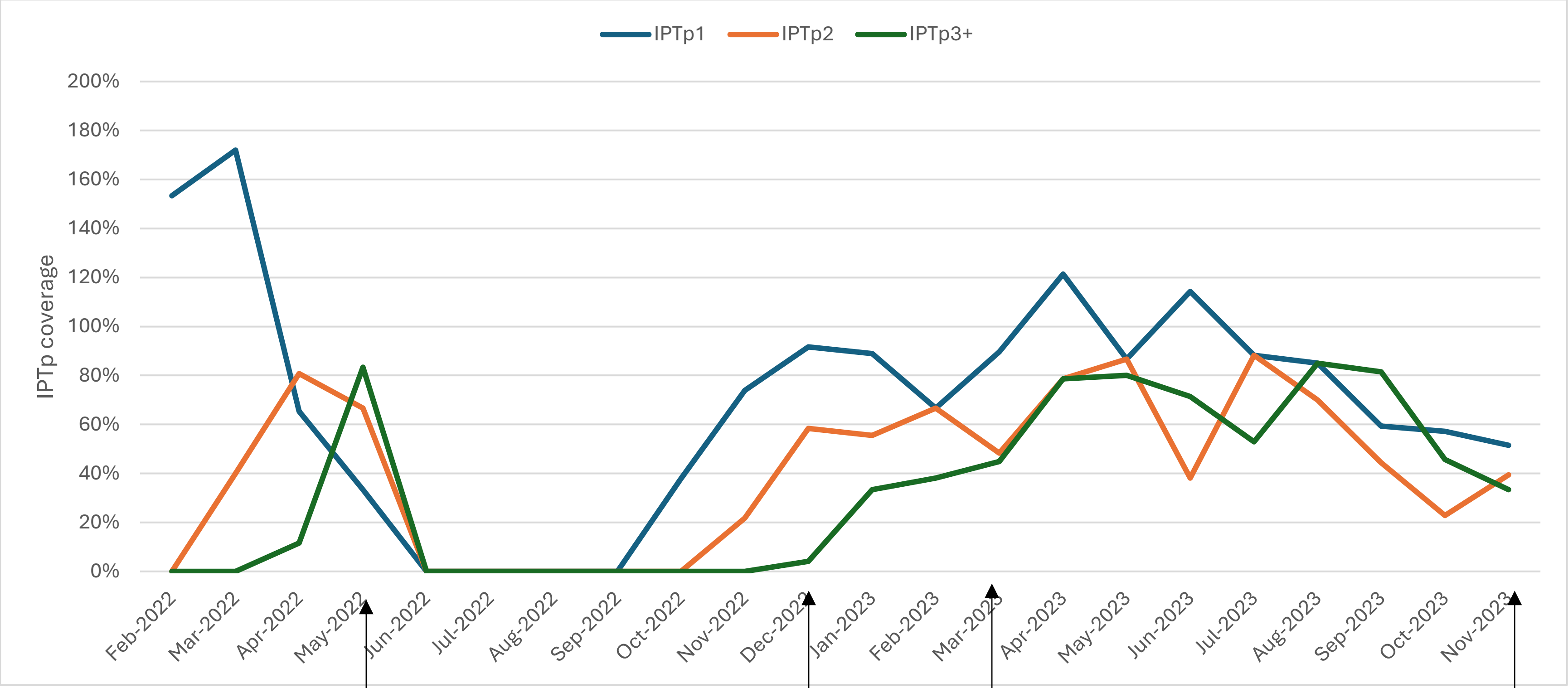

Figure S9 Health facility 6 IPTp-DP coverage and CQI notes

CQI 1
Monitor: Monitoring of inputs, including the SOPs developed and implemented and 83% of integrated health posts conducting IPTp-DP activities.
Improve: Home visits were conducted for mothers who did not attend ANC, and community health workers were assigned to invite pregnant women to the integrated health service posts.
Define: Some integrated health posts did not conduct IPTp-DP activities due to issues such as workforce reductions and low availability of anti-malarial medication.
Plan: Ensuring the stock of anti-malarial drugs and empowering community health workers to visit and inform pregnant women

CQI 2
Monitor: Monitoring the IPTp-DP that was restarted, and 100% participation of integrated health posts.
Improve: Ensuring the second and third doses were given to take home, with monitoring via phone calls.
Define: There was a 4-month stockout of anti-malarial medication, and some pregnant women were reluctant to join the programme.
Plan: Strengthening education and health promotion was prioritised, particularly by targeting family members and dispelling myths about anti-malarial medication.

CQI 3
Monitor Coverage for IPTp 1 increased, although participation significantly dropped in later doses.
Improve: Enhanced efforts in counselling and monitoring medication adherence.
Define: Difficulties were encountered in engaging the migrant population, particularly in Pomako Cendrawasih village, where there was strong resistance to the programme.
Plan: Strengthening education and health promotion strategies to address persistent cultural barriers and misconceptions.

CQI 4
Monitor: IPTp-DP 1 coverage reached target but subsequent doses show a decline.
Improve: Focus was on strengthening health promotion and communication strategies, especially targeting military families.
Define: Military wives, in particular, refused to participate due to misunderstandings and the absence of their husbands.
Plan: Targeted education campaigns, inclusive communication strategies, and empowerment efforts to reach women independently of their husbands.

Health facility performance of IPTp-DP coverage and CQI data

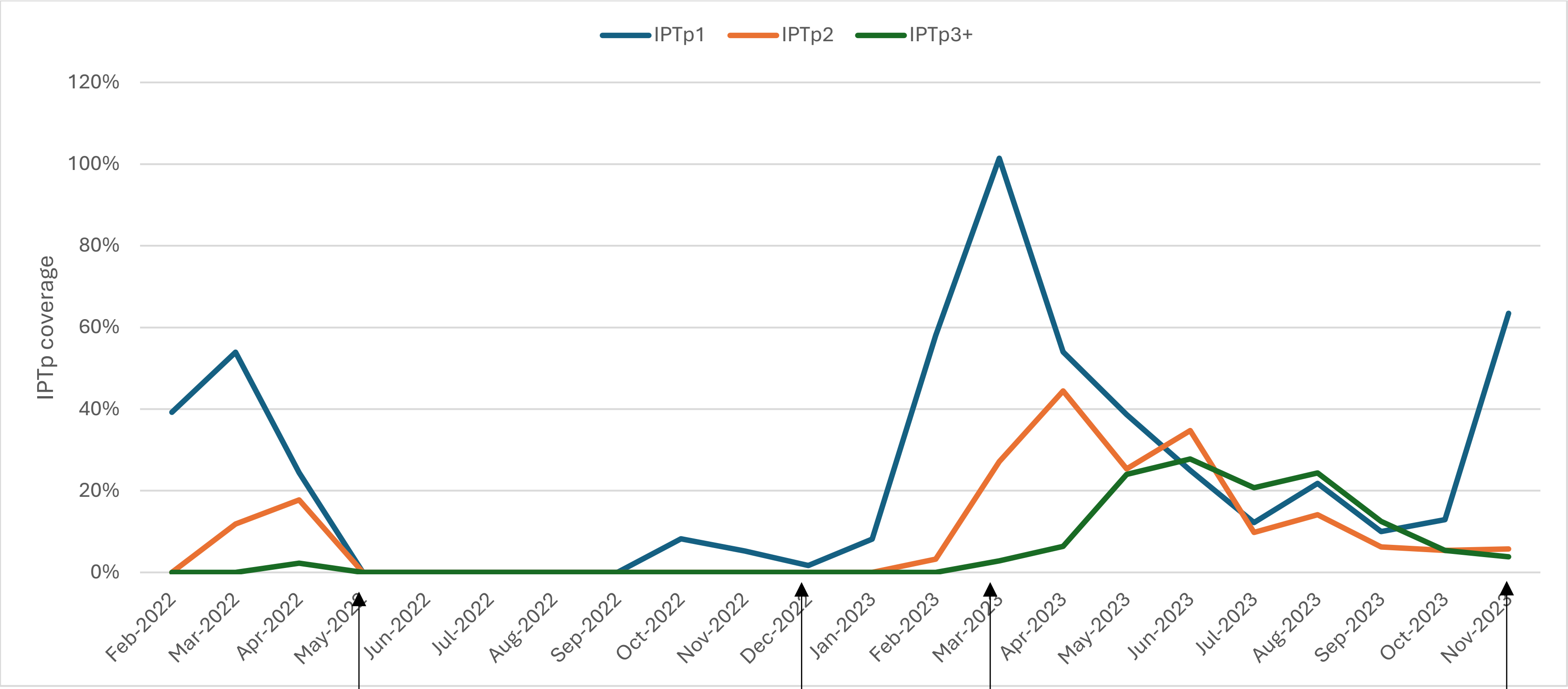

Figure S10 Health facility 7 IPTp-DP coverage and CQI notes

CQI 1
Monitor: Administratively, IPTp-DP was prepared with available decree and SOPs, but IPTp-DP participation lagged behind targets.
Improve: The IPTp-DP service process was initiated with drug provision and educational sessions at the main health centre and Integrated health posts.
Define: High dropout rates after IPTp-DP 1 were due to severe nausea and vomiting, coupled with limited drug supplies and educational media.
Plan: Enhanced communication, community education, and logistic availability were prioritised to address participation drop-offs.

CQI 2
Monitor: IPTp-DP restarted in October 2022.
Improve: Ongoing education and monitoring were planned to address participants' concerns, particularly regarding nausea and vomiting
Define: Medication stockouts and reluctance to continue with IPTp-DP due to side effects were significant barriers.
Plan: Increased counselling frequency and ensuring consistent drug availability were identified as necessary actions.

CQI 3
Monitor: Key IPTp-DP indicators showed low achievements, and even lower results for subsequent indicators.
Improve: Education sessions and antenatal classes were planned at Integrated health posts to improve awareness and participation.
Define: Persistent issues with nausea, vomiting, and lack of familial support continued to hinder IPTp-DP uptake.
Plan: Enhanced education, community engagement, and better communication about IPTp-DP benefits were emphasised.

CQI 4
Monitor: IPTp-DP 1 coverage increased, but subsequent doses saw further declines, with screening guidelines followed consistently.
Improve Efforts focused on engaging pregnant women and their families, and addressing common side effects to ensure IPTp-DP completion.
Define: Inconsistent husband participation during antenatal visits and low acceptance of IPTp-DP among new mothers remained challenges.
Plan: Direct engagement with families, leveraging community leaders, and targeted education on managing side effects were implemented.

Health facility performance of IPTp-DP coverage and CQI data

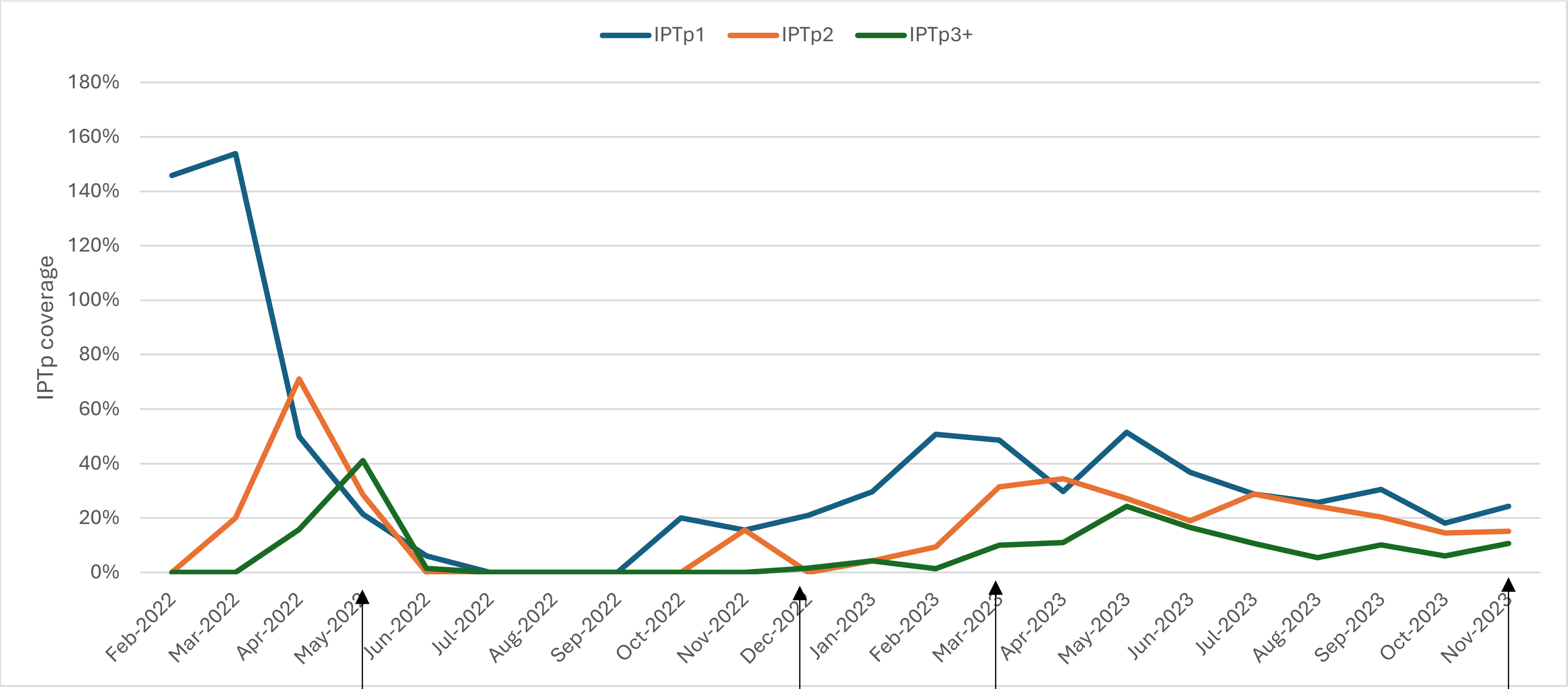

Figure S11 Health facility 8 IPTp-DP coverage and CQI notes

CQI 1
Monitor: IPTp-DP was initiated with available decrees and logistics, achieving a high initial participation rate in March 2022.
Improve: Providing educational materials and starting the program with pregnant women at the health centre and Integrated Health Posts.
Define: Participation rates dropped significantly in April due to complaints from pregnant women and a lack of follow-up, and by limited stock of Anti-Malaria Medication.
Plan: Increased counselling, community education, and better logistics management were identified as necessary steps to improve participation.

CQI 2
Monitor: IPTp-DP resumed in October 2022
Improve: Efforts were made to provide ongoing counselling and to address concerns about side effects, with education being provided to both women and their families.
Define: The main issues included persistent complaints from pregnant women and a lack of consistent engagement from staff.
Plan: Enhanced communication and systematic follow-up were prioritized to address the identified challenges.

CQI 3
Monitor: A gradual increase in participation rates for IPTp-DP was observed from December 2022 to March 2023, with a notable improvement in IPTp-DP 1 coverage.
Improve: Providing frequent education and counselling sessions, leveraging community leaders, and simplifying the information provided.
Define: The uptake for IPTp-DP 2 and beyond remained low, mainly due to late presentation of pregnant women and challenges in communication.
Plan: Increasing early participation and addressing communication gaps through structured educational campaigns.

CQI 4
Monitor: Further challenges were noted in maintaining the subsequent doses.
Improve: Continuous efforts to engage both pregnant women and their families, along with improved management of medication side effects.
Define: The main challenge remained the drop-off in participation after the first dose, particularly due to side effects and the absence of husbands during the decision-making process.
Plan: Continued community engagement, direct education, and ensuring clear communication about the benefits and side effects of IPTp-DP.

Health facility performance of IPTp-DP coverage and CQI data

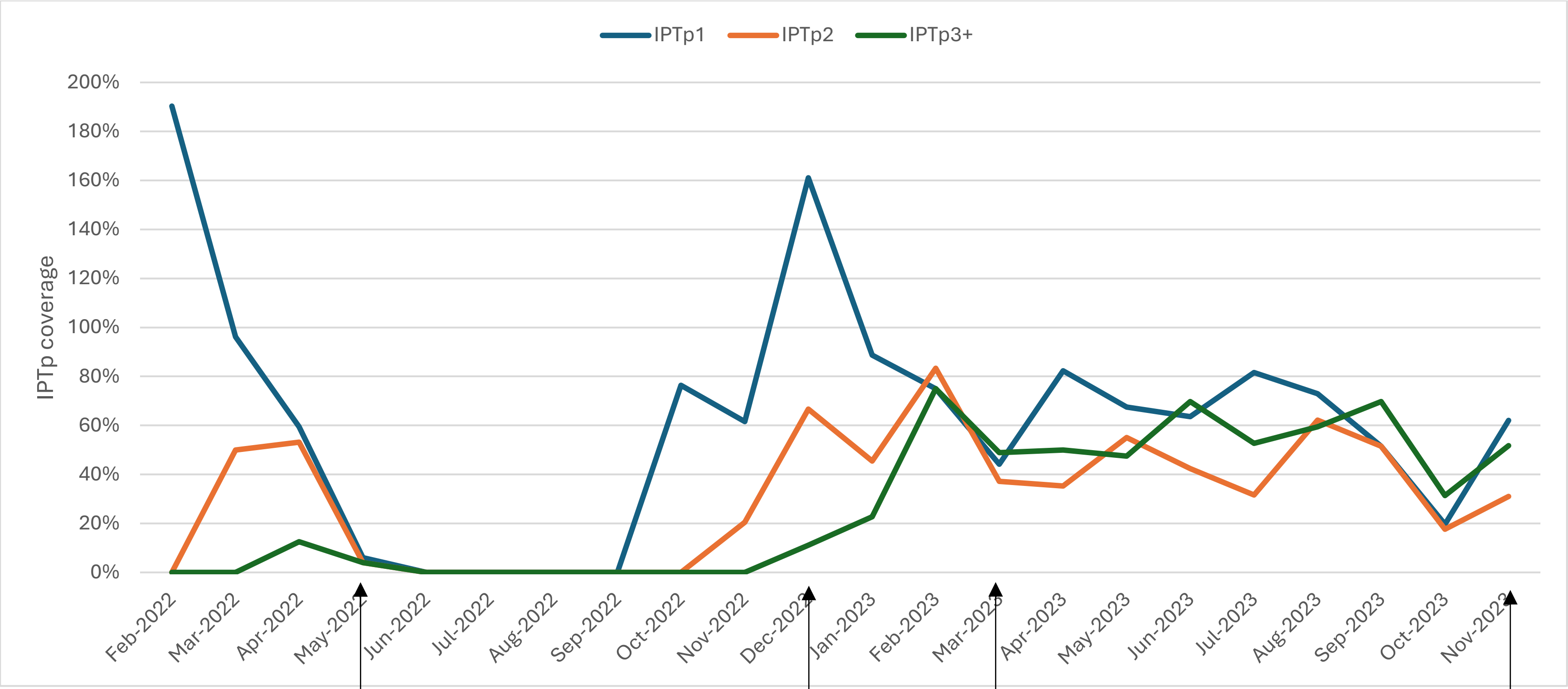

Figure S12 Health facility 9 IPTp-DP coverage and CQI notes

CQI 1
Monitor: IPTp-DP was initiated with the necessary decrees, SOPs, and logistics, achieving high participation among pregnant women.
Improve: Cross-sector program education were conducted during antenatal classes and at Integrated Health Posts.
Define: Resistance from pregnant women, insufficient human resources, and frequent stockouts of DHP were significant obstacles.
Plan: Continued campaign to healthcare to address misunderstandings and improve participation.

CQI 2
Monitor: IPTp-DP indicators showed improvement in IPTp-DP 1 achievement, but subsequent doses lagged significantly behind targets.
Improve: Education and counselling were prioritised, with efforts to encourage compliance through monitoring and engagement at Integrated Health Posts.
Define: High dropout rates after the first dose, coupled with side effects and preference for home administration.
Plan: IPTp-DP education, particularly focusing on addressing side effects and enhancing understanding among healthcare workers and patients

CQI 3
Monitor: High participation between December 2022 to February 2023.
Improve: A combination of field monitoring, compliance tracking, and education campaigns was employed to boost participation and address the decline in subsequent doses.
Define: Persistent issues with side effects, lack of family support, and inconsistent messaging from healthcare workers remained barriers
Plan: Strengthened communication, and targeted education to address the concerns of both pregnant women and healthcare workers.

CQI 4
Monitor: The coverage for IPTp-DP 2 and beyond was minimal, with significant drop-offs noted.
Improve: Enhancing awareness through antenatal classes, Integrated Health Posts, and home visits, with an emphasis on ensuring medication adherence.
Define: Issues included resistance from some healthcare workers, environmental factors affecting patient participation, and insufficient resources to manage the program effectively.
Plan: Continued education, consistent monitoring, and addressing the specific concerns related to side effects and family support

Health facility performance of IPTp-DP coverage and CQI data

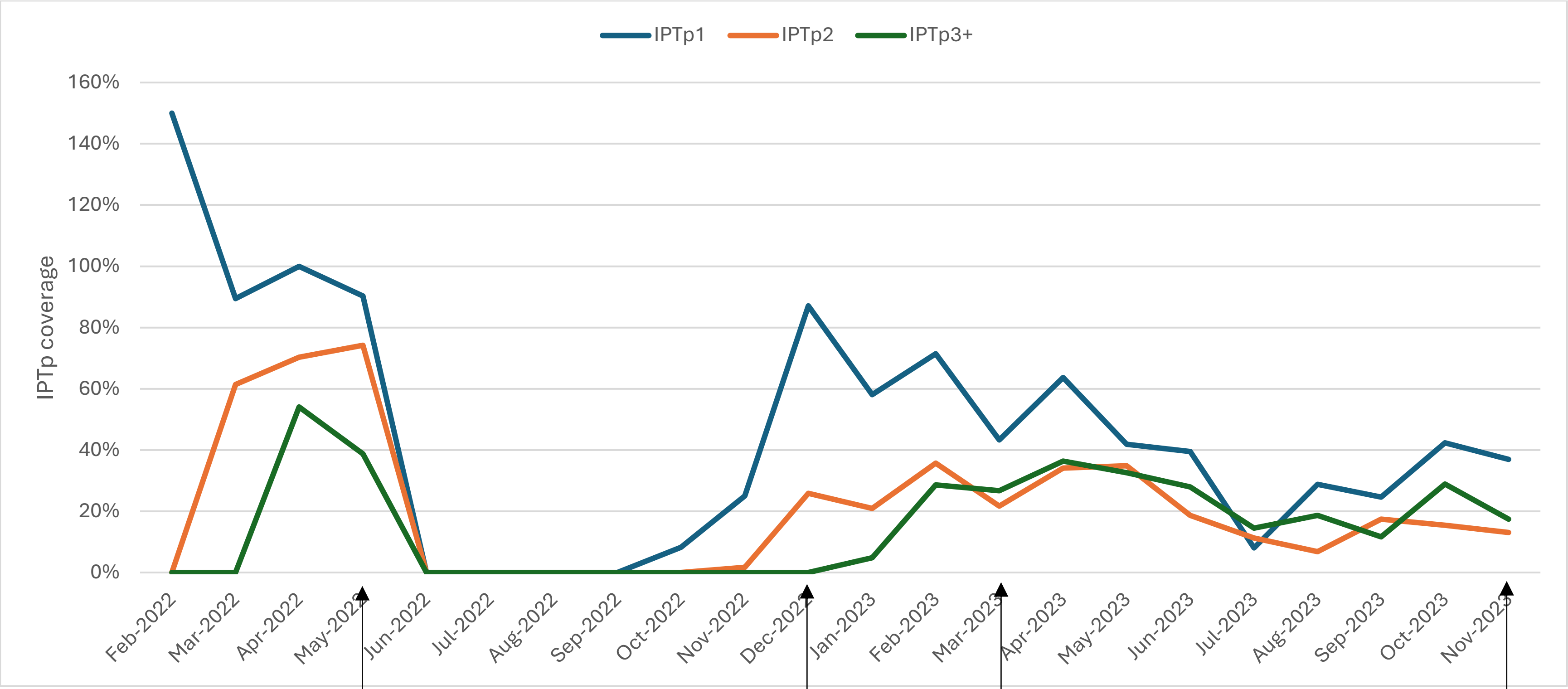

Figure S13 Health facility 10 IPTp-DP coverage and CQI notes

CQI 1
Monitor: IPTp-DP program was successfully initiated at multiple locations, with administrative completeness including decree, SOPs, and service flow.
Improve: Campaign efforts included face-to-face meetings, social media campaigns, and cross-sectoral collaborations, alongside the establishment of recording system.
Define: Inadequate facilities at Posyandu, shortage of staff for home visits, and resistance from pregnant women due to fear of medication side effects.
Plan: Targeted education strategies to improve understanding and acceptance of IPTp-DP among the community.

CQI 2
Monitor: The program restart in October 2022, participation remained low
Improve: Continued efforts in education, involving all midwives and leveraging social media, were implemented to address participation gaps.
Define: Persistent issues with medication side effects, lack of family support, and insufficient promotional materials at health posts
Plan: Focused education on managing side effects, enhanced promotion, and increased involvement of community leaders and community health workers were identified

CQI 3
Monitor: IPTp-DP participation data showed gradual improvement, but still fell short of targets, with significant drop-offs in subsequent doses.
Improve: Involvement of role models was employed to encourage participation and address misconceptions.
Define: The program continued to struggle with low uptake due to fears of side effects and a lack of mandatory participation regulations.
Plan: Introduction of a policy mandating participation in IPTp-DP and increased efforts to involve families and the private sector were recommended to enhance program success.

CQI 4
Monitor: IPTp-DP coverage showed slight improvement.
Improve: Promoting IPTp-DP through community engagement, with an emphasis on using role models and addressing root causes such as lack of family support.
Define: Insufficient public awareness, and logistical issues, including the unavailability of banners and educational materials.
Plan: Continued emphasis on education, cross-program collaboration, and targeted campaign as key strategies

Algorithm of IPTp-DP services. Source: Mimika District Health Office (2022)

Step 1: The first antenatal care (ANC) visit for pregnant women under 13 weeks' gestation includes malaria single screening and treatment (SST). If tested positive, following the *Guidelines for Malaria Management in Pregnant Women* based on KMK No. HK.01.07/MENKES/556/2019. If gestational age is ≥13 weeks, SST is not conducted.

Step 2: If a pregnant woman has symptoms and is diagnosed with malaria, treatment is provided in accordance with the *Guidelines for Malaria Management in Pregnant Women* based on KMK No. HK.01.07/MENKES/556/2019.

Step 3: To ensure the safe administration of preventive dihydroartemisinin-piperaquine (DP) in IPTp-DP services, pregnant women must not receive preventive DP if:

- Gestational age is under 13 weeks.
- Have taken DP (either as treatment or prevention) in the past 4 weeks.
- Have a history of syncope (fainting) or seizures within 24 hours after taking DP.
- Have a known allergy to DP.

To prevent malaria during pregnancy, women are provided with insecticide-treated nets (ITNs), encouraged to seek prompt diagnosis, and given appropriate malaria treatment if tested positive.

Step 4: Pregnant women who meet safety criteria are administered preventive DP, with reminders to return at least every 4 weeks for continued preventive care.

Source: Mimika District Health Office. 2022. *Policy on Malaria Prevention in Pregnant Women through Preventive DHP Administration in Mimika District.* Decree No. 443.41/89/2022. Mimika: District Health Office.

TIDieR (Template for Intervention Description and Replication) Checklist*:

Information to include when describing an intervention and the location of the information

Item number	Item	Where located **	
		Primary paper (page or appendix number)	Other † (details)
1.	**BRIEF NAME**<br>Evaluation of a pilot implementation of intermittent preventive treatment with dihydroartemisinin-piperaquine to prevent adverse birth outcomes in Papua, Indonesia.	Study protocol p1	______________
2.	**WHY**<br>Malaria during pregnancy causes severe maternal and infant health issues. Intermittent preventive treatment in pregnancy (IPTp) with monthly doses of dihydroartemisinin-piperaquine (DP) was shown to be highly effective in preventing malaria in pregnant women in a trial in Papua. The Ministry of Health therefore piloted IPTp-DP in selected health facilities in Papua from 7 February 2022 to 30 November 2023. The pilot was accompanied by an evaluation to determine the effectiveness, acceptability, feasibility/scalability, and cost-effectiveness in a real life setting.	Study protocol p3-6	______________
3.	**WHAT**<br>Materials: The study supported the Ministry of Health (MOH) to pilot IPTp-DP through routine antenatal care (ANC) services in ten community health facilities in Timika through - training of healthcare providers, continuous quality improvement (CQI), pharmacovigilance, and effective communication. Official decrees from the District Health Office, guidelines, and instructional videos on technical service delivery, and standard operating procedures (SOPs) were co-developed to		https://drive.google.com/open?id=1DrVoZGSTxUzDmO3JwMqTWwRSVpG0MP

	support implementation. These included job aids, information leaflets, X-banners, flipcharts, IPTp-DP stickers for MCH books, and reminders for pregnant women to return for follow-up visits. The study team also co-developed community health promotion media, such as videos, to be disseminated via social media and public spaces. Job aids such as flipcharts and leaflets were used to provide information to pregnant women, and information system materials, including stickers as reminders for pregnant women, were also distributed.<br><br>Dihydroartemisinin-piperaquine (DP) was provided by the MOH. However, during periods of limited DP supply (October 2022 to April 2023), the research project supplied DP for preventive treatment, while the Ministry of Health's stock was used for treatment purposes. To support routine monitoring in the health management information system (HMIS), adaptations were made to routine maternal health reporting on maternal health cards and monthly reports, including forms for recording any adverse events.		X5&usp=drive_fs
**4.**	Procedures: The delivery of IPTp-DP at ANC involved several key steps. Pregnant women in their second and third trimesters were screened for eligibility to receive the IPTp-DP intervention. The fixed dose DP regimen (dihydroartemisinin 40 mg, piperaquine phosphate 320 mg) included 3 tablets per day for 3 days. The first dose of DP was to be administered through Directly Observed Therapy (DOT) at the Community health centres, while the second and third doses were given to pregnant women to take at home. In the event, different strategies were employed to ensure adherence by each? of the Community health centres: pregnant women were visited on the second and third days to administer the doses, or Community health centres staff followed up with pregnant women via telephone to ensure they took their medication.	Study protocol p21-22	______________

	Five quality improvement workshops were conducted by staff involved in IPTp services (see 5) in each health facility over the course of the pilot to enhance service delivery, including routine reporting and monitoring. Additionally, training on effective communication and pharmacovigilance was provided to support the intervention activities.		
**5.**	**WHO PROVIDED**<br>Community health centres staff that involved in IPTp services: Doctors, Midwives, Pharmacy, Malaria coordinator. Staff who were already involved in providing routine antenatal care services and has responsibility in malaria services in Community health centres, Sub-health centres, and Health posts. These healthcare providers received training to prescribe and administer IPTp-DP. Training included guidelines on the administration of IPTp-DP, safety monitoring, management of side effects, and counselling techniques to improve adherence among pregnant women.<br><br>District Health Office (DHO) Staff. Senior staff from the DHO, supported by the Timika Research Facility, were responsible for overseeing the implementation and ensuring the delivery of IPTp-DP. DHO staff were involved in the training and supervision of ANC staff and midwives, as well as in the development and dissemination of training materials and job aids.<br><br>Study Team. The study team included LSTM and Timika Research Facility staff responsible for monitoring and evaluating the intervention's implementation and outcomes. Researchers received training on safeguarding, Good Clinical Practice (GCP), qualitative research methods, and	Study protocol p1,3,4,5, and 10	______________

	quantitative survey data collection. This comprehensive training ensured that researchers were well-prepared to conduct thorough and ethical evaluations of the intervention<br>**HOW**		
**6.**	The intervention was delivered through a combination of face-to-face interactions and follow-up mechanisms to ensure adherence and effective delivery. Pregnant women in their second and third trimesters were screened for eligibility at Community health centre during Antenatal care services. The first dose of dihydroartemisinin-piperaquine (DP) was administered through Directly Observed Therapy (DOT) at the Community health centre, ensuring that the initial dose was taken correctly under supervision. For the second and third doses, women were given blister packs to take at home. However, in some Community health centre, different strategies were employed to ensure adherence, such as follow up contact to home on the second and third days to administer the subsequent doses, or follow-up calls by Community health centre staff to confirm that the medication was taken as prescribed.<br><br>Information was provided to pregnant women using job aids such as flipcharts and leaflets during their visits to the Community health centre, educating them about the importance of the IPTp-DP regimen and adherence. Stickers were also provided as reminders for pregnant women to return for follow-up visits. These materials supported both individual and group education efforts during antenatal care sessions or health promotion campaign at community level. Additionally, five quality improvement workshops were conducted throughout the project to enhance service delivery, including training on routine reporting and monitoring, effective communication, and pharmacovigilance. These workshops aimed to improve service quality and adherence to the intervention at both individual and group levels.	Study protocol p21-22	______________

	The research component involved measuring effectiveness and adherence through exit interviews and home visits with pregnant women. Acceptance of the IPTp-DP programme was assessed through stakeholder interviews, including pregnant women and healthcare providers, conducted mid-study and at the end of the study.		
**7.**	**WHERE**<br>The intervention was conducted in ten Community health centres and their associated health posts in Timika city. These locations were chosen based on accessibility. The Community health centres provided the necessary infrastructure for delivering antenatal care services and implementing the IPTp-DP intervention.<br><br>The infrastructure included designated areas for counselling and administering the first dose of DP through Directly Observed Therapy (DOT). Additionally, these centres were equipped with the necessary materials and facilities to support the intervention, including job aids, informational leaflets, and quality improvement tools. Follow up were also part of the intervention, where healthcare workers ensured adherence to the medication regimen.<br><br>The locations were selected to maximise the reach and impact of the intervention, leveraging the existing healthcare infrastructure to provide comprehensive care and support to pregnant women in the region.	Study protocol p11-12	______________
**8.**	**WHEN and HOW MUCH**<br>The IPTp-DP intervention was delivered to pregnant women in ten community health centres in Timika city from February 2022 to November 2023. Quality improvement workshops led by MOH	Presentation of preliminary	______________

	were held five times during the pilot (January 2022, May 2022, December 2022, March 2023, November 2023).<br><br>The pilot evaluation involved mixed methods at midline and endline in addition to collection of routine data on coverage and outcomes. At midline qualitative data collection cycle (June to July 2022) to gathered insights into early implementation progress and challenges. At endline, 1) qualitative data collection (May to December 2023) to assess overall acceptability and feasibility of the intervention; 2) Exit Interviews and home visits (May to October 2023) to assess delivery effectiveness and adherence; 4) Routine data on coverage and impact from HMIS records; 5) Implementation process data from CQI workshops; 6) Cost data were collected continuously during implementation for a cost-effectiveness analysis.	dissemination meeting	
	**TAILORING**		
**9.**	The IPTp-DP intervention was to be implemented according to guidelines set out in the decree across all 10 health facilities, with all participants receiving the same treatment during the same period.	IPTp-DP guidelines	______________
	**MODIFICATIONS**		
**10.‡**	Specific strategies were employed to tailor the delivery of IPTp-DP to ensure adherence and address local challenges:<br>1. Second and third dose strategies: Nurse follow up at home and phone Calls<br>In one Community health centre, healthcare workers conducted follow up visits at home on the second and third days to administer the subsequent doses of DP. This approach ensured that the medication was taken correctly and helped address adherence issues.	CQI notes	______________

In all other Community health centres, follow-up calls were made to pregnant women to confirm that they had taken their medication as prescribed. This strategy provided additional support and reminders to enhance adherence.

2. Temporary Halt of IPTp-DP delivery Due to DP Shortage

Due to a national shortage of DP, the intervention was temporarily halted from May to September 2022. The project provided stocks of DP for IPTp between October 2022 to April 2023 to ensure that the intervention could continue effectively once the MOH supply was restored.

HOW WELL

11. Planned:

The study aimed to estimate both delivery effectiveness and adherence to IPTp-DP under routine antenatal care conditions. Delivery effectiveness was anticipated at 75%, meaning that 1,080 women would receive a full course of IPTp-DP out of the total 1,440 enrolled. Among these, adherence to the 3-day regimen was expected to be 60%, based on estimates from previous implementation studies.. Study protocol p14-18

Data of IPTp-DP coverage extracted from the routine HMIS: number of doses of IPTp-DP by period during the implementation.

**12.‡**	Actual:<br><br>Intervention adherence and fidelity were assessed at study endline to assess whether the IPTp-DP intervention was delivered as planned. The adherence and effectiveness results were as follows:<br>- Effectiveness of Delivery: 40.7% of the IPTp-DP doses were delivered effectively (29.4% fully effective and 11.3% partial effective). These results were obtained using exit interview from pregnant women.<br>- Adherence Results: Percentage of Adherence: 90.3% of pregnant women adhered to the IPTp-DP regimen as planned, completing the three-day treatment course. The adherence was obtained through and home visits, which included pill counts and self-reports.	Presentation of preliminary dissemination meeting slide 28	______________

Source: Hoffmann, T. C., et al. (2014). "Better reporting of interventions: template for intervention description and replication (TIDieR) checklist and guide." BMJ : British Medical Journal 348: g1687.

CRISP checklist

Consensus Reporting Items for Studies in Primary Care

CRISP Checklist

The CRISP Checklist[1] can help researchers meet readers' needs by including content that the primary care community feels is important for the validity, quality, and usefulness of primary care research reports.

The Checklist summarizes recommendations for authors to consider in crafting their report. Nothing is intended to limit the effective or creative reporting of research. Authors and editors make final decisions.

Primary care research involves a wide variety of methods, study designs, topics, and settings. The Checklist covers this broad spectrum and therefore, not all items apply to all studies. Some items may not apply to your study. Some may ask for information that is not available. Check such items off and use the rest of the Checklist in whatever way helps you improve *your* report of *your* research.

How to use the CRISP Checklist:

- Each item is listed. Please respond to each item, even if it is not included in your report.
- Check if the item is included in your report: yes, no, or not applicable to your study.
- If the item applies to the study design but is not included in the report, please provide a brief explanation in the "Notes" section.
- The "Suggested Section" indicates where the item usually appears in a research report following the IMRaD format (Introduction, Methods, Results, and Discussion). These are suggestions only. If the item is in a different section of your report, you might add that in "Notes."
- You can note the location of the item in your report by line, page, or section in "Notes."

For more information plus an explanation and examples of each item, please see the supplemental guidance in the Appendix to the CRISP Statement.[2]

You may choose to submit the completed Checklist along with your manuscript to help editors and reviewers see how you have included the suggested items in your research report. Authors should also consider using other reporting guidelines that are appropriate for their study and report. (See Table 2.[1]) Some CRISP items may overlap with other guidelines.

For more information, see **CRISP:** https://crisp-pc.org/

References:

1. Improving the Reporting of Primary Care Research: Consensus Reporting Items for Studies in Primary Care—the CRISP Statement. William R. Phillips, Elizabeth Sturgiss, Paul Glasziou, Tim C. olde Hartman, Aaron M. Orkin, Pallavi Prathivadi, Joanne Reeve, Grant M. Russell, and Chris van Weel. *Annals of Family Medicine* November 2023, 21 (6) 549-555; DOI: https://doi.org/10.1370/afm.3029
2. Supplemental Appendix. Consensus Reporting Items for Studies in Primary Care (CRISP) Explanation and Examples Guide. https://www.annfammed.org/content/annalsfm/suppl/2023/10/02/afm.3029.DC1/Phillips-Supp-App-Table-2023.pdf

CRISP Checklist

Reporting Item - 1	Included?			Section*	Notes
	Y	N	N/A		
**1. Include "primary care" and/or discipline-specific terms in the title, abstract, and/or key words.**	√			I	**In abstract, and keywords**

Reporting Item - 2	Included?			Section*	Notes
	Y	N	N/A		
**2. Describe the study rationale and importance for primary care.**					
**2a.** Explain the rationale for the research question and how it relates to primary care.	√			I	The research question addresses how a new malaria prevention strategy (IPTp-DP) can be integrated into antenatal care (ANC) services delivered through Indonesia's primary care system. As ANC is predominantly accessed at the primary care level, understanding delivery and adherence in this context is essential for informing national scale-up.
**2b.** Describe the importance or relevance of the topic under study in the primary care setting.	√			I	Malaria in pregnancy poses a serious threat in Papua, a region with moderate-to-high malaria transmission. Since most pregnant women in Indonesia receive care from puskesmas (public primary health centres) and their affiliated outreach posts, evaluating how a new intervention is delivered and received in routine primary care settings is critical to ensure accessibility, equity, and impact.
**2c.** Identify any theory, model, or framework used, and explain why it is appropriate to the research question in primary care.		√		I	

Reporting Item - 3	Included?			Section*	Notes
	Y	N	N/A		
**3. Describe the research team's primary care experience and collaboration.**					
**3a.** Describe the research team's expertise and experience in primary care practice and/or research.		√		M	The study team includes public health researchers and clinicians with extensive experience in primary care delivery and implementation research in Indonesia, particularly in maternal and child health and malaria

					control. The pilot was implemented through collaboration with the Mimika District Health Office and frontline ANC providers working in primary care settings
**3b.** Describe whether and how primary care patients, practicing clinicians, community members, or other stakeholders were involved in the research process.	√			M	Stakeholder involvement was central to the design and implementation of the study. Primary care clinicians and district managers were engaged through co-design workshops, CQI meetings, and technical training. Pregnant women and community members were involved through qualitative interviews and FGDs to ensure the intervention was grounded in community preferences and experiences

Reporting Item - 4	Included?			Section*	Notes
	Y	N	N/A		
**4. Describe the study participants and populations in the context of primary care.**					
**4a.** Use person-focused language to refer to the research populations and participants, or use terms based on patient preferences	√			R	The manuscript consistently uses person-focused language such as “pregnant women,” “participants,” and “health workers” rather than clinical or de-personalised terms**.**
**4b.** If reporting personal characteristics of participants, report the source of the data, the rationale for using it, and the rationale for any classifications used.	√			R	Sociodemographic and clinical data were self-reported by participants or extracted from maternal health cards during interviews. Classifications such as education level or gravidity were used based on standard national reporting categories and contextual relevance
**4c.** Describe the participants and populations in sufficient detail to allow comparison to other primary care patient populations.	√			R	Health facilities were described in detail, allowing comparison
**4d.** Specify whether participants have pre-existing therapeutic relationships with the clinical team or are new patients.		√		M/R	Participants were pregnant women attending routine ANC visits at public primary health facilities. Most were attending

					scheduled visits under Indonesia's standard maternal care schedule and had existing relationships with midwives providing care, though a small proportion may have been new attendees.

Reporting Item - 5	Included?			Section*	Notes
	Y	N	N/A		
**5. Describe the conditions under study in the context of primary care.**					
**5a.** Describe whether the condition under study is acute or chronic.		√		M/R	The condition under study is malaria in pregnancy, which is an acute infectious disease that poses episodic but significant risk during pregnancy, including maternal anaemia, premature birth, stillbirth, and low birthweight infants.
**5b.** Report how multimorbidity is considered and how it might affect interpretation of the study findings/ results.		√		M	Multimorbidity was not a primary focus of this study, and comorbid conditions were not systematically assessed.

Reporting Item - 6	Included?			Section*	Notes
	Y	N	N/A		
**6. Describe the clinical encounter under study in the context of primary care.**					
**6a.** Specify whether the study focus is an isolated clinical encounter or a longitudinal course of care. If it is an isolated clinical encounter, specify whether it is the first visit or a follow-up visit for the condition under study	√			M	The study focused on both isolated. Specifically:<br><br>Delivery effectiveness was assessed through exit interviews following individual ANC visits (which may be a first or follow-up visit).<br><br>Adherence was assessed longitudinally for each course of IPTp-DP, including home visits 3–4 days after the clinic visit to evaluate completion of the 3-day regimen.

Reporting Item - 7	Included?			Section*	Notes
	Y	N	N/A		
**7. Describe the patient care team.**					
**7a.** If care is delivered by teams, describe the team members and their roles.			√	R	Care was delivered by a primary care teams at community health centres.
**7b.** For each clinician category, report profession, specialty, and qualifications.		√		R	The main clinicians involved were:<br><br>Midwives – Diploma-qualified health professionals trained in maternal and reproductive health, responsible for providing ANC and implementing IPTp-DP.<br><br>CHWs (kader) – lay community workers with basic health training, supporting health promotion and outreach.<br><br>Health centre nurses – typically diploma holders in nursing, supporting midwives in clinical service delivery.

Reporting Item - 8	Included?			Section*	Notes
	Y	N	N/A		
**8. Describe the study interventions in the context of primary care.**					
**8a.** Describe interventions and their implementation in sufficient detail to enable the reader to assess applicability in their own setting.	√			M	The intervention (IPTp-DP) was implemented through routine ANC services in primary care

					settings (puskesmas, pustu, posyandu). Midwives delivered the first dose by DOT, and women took the remaining doses at home.
**8b.** Describe any clustering or grouping of patients, participants, clinicians, teams, or practices, and how it was addressed in the analysis.	√			M/R	Participants were nested within 10 primary health facilities and their catchment areas. The analysis accounted for this clustering at the facility level using robust standard errors and facility-level variables
**8c.** Describe the health care system in sufficient detail to allow comparisons to other systems.	√			I/D	The study was conducted within the Indonesian public primary healthcare system (Puskesmas and its networks), which is publicly funded and serves as the frontline for ANC and useful in comparative context.

Reporting Item - 9	Included?			Section*	Notes
	Y	N	N/A		
**9. Describe study measures used and their relevance to primary care.**					
**9a.** Report whether study measurement tools have been validated in primary care populations or settings.	√			M	Measurement tools were adapted from previously used instruments in malaria in pregnancy studies in similar LMIC settings. While the tools were not formally psychometrically validated in primary care populations, content validity was established through expert review and pretesting with ANC users.
**9b.** Describe how the measurement tools used are meaningful to primary care patients and their care.	√			M	The questions were developed in close collaboration with local stakeholders to ensure that the information collected was relevant and actionable
**9c.** Report findings/results to be clinically interpretable by primary care clinicians and patients.	√			R	Findings were reported in ways that are clinically interpretable: % of women who received effective delivery of IPTp-DP, % of adherence, coverage, and qualitative themes.

	Included?		

Reporting Item - 10	Y	N	N/A	Section*	Notes
**10. Discuss the meaning of study findings/results in the context of primary care.**					
**10a.** Discuss implications of the study findings/results for research, patient care, education, and policy with specific focus on primary care.	√			D	The discussion addresses how IPTp-DP can be feasibly integrated into routine antenatal care (ANC) at the primary care level, with implications for programme rollout and policy in malaria-endemic settings. The findings support scaling up IPTp-DP in similar settings where malaria in pregnancy remains a concern. This is especially relevant for midwives and primary care providers, who are the main actors in ANC delivery and patient counselling
**10b.** Discuss the implications of study recommendations on demands and priorities in primary care practice.	√			D	Study recommendations highlight the need for supportive supervision, provider training, and logistics which have direct implications for primary care workloads, priorities, and service delivery models.
**10c.** Comment on any research processes that might influence the applicability of the study findings/results in diverse primary care settings.	√			D	The discussion acknowledges that the pilot was conducted in urban and semi-urban primary care settings, and adaptations may be necessary to scale up the intervention to more remote or resource-limited primary care environments. It also reflects on how study support may have enhanced outcomes, and that real-world effectiveness may vary once scaled.

***Section:** I = Introduction, M = Method, R = Results, D = Discussion

ASSESS guidance

**Review or meta-analysis question**		Not applicable – this is a primary mixed methods implementation research study, not a review or meta-analysis.				**NOTES**
**ARTICLE CITATION**						
**Study author, publication year**		Firdaus Hafidz, Freis Candrawati, Jenna Hoyt, Enny Kenangalem, James Dodd, Maia Lesosky, Ida Safitri Laksanawati, Reynold Ubra, Minerva Simatupang, Feiko O ter Kuile, Eve Worrall, Jeanne Rini Poespoprodjo, Jenny Hill. Publication year (anticipated): 2025				
**Study title**		Pilot implementation of intermittent preventive treatment with dihydroartemisinin-piperaquine to prevent adverse birth outcomes in Papua, Indonesia: A mixed method evaluation				
**INTRODUCTION**		**Implementation strategy**	**Reported on page #**	**Intervention**	**Reported on page #**	
**Rationale**		The implementation strategy focused on integrating IPTp-DP delivery within routine ANC services in Papua, Indonesia, through a combination of training, stakeholder engagement, CQI processes, routine data strengthening, and supportive supervision.	**4**	IPTp-DP given to pregnant women to prevent malaria and improve maternal outcomes.	4	Intervention is described in the introduction, and elaborated in methods, on how IPTp pilot programme implementation
**Aim(s), objective(s), or research question(s)**	Co-Primary	to evaluate the pregnant women's adherence to IPTp-DP	4	not applicable		We opted to re-label adherence and delivery effectiveness as co-primary outcomes since they were interdependent
	Co-Primary	delivery effectiveness of through routine ANC services	4	not applicable		
**METHODS: DESCRIPTION**			**Reported on page #**		**Reported on page #**	
**Descriptions**		Training for ANC providers and facility heads on IPTp-DP delivery and pharmacovigilance.<br>Stakeholder and community engagement (e.g., community health workers, district leaders).<br>Community engagement	5	Administration of dihydroartemisinin-piperaquine (DP) starting from the second trimester (≥13 weeks gestation).<br>Monthly doses of 3 tablets/day for 3 consecutive days (9 tablets/course).<br>First dose given under directly observed therapy (DOT) by ANC staff.<br>Expected to have three courses targeted during pregnancy.	5	More detail in TIDieR check list in appendix
**Adaptation**		CQI approach adapted: While originally designed as structured CQI cycles, the implementation was simplified problem-solving meetings rather than full CQI workshops,<br>Adaptations of the current health information system and pharmacovigilance structure	5	Mode of follow-up (e.g., home visits, phone calls).<br>Communication and counselling strategies tailored to local context.<br>DOT flexibility: While the protocol specified that the first dose be administered under DOT, in some cases this was not consistently applied—particularly during fasting periods or when waiting times were long.	5	
**Design**		This study used a mixed-methods evaluation	4	The intervention was embedded within routine ANC services	5	
**Participant types**		Beneficiaries: Ministry of Health Office (MOH), Provincial/ District Health Office (PHO/ DHO)<br>Implementer: study team<br>Respondents: Health providers, health managers, pregnant women, husband of pregnant women, community health workers, community leaders	6	Beneficiaries: Pregnant women attending ANC who were eligible to receive IPTp-DP in The pilot programme was rolled out in the 10 facilities	5	
**Comparison group**		not applicable		Not applicable4		
**Context**		The strategy relied on integrating IPTp-DP into routine ANC workflows in 10 urban and semi-urban public facilities<br>Outside the pilot implementation, prescribe DP without lab evidence is prohibited.	5	DP is the national first-line treatment for malaria, which meant that stock prioritisation during the COVID-19 pandemic disrupted preventive use, leading to a 5-month stock-out.<br>IPTp-DP as a new malaria prevention intervention strategy in the second and third trimester is the addition to SST in the first trimester	5	
**Sites**		Ten community health centres, Mimika district, Mimika district health office, Provincial health office, and Ministry of Health	5	The pilot was implemented in 10 out of 26 community health centres (puskesmas) in Mimika District, Papua, Indonesia.	5	

**Subgroups** (optional)		not applicable			Not applicable				
**Implementation phase**		- Pre-implementation (preparation and training) (Aug 2021-Jan 2022)<br>- Implementation (Support, and monitoring) (Feb 2022 - Nov 2023)<br>- Post-implementation (dissemination) (Dec 2023 – going forward)		4-5	- Start intervention (Feb 2022)<br>'- Temporary interruption (June- Sep 2022)<br>'- Restart and ongoing intervention (Oct 2022 to Nov 2023)			5	
**Process evaluation**		The process evaluation aimed to understand how the implementation strategy functioned to support IPTp-DP delivery. This included evaluating:<br>-Health worker training and communication strategies,<br>-Implementation of CQI mechanisms (e.g. goal setting, local problem-solving),<br>-Drug supply and stock management,<br>-Routine data monitoring,<br>-Provider and pregnant women's experience and acceptability.		5-6					
**Sample size**		Quantitative component: The sample size for the exit interviews was calculated based on an expected adherence rate of 75%, with 95% confidence and ±5% margin of error, adjusting for design effect of 4.2 to account for clustering at the facility level. The resulting target was 1,080 women. However, due to lower-than-expected full delivery effectiveness, the final number of women followed up at home for adherence assessment was 484.<br><br>Qualitative component: For qualitative interviews, purposive sampling was used based on stakeholder role (e.g. health workers, policy makers, pregnant women). The aim was to capture variation in experience across urban and semi-urban facilities. Data collection continued until thematic saturation was reached. Midline and endline interviews were conducted, including FGDs with community stakeholders at endline.		6					
**Analysis**		Quantitative component: Quantitative data were analysed using descriptive statistics to summarise IPTp-DP delivery and adherence outcomes. Logistic regression was used to explore predictors of full adherence and effective delivery. Variables for inclusion were informed by previous literature and contextual relevance.<br><br>Qualitative component: Thematic analysis was conducted on transcribed qualitative data using a health system and acceptability framework approach. Coding was both deductive (guided by the implementation framework) and inductive (emerging from the data). NVivo software was used for data management. Themes were triangulated with quantitative findings to provide explanatory insights into implementation barriers and facilitators.		8-9					
Sub-group analyses		not applicable							
		**Quantitative**	**Qualitative**			**Quantitative**	**Qualitative**		

**Outcomes (assessment)**	*Acceptability*		Assessed through in-depth interviews with pregnant women and health workers regarding the acceptability of IPTp-DP	6	*Effectiveness*	Co-Primary outcome: adherence to a 3-day IPTp-DP regimen; delivery effectiveness. Both assessed through structured exit interviews and home visits.	interviews explored women's and providers' perspectives on the success of IPTp-DP implementatio n	6	The qualitative analyses will be reported in detail in a separate qualitative paper
(not all of these are required)	*Appropriateness*		Perceptions from providers and managers regarding the suitability and relevance of IPTp-DP in their service context	6	*Efficiency*	not measured	not measured		
	Adoption		Descriptions by health workers and facility managers on decision-making to adopt IPTp-DP	6	*Equity*	not measured	not measured		
	Feasibility	IPTp-DP coverage per first ANC visit	Challenges reported in interviews	7	*Patient centeredness*	not measured	Explored in interviews with pregnant women on their preferences, values, and experiences related to IPTp-DP use.	6	
	Fidelity	Fidelity to deliver IPTP-DP as guidelines	Health worker explanations of deviations from the protocol	7	*Safety*	Not a primary focus of this paper. However, pharmacovigilance mechanisms were in place and adverse events were monitored.	Explored in interviews with pregnant women and health workers	5	
	Penetration		Integration processes described by facility managers and providers	6	*Timeliness*	not measured	not measured		
	Cost	N/A (Will be reported as a separate cost-effectiveness study)	N/A (Will be reported in a separate qualitative paper)						
	Sustainability		N/A (Will be reported in a separate qualitative paper)						
**RESULTS: DESCRIPTION**				**Reported on page #**					**NOTES**
		**Quantitative**	**Qualitative**			**Quantitative**	**Qualitative**		
**Outcomes (findings)**	*Acceptability*	not measured	Health workers were generally supportive of IPTp-	*10*	*Effectiveness*	Adherence among those receiving full effective delivery was	Interviews highlighted that some women	9	The qualitative outcomes will be reported in detail in a separate qualitative paper

			DP introduction but reported community-level hesitation due to lack of familiarity with presumptive treatment. Women expressed mixed views, often influenced by providers' explanations.			90.3% (437/484). Among 1,366 pregnant women attending ANC, 402/1,366 (29.4%) received full effective delivery of IPTp-DP (with DOT), and only 154/1,366 (11.3%) received partial effective delivery (without DOT)	were reluctant to take IPTp-DP without malaria symptoms or a confirmed test, affecting perceived effectiveness		
(not all of these are required)	*Appropriateness*	not measured	Stakeholders expressed concern about using DP without testing, indicating a cultural misfit with the existing “test-before-treat” approach. Providers highlighted alignment challenges with current ANC routines.	10	*Efficiency*	not measured	not measured		
	Adoption	not measured	CQI discussions and IDIs revealed that adoption varied by facility, with better uptake where midwives had higher confidence and supportive management.	10	*Equity*	not measured	not measured		
	Feasibility	Overall, the average coverage for IPTp1, IPTp2, and IPTp3+ across all facilities from February 2022 to November 2023 was 39%, 20%, and 15%, respectively	Providers noted barriers such as logistics, staffing shortages that impeded DOT and consistent delivery.	9	*Patien centeredness*	not measured	by health providers’ limited communication skills and lack of engagement with male partners	10	
	Fidelity	not measured	CQI notes and interviews identified failure to conduct DOT, keep doing SST in the second and third trimester in initial phase of the implementation		*Safety*	Not a primary focus of this paper. However, pharmacovigilance mechanisms were in place and adverse events were monitored.	Safety concerns were voiced by providers and some women, especially related to side effect	10	
	Penetration	not measured	Given the limited number of health workers can be trained, thus often many other health workers have not		*Timeliness*	not measured	not measured		

				been reached by refresher trainings									
	Cost	Not reported in the primary manuscript (analysed in a separate cost-effectiveness study)		Not explored qualitatively in this paper									
	Sustainability	not measured		not measured									
**Barriers to implementation**		Several barriers challenged the successful implementation of IPTp-DP during the pilot:<br>- Drug stock-out: A prolonged stock-out of dihydroartemisinin–piperaquine (DP) during the early phase significantly disrupted service delivery, reduced coverage, and eroded trust among both providers and pregnant women.<br>- Provider hesitancy: in the beginning of the pilot, some health workers were reluctant to deliver IPTp-DP without a confirmed malaria diagnosis, reflecting residual preference for the single screen-and-treat (SST) approach and concern over possible drug side effects or resistance.<br>- Complex regimen and resources constraints: The three-day dosing regimen of IPTp-DP posed logistical challenges for directly observed therapy (DOT), particularly in facilities with limited staffing, and resources to follow up given high patient volumes<br>- Communication gaps: Lack of effective communication and understanding among some health workers and patients about the preventive nature of IPTp-DP impacted uptake and adherence.				10-11							
Facilitators of implementation		Several factors were identified as facilitators of successful implementation of IPTp-DP in the pilot:<br>- Supportive policy environment: Strong commitment from the Ministry of Health and local stakeholders provided legitimacy and momentum for the pilot.<br>- Midwife engagement and motivation: Health workers, especially midwives, were generally supportive of the intervention and keen to deliver IPTp-DP once trained and confident.<br>-Training and CQI workshops: Ongoing capacity building through CQI and communication training sessions strengthened provider knowledge, troubleshooting, and peer learning.<br>- Supportive from husband/ family: Women who received support from husband/family reported high adherence and encouraging uptake.				10-11							
**METHODS: EVALUATION**													**NOTES**
		**Implementation strategy**					**Intervention**						
**Design (Follow steps 1, 2, and 3)**		**Step 2: insert 5 corresponding criteria from instructions**	**Step 3: Provide score (0 or 1)**				**Step 2: insert 5 corresponding criteria from instructions**		**Step 3: Provide score (0 or 1) to each criteria**				

			**to each criteria**									
Step 1: Insert design type: Mixed methods		*4.1. Is there an adequate rationale for using a mixed method design to address the research question?*	1				*insert criteria 1*					Yes. The study aimed to assess both the effectiveness and implementation of IPTp-DP delivery, requiring both quantitative data (e.g. coverage, adherence) and qualitative insights (e.g. barriers, acceptability, provider behaviour) to explain why certain patterns were observed.
		4.2. Are the different components of the study effectively integrated to answer the research question?	1				*insert criteria 2*					The quantitative and qualitative components were integrated at both the design and interpretation stages. For instance, qualitative data were used to interpret quantitative findings on adherence and delivery effectiveness (e.g. issues with DOT, hesitancy among women, logistic challenges). This integration helped explain observed trends and variation
		4.3. Are the outputs of the integration of qualitative and quantitative components adequately interpreted?	1				*insert criteria 3*					The discussion synthesises both quantitative results (e.g. low effective delivery) and qualitative insights (e.g. providers' understanding, women's perceptions, systemic challenges). The current interpretation already demonstrates meaningful use of both data types to draw conclusions.
		4.4. Are divergences and inconsistencies between quantitative and qualitative results adequately addressed?	1				*insert criteria 4*					Yes. The manuscript acknowledges areas of divergence—for example, health worker acceptability was previously seen as a barrier, but current findings show greater community-level resistance. This discrepancy is discussed and attributed to context and framing differences.
		4.5. Do the different components of the study adhere to the quality criteria of each tradition of the methods involved?	1				*insert criteria 5*					Yes. The quantitative analysis followed rigorous statistical methods, including logistic regression, sample size justification, and clustering. The qualitative study was guided by a predefined

												protocol, with trained interviewers, thematic analysis, and findings supported by participant quotes (in full manuscript or planned companion paper).
**RESULTS: EVALUATION**												**NOTES**
**Step 4: Sum the score from Step 3 and apply to the outcomes assessed**		**Bias** (1-2=higher bias; 3-5=lower bias; U=unclear; NA=not assessed)		**Step 4: Sum the score from Step 3 and apply to the outcomes assessed**			**Bias** (1-2=higher bias; 3-5=lower bias; U=unclear; NA=not assessed)					
**Outcomes**	*Acceptability*	4		**Outcomes**		*Effectiveness*	*4*					
(not all of these are required)	*Appropriateness*	4		(not all of these are required)		*Efficiency*	*not applicable*					
	Adoption	3				*Equity*	*not applicable*					
	Feasibility	3				*Patient centeredness*	*3*					
	Fidelity	4				*Safety*	*2*					
	Penetration	3				*Timeliness*	*not applicable*					
	Cost	*not applicable*										
	Sustainability	*not applicable*										

STOPMiP-2 study protocol

**Evaluation of a pilot implementation of intermittent preventive treatment with dihydroartemisinin-piperaquine to prevent adverse birth outcomes in Papua, Indonesia**<br><br>***Short Title:*** STOPMiP-2 Indonesia

Identifiers:		
UK LSTM REC [21-054]	Universitas Gadjah Mada [KE/KK/1198/EC/2021]	Clinicaltrials.gov [NCT05294406]

Principal Investigator

- Dr Jenny Hill, MSc, PhD, Liverpool School of Tropical Medicine (LSTM), Pembroke Place, Liverpool L3 5QA, UK jenny.hill@lstmed.ac.uk

Site Principal Investigator

- Dr Jeanne Rini Poespoprodjo, MD, PhD, Centre for Child Health-PRO, Universitas Gadjah Mada (UGM), Jl. Kesehatan no.1, Yogyakarta 55284, Indonesia; didot2266@yahoo.com

Co- investigators UK

- Dr Eve Worrall, PhD, Liverpool School of Tropical Medicine (LSTM), Pembroke Place, Liverpool L3 5QA, UK eve.worrall@lstmed.ac.uk
- Dr Firdaus Hafidz (*Study Coordinator*), PhD, Liverpool School of Tropical Medicine (LSTM), Pembroke Place, Liverpool L3 5QA, UK hafidz.firdaus@lstmed.ac.uk
- Prof Feiko ter Kuile, MD, PhD, Liverpool School of Tropical Medicine (LSTM), Pembroke Place, Liverpool L3 5QA, UK Feiko.terkuile@lstmed.ac.uk
- Prof Maia Lesosky (*Study statistician*), PhD, Liverpool School of Tropical Medicine (LSTM), Pembroke Place, Liverpool L3 5QA, UK Maia.Lesosky@lstmed.ac.uk
- Jenna Hoyt, MSc, Liverpool School of Tropical Medicine (LSTM), Pembroke Place, Liverpool L3 5QA, UK Jenna.Hoyt@lstmed.ac.uk

Co- investigators Indonesia

- Faustina Helena Burdam, MD, Yayasan Pengembangan Kesehatan dan Masyarakat Papua (YPKMP), Jl Yos Sudarso, Timika 99910, Indonesia; lenny_burdam@yahoo.co.id
- Enny Kenangalem, Yayasan Pengembangan Kesehatan dan Masyarakat Papua (YPKMP), Jl Yos Sudarso, Timika 99910, Indonesia; ennykenangalem@yahoo.com
- Freis Candrawati, Yayasan Pengembangan Kesehatan dan Masyarakat Papua (YPKMP), Jl Yos Sudarso, Timika 99910, Indonesia; freisc@yahoo.com
- Ida Safitri Laksanawati, Centre for Child Health-PRO, Universitas Gadjah Mada UGM), Jl. Kesehatan no.1, Yogyakarta 55284, Indonesia; ida_laksono@hotmail.com
- Asal Erlin Mulyadi, Centre for Child Health-PRO, Universitas Gadjah Mada UGM), Jl. Kesehatan no.1,

Funder: MRC, UK
Sponsor: Liverpool School of Tropical Medicine (LSTM); Pembroke Place, Liverpool L3 5QA, UK
Phone: +44 0151 7053794; Email: lstmgov@lstmed.ac.uk

Revision chronology:

Date	Protocol Version	Details of Changes	Authors	PI Signatures
15Jun2021	v1.1	Original	JH, FtK, EW, JRP, FH	
23Aug2021	v2.0	Revisions to ICFs and protocol requested by LSTM REC	JH, FtK, EW, JRP, FH	

21Feb2022	V2.2	Change to drug regimen; revised logic model; Revisions requested by UGM ethics: expanded exclusion criteria; addition of 2018 Data protection Act in all ICFs and of an assent form for pregnant minors (p.31 of ICF Appendix)	JH, FtK, EW, JRP, FH	
10Jul2023	V2.3	Additional respondents added to qualitative data collection and respective ICFs included	JH, JRP, FH	

Confidentiality Statement: This document contains confidential information that must not be disclosed to anyone other than the sponsor, the investigator team, host institution, and relevant ethics committees

1. Technical summary

Malaria in pregnancy is a major cause of maternal and neonatal death in Papua, Indonesia. In our recent trial in Papua, we showed that monthly intermittent preventive treatment (IPTp) with the long-acting artemisinin-based combination dihydroartemisinin-piperaquine (DP) among pregnant women in the second and third trimester was safe, tolerable and more efficacious than the current policy of single screening at ANC booking and treatment of RDT-positive cases. The Ministry of Health (MOH) Indonesia now plans to pilot the strategy in the routine health system in Papua, Indonesia. This study will assess the programme effectiveness of IPTp-DP delivery through antenatal care services and women's adherence to the monthly 3-day DP treatment regimen in a 'real life' setting.

The study will be undertaken in ten community health centres in the lowlands and their associated health posts in Timika city. In the first 18 months, we will support MOH to implement the intervention using quality improvement (QI) approaches to continuously strengthen service delivery, uptake and adherence through plan-do-study-act cycles. We will also support the MoH to collect safety data for pharmacovigilance. A mixed-methods evaluation will be conducted towards the end of the pilot using exit interviews to assess delivery effectiveness, home visits to assess adherence, and qualitative research to explore provider perceptions of the drivers of successful integration, scalability and user acceptability. The primary outcome is adherence and delivery effectiveness, defined as the proportion of pregnant women who receive the first dose of IPTp-DP by DOT at ANC, have received the correct number of DP tablets for subsequent doses, and when visited at home have verified they completed the course. We will also determine the net cost-effectiveness of implementing IPTp-DP and the net cost-effectiveness of the current policy of single screening and treatment (SST) in the routine health system and compare the cost-effectiveness of these alternative options (note: net cost-effectiveness means that cost savings from averted malaria will be deducted from the intervention costs). We will also provide an estimate of the incremental financial cost of implementing IPTp-DP from the provider (MOH) perspective at scale in Papua, Indonesia.

2. Lay summary

The control of malaria in pregnancy in Indonesia, where approximately 10% of pregnant women get infected with malaria, could receive a potential boost through a new study in Papua, Indonesia. A partnership between the Liverpool School of Tropical Medicine (UK) and the Timika Research Facility in Papua Indonesia will conduct a holistic evaluation of a promising new drug-based regimen for preventing malaria and its harmful effects in pregnancy.

When pregnant women contract malaria, the infection can have devastating consequences for pregnancy, resulting in fever which may trigger the preterm onset of labour or even pregnancy loss. It is also possible for women to be infected without showing any outward signs or symptoms, yet if these infections are undetected and left untreated, they can cause severe anaemia in the mother and can interfere with the growth of the fetus leading to low birth weight, which makes babies more vulnerable to infections, growth retardation and dying during the first year of life.

The new study will support the Indonesian Ministry of Health to identify the best way to deliver a new preventive regimen for the control of malaria in pregnancy called intermittent preventive treatment or IPT for short. IPT is used in most countries in Africa but not yet in Asia. With this IPT

strategy, pregnant women without symptoms of malaria attending routine antenatal care in selected health facilities in Papua, Indonesia, will receive monthly treatment with a long-acting antimalarial drug called dihydroartemisinin-piperaquine (DP) that provides four weeks of prophylaxis after each dose. Currently, women are screened for malaria at their first antenatal care visit, and women are treated with DP only if tested positive. In the new strategy, women will receive the drug as monthly prophylaxis without prior blood testing for malaria parasites.

A recent trial in Indonesia has shown that this intervention, when taken as directed, is very effective in preventing malaria in expectant mothers. However, the concept of using drugs for prevention by women who do not have malaria symptoms is new to this region. Indeed, this would be the first time that the IPT strategy would be used in South East Asia. The study is designed to find the best ways for the Ministry of Health to introduce and deliver this new intervention. It will use stepwise quality improvement approaches to understand what worked, for whom, and why during each step in the implementation phase to help improve the delivery of the new intervention and quality of service provision on an ongoing basis. Opportunities to bring the intervention close to the community through health posts where most women receive antenatal care will be strengthened. The study will also determine the costs and cost-effectiveness of the alternative new strategy, compared with the current strategy, to inform policy decision making for malaria prevention among pregnant women in Indonesia. It is anticipated that the study, by increasing the effectiveness of malaria prevention, has the potential to increase the provision and uptake of additional ANC services that prevent other causes of adverse birth outcomes in pregnancy. The final study results will be shared with the local offices of the Ministry of Health and the National Malaria and Reproductive Health departments in Indonesia to inform policy decision making for rolling out the strategy. The study will thereby contribute to improved outcomes for mothers and their infants in Indonesia, whose quality of life, health and creative output will be enhanced and will be of relevance to other parts of the Asia-Pacific region with similar malaria transmission patterns.

This will be a 32-month study, six months for study preparation, 22 months of fieldwork, data collection and data processing, and four months to conclude analysis and reporting.

3. Background and rationale

3.1. Malaria in pregnancy

Malaria infection during pregnancy has devastating consequences, causing severe maternal anaemia, pregnancy loss, intrauterine growth retardation, preterm delivery and increased infant morbidity and mortality. Annually, 88.2 (70%) of the 125.2 million pregnancies in malaria-endemic regions globally occur in the Asia-Pacific region, where *P. falciparum* and *P. vivax* are co-endemic. [1] Malaria in this region has been estimated to cause a 2.24 fold increase in the hazard of stillbirth and a 2.55-fold increase in the hazard of neonatal death mediated through small-for-gestational-age status and preterm birth. [2] Furthermore, *P. vivax* malaria itself increases the odds of stillbirth when detected at delivery (2·81 [0·77–10·22]), but not when detected and successfully treated during pregnancy (1·09 [0·76–1·57]), [3] suggesting that successful control of malaria in pregnancy in south-east Asia could have a major impact on pregnancy outcome.

Until recently, the prevention of malaria in pregnancy in the Asia-Pacific region relied on the provision of long-lasting insecticide-treated nets (LLINs) and passive case detection and management of febrile cases. [4] In 2012, Indonesia was the first country in the region to introduce

single screening and treatment (SST) in pregnancy. SST consists of screening women for malaria infections at their first antenatal care (ANC) visit with microscopy or rapid diagnostic tests (RDTs) and treating test-positive cases with artemisinin-based combination therapy (ACTs), followed by passive case detection. [5,6] Most countries in the region have since also adopted SST alongside LLINs and passive case detection. By contrast, in the Africa region, the World Health Organization (WHO) recommends intermittent preventive treatment in pregnancy (IPTp) consisting of curative doses of sulphadoxine-pyrimethamine (SP) given at every scheduled antenatal visit in the second and third trimester alongside LLINs and case management. This strategy has so far been unsuitable in Asia due to widespread resistance to SP, [7] the only antimalarial currently recommended by WHO for IPTp, [8] and the more modest levels of malaria transmission intensity. The only alternative drug that has been shown to be safe and effective for the intermittent treatment of malaria in pregnancy (IPTp) is dihydroartemisinin-piperaquine (DHP). Studies in Africa and Indonesia have shown that DHP is safe and effective in preventing malaria during pregnancy[9,13].

3.2. intermittent preventive treatment in pregnancy (IPTp)

In the first trial of its kind in South East Asia, we found that monthly IPTp with the artemisinin-based antimalarial combination dihydroartemisinin-piperaquine (DP) among pregnant women in the second and third trimester protected with LLINs was safe, tolerable, efficacious[9] and cost-effective under controlled clinical trial conditions. [10] The trial was conducted in Papua, Indonesia, an area with moderate *P. falciparum* and *P. vivax* transmission and high-level resistance to the antimalarials SP and chloroquine. IPTp-DP reduced the incidence of both *P. falciparum* or *P. vivax* infections by 77% and halved the risk of malaria infections at delivery compared to the standard SST strategy with rapid diagnostic tests (RDTs) and case management with DP. Results were consistent with the promising effects of IPTp-DP in three previous trials in Africa[11-13] which, in a meta-analysis, showed greater reductions in clinical malaria (73%), and malaria infection at delivery (75%) compared with SP and a lower risk of fetal loss (59-61%).[14,15] The nested economic study showed that IPTp-DP at a cost of USD 53 per DALY averted offers a cost-effective alternative to the current policy of SST-DP in the moderate malaria transmission setting of Papua, Indonesia, and compares favourably with the median cost per DALY averted by LLINs (USD 30), IPTp-SP (USD 27) and indoor residual spraying (USD 160). [16] Combined, these data suggest that monthly prophylaxis with IPTp-DP may provide a cost-effective alternative to the current policy in Papua Indonesia and other areas with moderate transmission and high-grade SP and chloroquine resistance in South East Asia.

Acceptability studies nested within this recent Indonesian trial showed that the existing SST strategy at antenatal booking was an acceptable strategy among both health providers and pregnant women. [5,6,17] However, providers had reservations about IPTp and giving antimalarials presumptively - *i.e.* without a confirmatory test - to asymptomatic women. This suggested that in this setting, a switch from the existing SST strategy, that tests all women at their first ANC visit, toward adoption of the proposed novel approach of monthly chemoprevention with IPTp-DP, would require a considerable shift in healthcare provider attitudes and behaviours. By contrast, pregnant women did not share these concerns and were prepared to take antimalarials presumptively as part of a package of antenatal care services they valued and trusted highly to protect themselves and their babies. [17] Evidence of its superior efficacy and the low sensitivity of the current generation of RDTs, especially for Plasmodium vivax, [18] may persuade healthcare providers to accept IPTp-DP as a potential strategy in the higher transmission areas in Indonesia

such as Papua. To achieve such a conceptual shift, the introduction of IPTp-DP in routine ANC services will require a robust evidence-based campaign of effective communication, training guidelines and job aids, and careful monitoring and supervision.

While clinical trials demonstrate clinical efficacy and safety of interventions, economic evaluations conducted alongside trials may not accurately represent cost-effectiveness under routine conditions. A key limitation is that acceptability and adherence among providers and users under trial conditions are likely to be higher than under routine health system settings. In addition, the costs of implementation under routine conditions may be different due to factors including scale, supervision/oversight and training. Hence, it is important to assess both the costs and effects under routine conditions to provide a more realistic indicator of cost and cost-effectiveness.

3.3. IPTp-DP pilot implementation in Papua

Based on the promising results, the MOH now intends to implement a pilot of IPTp-DP as a new strategy in Papua Province, where malaria is a major cause of maternal and neonatal death and have sought a collaboration with the study partners.

The National Expert Committee of Malaria Diagnosis and Treatment recommends that the Pilot Study could be carried out in malaria endemic area with the Annual Parasite Incidence (API) of >100/1000 populations. In addition, implementation of a novel strategy that requires an entirely new system and attitude in delivering antenatal care at village health posts would need to be carefully defined and evaluated to ensure sustainability. Pharmacovigilance system would also need to be reactivated. In view of this, the scope of the pilot study will be at district level which includes the NMCP staff, health staff involved in maternal care and pregnant women visiting village health posts for routine antenatal care. IPTp-DP will be provided presumptively for malaria prevention intervention for healthy pregnant women during routine ANC visits. In accordance with the recommendations of WHO in 2012, the provision of IPTp to pregnant women can be carried out by midwives/nurses. In malaria endemic areas in Africa, IPTp with sulfadoxine-pyrimethamine (SP) is administered by midwives during the ANC visit. The aim of preventative therapy for malaria is to maximise coverage and public health impact. Midwives have far greater access to pregnant women when they provide antenatal care services and also provide outreach services in the community.

At the start of the study, the MoH will be informed and consulted on the training plans (methods and materials) for IPTp implementation via virtual meetings in the first 3 months of training preparations. Subsequent meetings with the MoH will be held 6 monthly or as required to update and monitor the study progress. The study will be supervised and coordinated by the NMCP and Maternal Health Program team at the District Health Office (DHO). The research team would assist the DHO team in district wide program socialisation to the community to ensure support as well as developing, monitoring and evaluating the program implementation. During the course of the study, the Provincial Health Office and DHO will discuss options on whether they would return to implement SST or continue with IPTp when the pilot study ends.

The MoH expects that the research team could provide interim results at the end of the study (mid 2024) and present that to the National Expert Committee of Malaria Diagnosis and Treatment for their review and provide recommendation to the MoH by the end of 2024 or early 2025 to inform policy.

4. Impact and use of findings

MOH Indonesia has invited our research team to support the pilot implementation of the new malaria chemoprevention strategy, IPTp-DP, to prevent adverse pregnancy outcomes in Papua, Indonesia. This study will provide essential evidence for the most promising delivery strategies to rollout this new strategy. Understanding the bottlenecks in service delivery and health providers' experiences of prescribing IPTp with DP to pregnant women can contribute to improving the quality of patient care (prescribing, administration, counselling) and maximise therapeutic outcomes. This information can also guide strategies to improve patient adherence to IPTp with DP, enhancing outcomes due to malaria in pregnancy. The study is also designed to inform the strategy for scale-up.

The addition of quality improvement (QI) approaches during the first year of the pilot will identify what works, for whom, and why, providing actionable evidence to optimise roll out and strengthen the quality of service delivery. Costing analyses will provide essential information related to financing requirements and implications for the health budget i.e. change in resource use.

Results will be used directly by the government to guide decision making on whether to roll out the strategy across the entire Papua Province. Results will also be discussed at a stakeholders' meeting involving National, Provincial and District Health Offices, partners and local communities. Based on emerging consensus, policy guidelines will be prepared and disseminated. It is anticipated that policy impact will occur within 6-12 months after study completion and will result in increased effectiveness of malaria prevention, and potentially also increased uptake of other ANC services, leading to improved outcomes for mothers and their infants in Indonesia, whose quality of life, health and creative output will be enhanced. The findings will also be of substantial interest to other countries in the region and Asia-Pacific region and will be disseminated through the Asia Pacific Malaria Elimination Initiative (APMEN) as we did for the original trial and nested studies.

5. Aims and objectives

5.1. Overall Objective

To explore the programme effectiveness and scalability of monthly doses of dihydroartemisinin-piperaquine preventive treatment (IPTp-DP) delivered by the MOH pilot programme to prevent malaria in pregnancy and improve birth outcomes in Papua, Indonesia.

5.2. Specific Objectives

1. To assess the programme effectiveness of IPTp-DP delivered through routine health system at the end of the pilot and assess health provider perceptions of the drivers of successful integration and scalability to inform potential rollout.

2. To assess adherence among pregnant women to the full IPTp-DP regimen and their perceptions of the strategy at the end of the pilot to refine strategies to improve uptake.
3. To develop embedded quality improvement procedures to maximise IPTp-DP service delivery and uptake to understand what worked, for whom, and why.
4. To generate routine pharmacovigilance data on the safety of DP when used monthly for IPTp during pregnancy in the Indonesian population.
5. To estimate the net cost-effectiveness of implementing IPTp-DP and the net cost-effectiveness of the current policy of the single screen and treat strategy in the routine health system and compare the cost-effectiveness of these alternative options. We will also estimate the incremental financial costs of implementing IPTp-DP (including associated pharmacovigilance and other necessary health systems strengthening and quality improvement initiatives) from the provider perspective at scale in Papua, Indonesia.

6. Overview of study design

This will be a mixed method process evaluation in a ‘real life’ setting of the IPTp-DP pilot programme led by the Indonesian Ministry of Health (MOH) in Papua Province. In the first 18 months, the study team will support the district and provincial health departments of MOH to implement the intervention using embedded quality improvement (QI) approaches to continuously strengthen service delivery, uptake and adherence through a series of ‘plan-do-study-act’ cycles. The evaluation design is predicated upon several assumptions about conditions and contextual factors in the service delivery, household, and broader social environments that will lead to IPTp-DP adoption and adherence. These assumptions are made explicit in the IPTp-DP uptake logic model shown in Figure 1. The model represents the study’s initial overarching conceptual framework to structure data collection and analysis procedures while allowing for inductive discovery through open-ended inquiry; the model will be reviewed and revised in consultation with local MOH and other stakeholders prior to the implementation of the intervention.

A mixed-methods evaluation will be conducted in the last six months of the IPTp-DP pilot using exit interviews to assess service delivery effectiveness, home visits within 4-5 days of their clinic visit to assess adherence to the 3-day DP treatment regimen, and qualitative research to explore provider perceptions of the drivers of successful integration, scalability and user acceptability.

The primary outcome is adherence, defined as the proportion of pregnant women who receive the first dose of IPTp-DP by directly observed therapy (DOT) at the antenatal clinic (ANC) and have the correct number of DP tablets for subsequent doses on exit (delivery effectiveness), and when visited at home have verified they completed the course. We will also support the MOH to collect safety data for pharmacovigilance.

A nested health economic study will determine the cost-effectiveness of IPTp-DP compared to the current policy of single screening and treatment (SST) under routine conditions, and the incremental cost of implementing IPTp-DP at scale in a way that will support the MOH strategy to improve neonatal and maternal outcomes.

Figure 1. Logic model for IPTp delivery and uptake

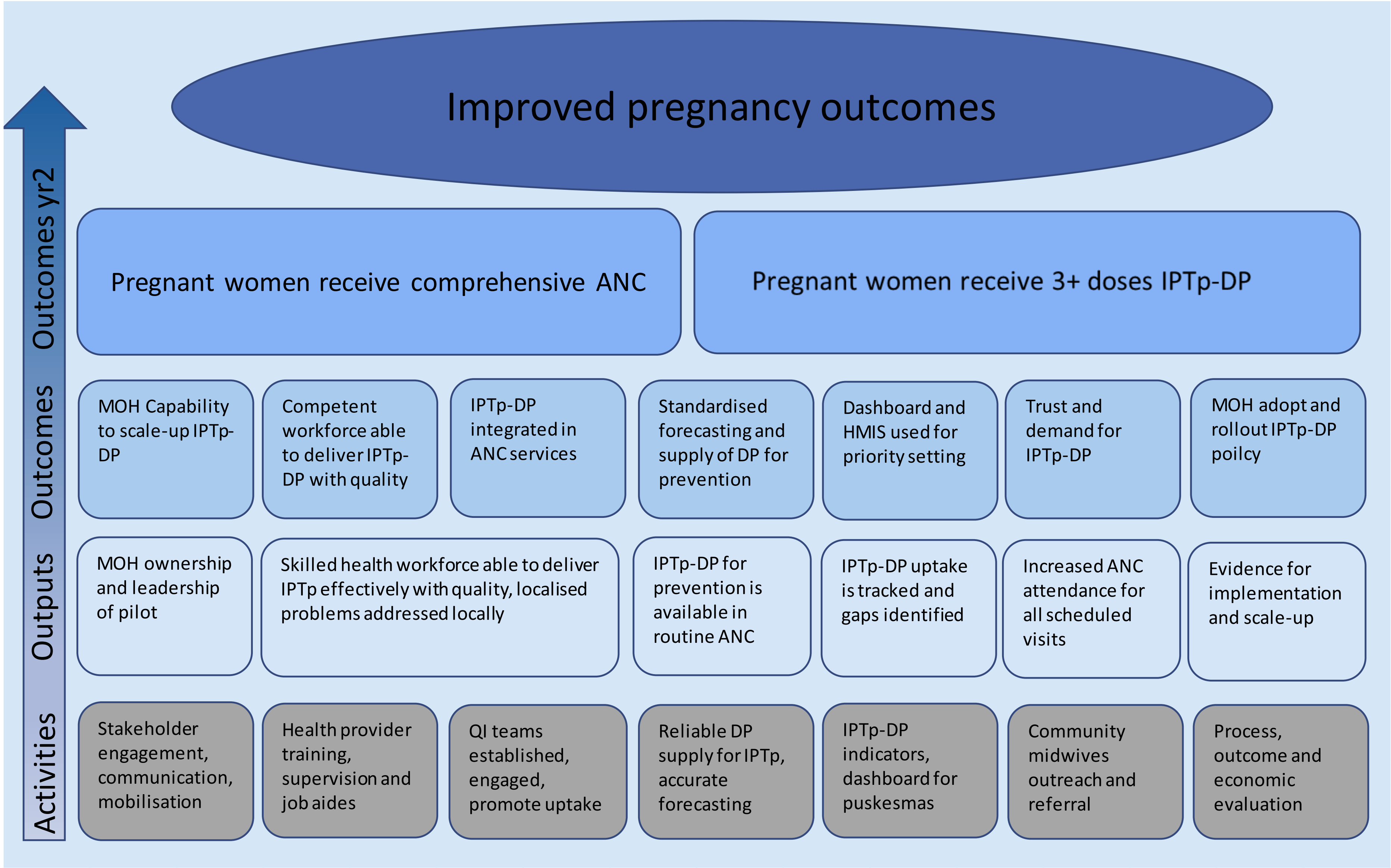
Improved pregnancy outcomes
Outcomes yr2
Outcomes
Outputs
Activities
Pregnant women receive comprehensive ANC
Pregnant women receive 3+ doses IPTp-DP
MOH Capability to scale-up IPTp-DP
Competent workforce able to deliver IPTp-DP with quality
IPTp-DP integrated in ANC services
Standardised forecasting and supply of DP for prevention
Dashboard and HMIS used for priority setting
Trust and demand for IPTp-DP
MOH adopt and rollout IPTp-DP poilcy
MOH ownership and leadership of pilot
Skilled health workforce able to deliver IPTp effectively with quality, localised problems addressed locally
IPTp-DP for prevention is available in routine ANC
IPTp-DP uptake is tracked and gaps identified
Increased ANC attendance for all scheduled visits
Evidence for implementation and scale-up
Stakeholder engagement, communication, mobilisation
Health provider training, supervision and job aides
QI teams established, engaged, promote uptake
Reliable DP supply for IPTp, accurate forecasting
IPTp-DP indicators, dashboard for puskesmas
Community midwives outreach and referral
Process, outcome and economic evaluation

7. Methods

7.1. Study site

The study will be conducted in Mimika District in the south of Papua Province, Eastern Indonesia, covering 19,952 square kilometres (sq km) consisting of highlands and lowlands. Mimika has a rapidly growing population of an estimated 210,407 (2017 census), with the main occupations in commerce, business, and retail. The Annual Parasite Incidence in this region in 2018 is 250 per 100,000 population at risk with a population of 215,541 (Mimika District Health Office 2018-Malaria Report). The majority of malaria cases are in the lowlands. The prevalence of parasitaemia among pregnant women at delivery is 16.8% (58% P. falciparum infections, 34% P. vivax, and 8% mixed infections), with 35% of these infections being associated with fever. [19]

The main health facilities which deliver antenatal care (ANC) services to pregnant women are hospitals, community health centres (known in Indonesian as *puskesmas*) covering about 30,000 people, sub-health centres (*pustu*), which serve about 2-3 villages and 2000-3000 population, and health posts (*posyandus*) or community integrated services including antenatal care, which are held monthly or bi-monthly in villages. In Mimika, almost half of women (46%) made at least four antenatal care visits, and the majority (97%) of women attended ANC at least once in health facilities (Mimika District Health Office 2019). There are two referral hospitals in the district that provide emergency obstetric and neonatal care.

The IPTp-DP intervention will be delivered in 10 of 17 purposively selected (accessible) community health centres and their associated health posts in the lowlands in Timika city. The District Health Office (DHO) will be responsible for all aspects of IPTp-DP delivery through antenatal clinics in terms of human and institutional resources and infrastructure as part of routine antenatal care services. Senior DHO staff supported by the Timika Research Facility will be responsible for training DHO staff, procuring and providing DP for use as IPTp for the duration of the study and providing technical support. DHO nursing staff providing routine antenatal care services in *puskesmas*, *pustu* and *posyandu* will be trained to deliver IPTp-DP to pregnant women attending ANC in their second or third trimester. Delivery of IPTp-DP by health staff at the community level (village health post midwives) to extend reach and promote adherence will be essential to the IPTp-DP strategy.

7.2. Description of the intervention

The pilot will be implemented over 16 months (Q1 2022-Q2 2023).

The Mimika District Health Management Team will be responsible for the training and supervision of antenatal staff who will be delivering IPTp-DP in the selected health facilities (*puskesmas* and *posyandus*) with support from the study team. Currently, nurses are not permitted to prescribe antimalarials, and they will need to be trained and supported to provide IPTp-DP in puskesmas and the community. Training will include administration of IPTp-DP, eligibility for receiving IPTp, safety monitoring, management of side effects, stock control and

key messages for counselling pregnant women on adherence and what to do in the case of vomiting or other side effects. Guidelines and materials on IPTp-DP will be provided by the study team.

The Provincial Health Office (PHO) will provide sufficient DP tablets for IPTp in the study population for the duration of the pilot using the existing PHO drug supply and distribution systems that supplies drugs for routine antenatal care services.

Pregnant women attending routine ANC visits at puskesmas and posyandu in their second and third trimester will be given a presumptive treatment dose of DP daily for three days by village midwives and nurses working in routine ANC. The first dose will be given by directly observed therapy (DOT), and the remaining doses given in blister packs to the women to take at home on day 1 and day 2. Additional IPTp-DP courses will be given at each subsequent ANC visit no less than one month apart. All pregnant women will receive the standard treatment dose of 3 DP tablets per day for 3 days (9 tablets). Pregnant women who refuse IPTp-DP, who are contraindicated e.g. taking daily CTX, and who visit ANC in their first trimester will be given SST (standard of care).

7.3. Study populations

The study population will consist of healthcare providers working in antenatal clinics at health centres (*puskesmas*) and health posts (*posyandu*), health managers at various levels of the system, community leaders, pregnant women, and husbands.

7.3.1. Inclusion criteria

7.3.1.1. Health facilities

- Antenatal services must be operational and accessible
- Midwives/nurses have been trained to prescribe IPTp-DP

7.3.1.2. Healthcare providers (QI initiative and in-depth interviews)

- Healthcare providers responsible for providing antenatal care services, and facility managers
- District and provincial health managers

7.3.1.3. Community leaders (focus group discussion)

- Community leaders aged 18 years or more
- Community leaders who are recognised by community members and have held a leadership position for at least 6 months.
- Community leaders who are residing within the catchment areas of the health facilities participating in the pilot

- Community leaders who have been exposed to IPTp-DP information

7.3.1.4. Pregnant women (health facility exit interviews; home visits; in-depth interviews)

- Pregnant women aged 15-49 years
- Women in 2nd/3rd trimester of pregnancy
- HIV negative (where status is known)

7.3.1.5. Husbands of pregnant women (in-depth interviews)

- Husbands of pregnant women aged 18 years or more
- Husbands of pregnant women who are in the 2nd/ 3rd trimester of pregnancy.
- Husbands who are residing within the catchment areas of the health facilities participating in the pilot.

7.3.1.6. Community health workers (in-depth interviews)

- Community health workers aged 18 years or more
- Community health workers who have been involved to IPTp-DP activities

7.3.2. Exclusion criteria

7.3.2.1. Health facilities (Pilot implementation)

We selected 10 from 17 health facilities in Papua based on accessibility and functionality to be the pilot study sites. The remaining health facilities have been excluded due to accessibility issues.

7.3.2.2. Health facilities (Data collection for evaluation)

- Health facilities not enrolled in the pilot will be excluded.

7.3.2.3. Healthcare providers (QI initiative and in-depth interviews)

- Health workers providing ANC and IPTp-DP services in Puskesmas who have provided services for less than 1 month.

7.3.2.4. Community leaders (focus group discussion)

- Community leaders who are unable to communicate effectively in Indonesian.

7.3.2.5. Pregnant women (health facility exit interviews; home visits; in-depth interviews)

- Women with communication or language problems including not being able to speak Indonesian.
- Pregnant women are unwell during the interview
- Pregnant women who move outside the pilot implementation areas.

7.3.2.6. Husbands of pregnant women (in-depth interviews)

- Husbands who are unable to communicate effectively in Indonesian.

7.3.2.7. Community health workers (in-depth interviews)

- Community health workers who are unable to communicate effectively in Indonesian.

7.4 DELIVERY effectiveness and adherence (objectives 1 & 2)

7.4.1. Outcomes

Delivery effectiveness will be evaluated using: 1) a cross-sectional survey of delivery effectiveness of IPTp-DP at ANC assessed through exit interviews with pregnant women, and 2) longitudinal follow-up of adherence to DP as a multi-day regimen (IPTp-DP) assessed through home visits.

Primary Outcome: Adherence to 3-day IPTp regimen

The proportion of pregnant women who both receive the first dose of IPTp-DP by DOT at ANC and have the correct number of DP tablets for subsequent doses on exit (i.e. secondary outcome), and when visited at home have verified they completed the treatment (adherence). Women who receive the correct dose of IPTp-DP as assessed in the exit interviews will then be followed up at home on day 4 or 5 after their clinic visit (i.e. one to two days after the third dose of 3-day DP) and interviewed about adherence (self-report), and pill counts performed.

Secondary outcome: Delivery effectiveness

Delivery effectiveness will be assessed through a cross-sectional survey of health facilities enrolled in the pilot, using unstructured observations during antenatal care and exit interviews with pregnant women leaving antenatal clinics. The endpoint will be 'the proportion of women attending ANC treated appropriately according to the IPTp-DP guidelines', defined as the first dose given by directly observed therapy (DOT) plus adequate doses to take home for days 1 and 2. Their understanding of the treatment regimens given during that ANC visit will also be assessed. A health facility audit, together with unstructured observations, will be used to provide context to the findings of the exit interviews.

Definitions

An appropriate dose of IPTp DP is defined as a first dose reported as given with DOT and leaving the facility with the correct number of tablets for subsequent doses.

Verified completed treatment is reported completed treatment corroborated by a pill count.

Verified timely completed treatment is reported completed treatment corroborated by a pill count where the pills are taken at the time required.

7.4.2. Sample selection

Systems effectiveness

All 10 health facilities in the MOH pilot will be included in the evaluation. Pregnant women will be randomly selected for exit interviews at ANC registration by lottery, which will take into account the number of women expected at the health facility that day and the number of field workers available to conduct the exit interviews.

Adherence to DP

All health facilities in the MOH pilot will be included in the evaluation. Pregnant women will be randomly selected from a list of women who received a full dose of IPTp with DP on exit using random number tables and followed-up at home within 4 to 5 days after their visit, i.e. no more than 2 days after their 3-day regimen is due to finish. If the visit cannot be completed, the interviewer will try again the following day. No interviews will be carried out beyond 5 days from the ANC visit.

7.4.3. Sample size

A sample size of 1080 women from ten clinics (108 per clinic) will allow the detection of an estimated 60% of women achieving the primary endpoint[20] with a precision of +/-6%, 95% CI, and a design effect (DE) of 4.22 based on an Intra-Cluster Correlation Coefficient (ICC) of 0.03. The ICC of 0.03 was selected based on earlier studies which showed ICC values of 0.02-0.03 for similar outcomes. [21,22] The sample size is comparable with other similar studies. [23]

We expect to enrol a total of 1,440 women studied for the secondary endpoint of systems effectiveness. Allowing for an expected systems effectiveness of 75%, there will be 1080 available for analysis of the primary endpoint of adherence rate.

Justification for sample size

This study will primarily estimate the adherence and delivery effectiveness rate of IPTp-DP in the real-world setting. Based on our estimate for an adherence and delivery effectiveness rate of 60%, the sample size is comparable with other similar studies. [23] A confidence level of 95% and precision of 6% was selected by considering both available financial resources and acceptable clinical relevance. This gave a sample size without adjusting for cluster effects of 256.

The Design Effect (DE) for clustering was estimated based on the variability in clinic-level delivery rate. It is believed that there will be more variability in the results for delivery than for adherence, that delivery rate is an important secondary endpoint, and doing this provides a conservative value for the DE for patient-level adherence.

The number of patients needed per cluster was estimated based on there being 10 clusters, an Intra-Cluster Correlation (ICC) of 0.03, and the following formula:

$m = (N(1-ICC)) / (k – (ICC*N))$

Where m is total patients per cluster, N the unadjusted sample size, and k the number of clusters per arm. The number of patients per cluster needed was 108, or 1080 overall, for a DE of 4.22.

The ICC was a conservative estimate based on the ICC for treatment delivery rate from previous similar cluster-randomised studies. [21,22]

7.4.4. Data collection procedures

Delivery effectiveness

A structured questionnaire will be used as the selected pregnant women exit ANC (**Appendix 1**). Topic categories within the questionnaire will include demographics, current and past pregnancy history, reason for attendance on the day of the interview, details of IPTp received, knowledge on how to take remaining IPTp doses where applicable, and any other medications received.

Adherence to DP

A structured questionnaire will be used at follow-up home visits to the selected pregnant women (**Appendix 2**). Topic categories within the questionnaire will include demographics, current and past pregnancy history, details of the IPTp regimen (number of doses taken, number of tablets taken, timing of doses), taken with/without food. Additional questions will be asked about any side effects experienced, and any problems that led women to not completing the full course. Pill counts will be conducted.

7.4.5. Consenting procedures

Exit interviews: We will obtain informed consent from participants as pregnant women arrive and register for their ANC visit, before they enter the clinic. Women who provide consent will then be interviewed after they conclude their ANC visit. During the consenting process, individuals will be informed that they can ask questions about the study and the process at any time in advance of or during the interview. On exiting ANC, participants will be reminded that they are free to decide whether or not to withdraw their consent depending on the time taken for them to complete their ANC visit and resultant availability.

Home visits: We will obtain informed consent from participants at the time of the interview to avoid influencing adherence behaviours by gaining consent prior to the home visit. Participants will be given an opportunity to decline participating in the interview and be reminded that they can stop the interview at any time.

7.5. Scalability (Objectives 1 & 2)

7.5.1. Sample size and selection

In-depth interviews at mid-term and end-term – Health providers: Health providers in antenatal care clinics in the pilot health centres and health posts will be selected through purposive sampling and include the head of the facility (2-3 per health facility depending on size, n=approx. 60). Interviews with purposively selected district and provincial managers (n=approx. 5).

In-depth interviews at mid-term and end-term – Pregnant women: 3-5 pregnant women per health facility (n=approx. 100); at least 1 primigravidae, 2 multigravidae, and a mix of first vs subsequent visits during this pregnancy.

Focus group discussion at end-term - Community Leaders: Community leaders will be selected through purposive sampling. Community leaders, include representatives from religious leaders, tribal leaders, and kinship leaders (n=approx. 10).

In-depth interviews at end-term - Husbands of Pregnant Women: 2-3 husbands per health facility will be selected through purposive sampling (n=approx. 25). The selection will consider a mix of first-time fathers and those with previous children, to gain insights into different levels of experience and perspectives on supporting their wives through pregnancy and malaria prevention. The interviews with husbands will be conducted only at the end-term to understand their perspective on the pilot program and to evaluate the support systems for pregnant women.

In-depth interviews at end-term - Community Health Workers: 1-2 community health workers will be selected through purposive sampling. The selection will consider a mix of new and experienced community health workers to gain insights into different levels of experience and perspectives on implementing the pilot program and supporting pregnant women (n=approx. 10). The interviews with community health workers will be conducted both at end-term to understand their perspective on the pilot program and to evaluate the effectiveness of the program from their point of view.

7.5.2. Data collection procedures

In-depth interviews will be used to explore the acceptability and scalability of IPTp-DP among health providers and pregnant women compared to the current policy of SST. Qualitative data will be used to explain primary and secondary outcomes.

In-depth interviews - Health Providers

Health provider perceptions of giving antimalarials as a preventive treatment in the absence of a confirmed diagnosis will be explored as this strategy is new to Indonesia. In-depth interviews with health providers in antenatal care clinics in the pilot health centres and health posts and the head of the health facility will assess: 1) acceptability of IPTp-DP, 2) the adaptations to their working practices that were required to implement IPTp-DP, 3) their perceptions of the feasibility of implementing the intervention at scale including any resource constraints, and 4) their recommendations on factors to be considered to ensure effective implementation (**Appendix 3**). Interviews will be conducted at mid-term and end-term to capture changes in perceptions of the providers following the two QI cycles.

In-depth interviews will also be conducted with purposively selected district and provincial managers and invited to share their views on progress of the pilot program, changes that have brought about improved provider or user adherence, and the feasibility of scale up (**Appendix 4**).

Focus group discussion– Community Leaders

Engaging community leaders including tribe leaders, and religious leaders are essential to gather a comprehensive understanding of the cultural, and social aspects that may influence the acceptability and implementation of IPTp-DP in Papua, Indonesia. In-depth interviews with community leaders will focus on assessing their understanding and perception of malaria prevention during pregnancy, and the acceptance of IPTp-DP within their communities. The interviews will also explore the role that community leaders can play in promoting IPTp-DP, and the support needed from the health system to facilitate this. Additionally, their insights on cultural practices, community beliefs, and barriers to access that could affect the uptake of IPTp-DP among pregnant women will be sought. Community leaders' recommendations on community engagement strategies, communication, and education initiatives to improve the acceptability and adherence to IPTp-DP will also be collected. These interviews will be conducted at the end-term of the project to understand the community dynamics and explore opportunities for scaling the intervention.

In-depth interviews - Pregnant Women

In-depth interviews with pregnant women will explore the acceptability of IPTp-DP compared to SST, their perceptions of being given a preventive treatment in the absence of a parasitological test, and any challenges they face in access/uptake/completion (**Appendix 5**). Interviews will be conducted at project mid-term and end-term to explore changes in perceptions over the course of the pilot.

In-depth interviews – Husbands of Pregnant Women

Involving the husbands of pregnant women in the evaluation process is crucial for understanding the family dynamics and support systems that may impact the acceptance and adherence to IPTp-DP. In-depth interviews with husbands will delve into their knowledge and attitudes towards malaria prevention during pregnancy, their perceptions of IPTp-DP as a preventive treatment, and their willingness to support their wives in adhering to the regimen. The interviews will also assess the husbands' concerns, if any, regarding the safety and efficacy of IPTp-DP, and explore their ideas for overcoming barriers to adherence. Moreover, their views on the role that they can play in disseminating information about IPTp-DP within their social circles and contributing to the overall success of the program will be explored. Understanding the husbands' perspective is important, as family support is often a key factor in healthcare decisions, especially in settings where cultural norms might dictate shared decision-making in households. These interviews will be conducted at project end-term.

In-depth interviews - Community Health Workers

Community health workers play a crucial role in implementing health programs at the community level. In-depth interviews with community health workers will explore their experiences and challenges in implementing the IPTp-DP program. This includes their perceptions of the acceptability of IPTp-DP among the community, the adaptations they had to make in their work practices to implement IPTp-DP, and their views on the feasibility of scaling

up the intervention. The interviews will also delve into their understanding of the community dynamics that may affect the uptake and adherence of IPTp-DP and their recommendations for improving the program's implementation and acceptance. These interviews will be conducted at end-term to capture their perceptions and experiences over the course of the pilot.

7.5.3. Consenting procedures

In-depth interviews with health providers: Permission to interview healthcare staff will first be sought from the head or officer in-charge of the participating health facilities, prior to enrolment of health providers. Research staff will explain to the head of the facility that participation is entirely voluntary and that the decision to participate or not should have no bearing on their job. Consenting for one-on-one interviews will be conducted in a private setting by research staff conducting the interview. The research staff will explain that permission to conduct the study in that facility has been obtained from the head of the health facility, that their participation is entirely voluntary and that the head has stated that the decision to participate or not should have no bearing on their job. They will be assured that all information from interviews with health providers will be kept confidential.

In-depth interviews with pregnant women: Consenting will be conducted in a private setting by trained research staff who will conduct the interview. Participants will be given an opportunity to decline their participation before the interview commences and they will be reminded that they can withdraw at any time in the process.

In-depth interviews with husbands of pregnant women

Husbands of pregnant women will be identified and recruited through a community-based approach. This approach will allow gathering a representative view of the socio-cultural practices in the areas around the 10 study facilities. The research team will collaborate with village midwives and local chiefs, who are well-acquainted with their communities, to assist in identifying a range of husbands range from diverse socio-cultural backgrounds. These community figures will be the entry point for contacting potential participants; they will explain the purpose of the study and invite the husbands to participate, emphasising that their participation is voluntary and that they can withdraw at any time without any consequences. Only once a husband has expressed interest in participating in the study and has given verbal consent to be contacted by the study team, will the village midwives or local chiefs provide the study team with his contact information. The study team will contact the husband to provide more detailed information about the study, answer any questions they may have, and schedule the interviews. Consenting will be conducted in a private setting by trained research staff who will conduct the interview. Participants will be given an opportunity to decline their participation before the interview commences and they will be reminded that they can withdraw at any time in the process.

Figure 2. Study design

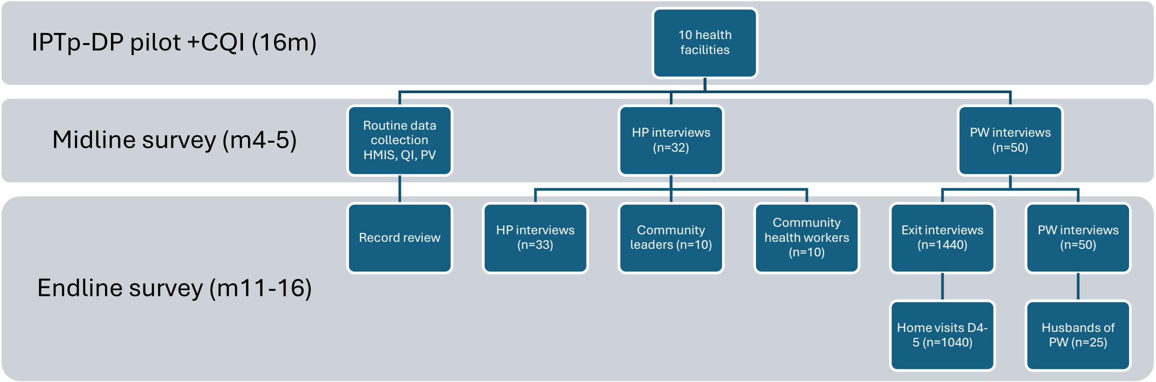

7.6. QUALITY IMPROVEMENT (OBJECTIVE 3)

7.6.1. QI Process

The continuous quality improvement (CQI) initiative will begin after the first 3 months of implementation with the establishment of work improvement teams from ANC staff and their line managers at each puskesmas who will receive a 3-day training workshop. The workshop will cover the core concepts of quality assurance and quality improvement and its application to the context of ANC services; data quality and how to record disaggregated data at puskesmas and community levels and report to the national health information system; and development of a strategy to capture client experience and satisfaction with the delivery of ANC services. The teams will be invited to a second workshop after 3 further months of implementation to review data and identify locally identified challenges, prioritise and define problems; root cause analysis; and develop a list of potential interventions to improve service quality. [24] This will be followed by one complete 'plan-do-study-act' (PDSA) cycle to 'test and implement', with a 3 month gap between each step in the cycle so that the cycle is completed by month 14 (Figure 2). The teams will be supported by coaches from the sub-District Health Office supported by the study implementation team. At the end of the cycle a third workshop will be held to present and evaluate their progress in implementing their QI Change Plans through interactive methods enabling reflection and evaluation involving managers, supervisors, providers and community members. The three workshops will be facilitated by an experienced study staff in the implementation study team, who will also serve as coaches.

7.6.2. Data collection procedures

Key performance indicators will be used to monitor the impact of the QI interventions on IPTp-DP delivery and uptake, such as the delivery of the first DP dose by DOT and the proportion of ANC attendees in the second and third trimester receiving IPTp-DP by gestational age. Performance and coverage indicators will be captured in revisions to antenatal clinic registers and reported through the routine health management system (HMIS). The SQUALE toolkit tools will be adapted for use in the IPTp-DP pilot, including procedures for data quality assessment of

routine data http://usaidsqale.reachoutconsortium.org/publications-and-tools/usaid-sqale-toolkit/

Figure 2. SQALE cascade http://usaidsqale.reachoutconsortium.org/

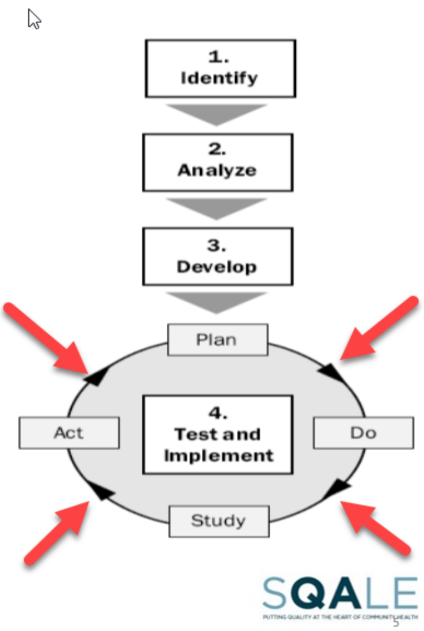

7.7. Process evaluation

The process evaluation[25] will seek to explain how and why such impacts, did or did not, come about and for whom - how the implementation of the pilot programme happened, whether hypothesised causal pathways were activated and identify contextual factors that serve as barriers or facilitators to either implementation, effectiveness, or both. The initial logic model (Figure 1) will be refined at the start of the study with all stakeholders involved in intervention design and implementation.

Based on the final logic model, we will use mixed methods - quantitative methods to measure key process variables and allow testing of pre-hypothesised mechanisms of impact and contextual moderators, and qualitative methods to capture emerging changes in implementation, experiences of the intervention and unanticipated or complex causal pathways, and to generate new theory. Data on key process variables will be collected from all sites and more detailed qualitative data from smaller, purposively selected participants. Qualitative data will be collected at mid-term and end-term to capture changes to the intervention over time. Table 2 summarises the key components of the process evaluation.

Table 2. Key components of the process evaluation[25]

Implementation process	Methods	Indicators	Data source	Timepoint
**Context**				
Pilot (other facilities get SST)	Stakeholder interviews; documentary analysis; unstructured observations	N/A	IDIs	mid-term; end-term
COVID (pandemic, vaccine)	Stakeholder interviews; documentary analysis; unstructured observations	N/A	IDIs	mid-term; end-term
Other	Stakeholder interviews; documentary analysis; unstructured observations	N/A	IDIs	mid-term; end-term
**Implementation processes**				
Training	Record review	Proportion of ANC staff trained to deliver IPTp-DP	Study records; MOH records	throughout
Resources	Human, material, other	ANC staff to client ratio	Health facility records	throughout
Costs	Cost data	Health facility costing exercise	CRF	throughout
Supervision	Record review	Proportion of midwives supervised by DHO at least x times each quarter	MOH checklists	throughout
Drug supply	Record review	Stock outs (no. weeks) during pilot	MOH store records	throughout
QI adaptations	Stakeholder interviews; documentary analysis; unstructured observations	No. and impact of QI interventions [see performance indicators] e.g. improve data quality, IEC activities, OTJ training	QI tools	throughout
Perfomance indicators	Record review	Proportion ANC attendees in 2nd/3rd trimester receiving IPTp-DP; proportion receiving 1st dose by DOT	HMIS records	throughout
**Outcomes**				
Fidelity	Exit interview (delivery effectivess)	Proportion ANC attendees treated according guidelines (first dose by DOT plus adequate doses for day 1 + day 2)	CRF	end-term
Reach (target group receiving IPTp-DP with quality i.e. coverage and fidelity)	Population based survey	Proportion of eligible population with 3+ doses	Next DHS?	end-term
Adherence	Home visit	Proportion ANC attendees receiving IPTp-DP per guidelines on exit who verified treatment completed at home	CRF	end-term
Acceptability (HPs and PW)	IDIs	N/A	IDIs	end-term
**Mechanisms of impact**				
	Stakeholder interviews; routine data	N/A	IDIs	mid-term; end-term

7.8. Pharmacovigilance (objective 4)

Safety monitoring will be included as part of health system capacity strengthening for routine pharmacovigilance in pregnancy. The study site benefits from the capacity and tools that were recently (2017-2019) developed to prospectively capture pregnancy outcomes and congenital malformations as part of an Indonesian study of pharmacovigilance of DP use in pregnancy funded by the Medicines for Malaria Venture (MMV). All safety data will be provided into a new pregnancy exposure registry that is currently under development by MMV and LSTM. A team of clinicians consisting of an internal medicine specialist, obstetrician and paediatrician will be

established in the two hospitals to review SAEs and assess the causality with DP as preventive treatment. The Indonesian PI will assist the documentation (**CRF 6**) and facilitate the process. Piperaquine in the fixed-dose DP combination is known to be associated with QTc prolongation but the pro-arrhythmic potential of piperaquine to cause clinically relevant arrhythmias or torsade des pointes appears very low and similar to the background rate. [26] Nevertheless, to collect further safety data, special attention will be placed on potential cardiac events, defined as syncope (fainting) or seizures occurring within 24 hours after DP intake. If such an event occurs, cardiac evaluation, including ECGs and appropriate treatment is given.

7.9. Economic studies (objective 5)

We will conduct activity-based costing, using the ingredients approach combined with step-down and micro-costing to estimate the costs by health system level (provincial, district and health facility, including associated health post) of delivering IPTp-DP versus the cost of delivering SST. First, we will identify the activities to be costed for both IPTp-DP and, separately, SST (e.g. staff training and supervision, IPTp DP or SST administration, supply/drug chain management, safety monitoring, communication with stakeholders, counselling women etc.) by programme phase (planning implementation pilot, QI round, evaluation). Activities will be defined as either implementation or research with research costs excluded from the analysis. Bottom-up, i.e. facility-level cost data will be collected in two IPTp-DP facilities and two SST facilities, matched on facility size (staffing quotient and the number of health posts) and population served (size and demographic/socioeconomic status). This will be combined with information on staffing levels and other relevant indicators (e.g. difficulty of access) and used to estimate costs in the remaining facilities. Health system costs (facility running costs, staff costs and equipment) will be allocated to the relevant intervention using step-down costing to calculate a unit cost per woman receiving each intervention in Papua Province. Total cost and cost per woman will be presented and analysed by health system level and by cost type (e.g. staff costs, equipment, drugs, consumables) to identify the key cost drivers.

Patient costs: The main (base case) analysis will be conducted from the provider perspective. We will also collect information on the cost of IPTp-DP incurred by pregnant women during the exit interviews and home visits. It will not be possible to compare these costs directly to those incurred for SST as no local data exists from previous studies. However, as we anticipate that the majority of costs faced by women will be travel time and costs to attend clinics, we will estimate the cost difference on this basis.

Effects: Exit interview and home visit information will be used to estimate the proportion of women receiving and completing IPTp-DP, whereas data from previous studies, supplemented by facility records, will be used to estimate the proportion of women accessing and adhering to SST. Health effects of each intervention will be estimated using data from our recent clinical trial[9] and converted into DALYs using standard weights and methods.

We will estimate the total, unit, incremental health care cost and utilisation of the IPTp-DP versus SST. Cost-effectiveness analysis also will be conducted from the provider and societal perspectives using a lifetime horizon to capture the possible long-term impact of IPTp-DP. To support policy decision making for wider implementation beyond Mimika District to the whole of Papua, health facility costing and data on the Papua health system (e.g. facilities by province) will be used to estimate the cost of scaling up the intervention at the regional/national level.

8. Data Management

8.1. Data Storage

8.1.1. Quantitative data

Data collected from the exit interviews and home visits will be collected on tablets. The data entry program will have in-built range checks and skip-patterns to minimise errors.

Data from the health facility costing will be collated in an Excel spreadsheet with in-built pivot tables to display key results. The data in the spreadsheet will be checked for accuracy by a second investigator. The health facility costing does not collect any confidential data and therefore does not require any further data storage steps but will be kept electronically in data repositories for the purpose of avoiding potential data loss caused by technical or human error. All data will be kept in a single Excel Workbook, which will be password protected and contain automatic date stamps to indicate who last accessed it. Only the Health Economists and Study PI/Co-Is will have access. Version control will be implemented to ensure data integrity. The workbook versions will be stored on a cloud-based file-sharing system to facilitate access to the same version for UK and Indonesian based collaborators.

8.1.2. Qualitative data

Audio-files will be named and numbered following an agreed-upon file naming convention and archived by the study team. At the end of each interview day, audio-files and quantitative data will be uploaded and saved to study computers with backup copies saved and stored on the TRF server on a drive only accessible to the study team. Data on the TRF server is backed up daily, and for additional backup, data will be stored on LSTM’s OneDrive for Business. TRF and LSTM have standard operating procedures for secure data storage and archiving. The qualitative data collected in this study will be in the form of open text and will include field notes, reflective notes, and the transcripts of recordings of interviews. Digital recordings, interview transcripts and field notes will be securely stored in password-protected computers. No names or other easy identifiers will appear on the transcripts, and the identification codes for the transcripts will be kept in a separate secure location. All digital recordings will be deleted at the end of the study. All participants mentioned in field notebooks will be anonymised. Field notebooks, ICFs and any other study documents containing identifiable personal information will be stored in locked cabinets. These documents will be destroyed at the end of the study in line with TRF guidelines and policies.

Data will be archived and stored in a Good Clinical Practice (GCP) compliant storage area. The data collected during the study will be transferred to researchers at the Liverpool School of Tropical Medicine in the UK for analysis. Your data will be handled in accordance with UK Data Protection Act 2018. An Act to make provision for the regulation of the processing of information relating to individuals; to make provision in connection with the Information Commissioner's functions under certain regulations relating to information; to make provision for a direct marketing code of practice; and for connected purposes.

8.2. Data processing

8.2.1. Quantitative data

Research staff will complete data collection in the field, and the data will be reviewed by a research assistant for completeness and accuracy on the same day. Data back-up will be performed weekly and stored in a separate building.

The study coordinator will be responsible for entering cost data into the costing spreadsheet along with relevant supporting information on: activity, cost type (capital/non-capital), useful life of item, health system level incurring the cost, major cost category (cost category 1), sub-cost category (cost category 2) and detailed cost category (cost category 3), unit type, unit quantity and unit price. All expenditure will be recorded in the currency used, most often the Indonesian Rupiah XOF converted to US Dollars ($) using mean exchange rates for each year.

8.2.2. Qualitative data

Interviews will be conducted in Indonesian and digitally recorded. Audio-recorded interviews will be transcribed by the TRF research team. Interviews will be transcribed in the local language and subsequently translated and transcribed into English. Whenever possible, the interviewers themselves will transcribe/translate audio-recordings as soon as possible after the interview occurs. Translation from local languages into English will be literal when meanings are equivalent. When, however, linguistic structures, concepts, and terms are divergent, the translation procedure will be to capture as accurately as possible the intended meaning of the respondent.

Translation quality checks will be built into the data management process, and at least one full interview will be reviewed for translation quality for each interviewer. This initial translation quality check will include all translator-transcriber research staff and involve reading the English translation while listening to the audio-recorded interview. Translation inaccuracies will be discussed and, as needed, the English translation modified. The collective results of the mid-term interview translation checks will be shared with all transcriber-translators and used to guide subsequent translation practices.

Routine transcription quality checks will involve a study investigator reading the full transcript for clarity and anonymity. When needed, researchers will return to the audio-recordings to clarify passages and modify the English translations in the transcripts when appropriate. Participant

observation notes will be typed up in the local language and translated into English, and any quotes documented in the original language. Each data file will be labelled a unique digital identifier comprising: the initials of the project, followed by the case identifier (the name/pseudonym for the case); the data collection round; the type of data (interview, diary, field notes etc.); and a unique number assigned to each file in a particular case. Data on socio-demographic and other variables derived from close-ended questions will be entered into an Excel spreadsheet along with details on the participant unique IDs and transcript file names and numbers.

8.3. DATA ANALYSIS

8.3.1. Primary and secondary outcomes

For the primary outcome of adherence and delivery effectiveness, point estimates of proportions will be provided along with the 95% CI. Between cluster variability will be assessed by calculating the ICC and coefficient of variation. Proportions for adherence and delivery effectiveness will be reported by health facility. Further exploratory analysis will be undertaken to investigate variables that are predictive of successful adherence and delivery. Statistical analysis will be done by the LSTM study coordinator with support from a study statistician using Stata (StataCorp, College Station, Tx USA) or SPSS for windows software (IBM SPSS Statistics).

8.3.2. Scalability

Transcripts will be transferred to NVivo 12 (QSR International) for data management, coding and analysis. The analysis will be performed around pre-defined themes based on the key research questions and additional themes emerging from the data using content analysis.

The analysis will draw on more than one framework whereby the initial coding will be based on the key elements of the initial logic model and additional frameworks. For health providers and managers, analysis will also draw on: 1) the health systems building blocks (finance, governance, health information, human resources, products and technology, service delivery) [27], 2) the different constructs of acceptability - affective attitude (general feelings about the intervention), burden (perceptions of effort needed to take part), ethicality (fit with their value system), intervention coherence (understanding of the intervention and how it works), opportunity costs (what must be given up in order to participate), perceived effectiveness (perceptions of the intervention's ability to achieve its aim), and self-efficacy (feeling that they are able to do what they need to do to take part) [28] and unintended consequences, and additionally 3) provider perceptions of pregnant women's preferences and adherence. For pregnant women, analysis will focus on the constructs of acceptability of the intervention and of antenatal services more broadly. Data may be coded multiple times to fit themes in different framework components and different frameworks to enable synthesized and individual analysis of the data.

We will use the Standards for Quality Improvement Reporting Excellence (SQUIRE 2, http://www.squire-statement.org/) for reporting the QI component of the study. Qualitative data on acceptability, feasibility, and appropriateness will be used to explain the quantitative findings.

8.3.3. Process evaluation

The process evaluation will utilise quantitative and qualitative data shown in table 2. Analysis will draw on both qualitative and quantitative data to explore the key implementation outcome variables developed by Peters et al[29] – acceptability, adoption, appropriateness, feasibility, fidelity, implementation cost, coverage and sustainability. Analysis of quantitative process data will begin with descriptive analysis relating to fidelity, dose/adherence, and reach. Subsequently, quantitative process measures will be integrated into outcomes datasets to understand how implementation variability affected outcomes (on-treatment analyses) and test hypotheses arising from qualitative analyses. In a final step, we will integrate qualitative and quantitative process data with primary and secondary outcomes and other implementation outcomes to clarify and explore complex causal pathways.

8.3.4. Economic studies

Decision trees capturing provider costs, adherence, uptake and health outcomes attributed to women who receive and adhere to the intervention will be constructed and used to calculate the cost per DALY averted of each intervention. Incremental cost-effectiveness ratios (ICER) will be calculated for the cohort of women receiving IPTp-DP and SST in the Timika Province. A base-case and alternative scenarios will be used to explore key structural differences in results (e.g. provider versus societal perspective, net versus gross cost-effectiveness) and probabilistic sensitivity analysis will be used to explore the impact of heterogeneity and uncertainty on outcomes. Results will be plotted on the cost-effectiveness plane with policy-relevant cost-effectiveness thresholds to be determined in partnership with MOH and applied to interpret results. Incremental cost-effectiveness ratios will be presented separately for IPTp-DP and SST, accompanied by the ICER for IPTp-DP compared with SST.

9. Ethical considerations

9.1. Human Subjects

9.1.1. Risks

Participants may find some of the questions in the interviews uncomfortable and will be informed of their right not to answer. Participants will not have to respond to questions they do not wish to and made aware that they can withdraw at any time. This will have no consequences for the care they receive. The study will include safety monitoring in study facilities which will be conducted by Timika Research Facility staff to assess for any serious adverse events. Safety data from over 10,000 women in clinical trials (5,561 women from four published trials of IPTp-DP [11-

[13,30] plus from 4,680 women in the recently completed IMPROVE trials in Africa) show no major safety concerns with the use of DP as a monthly regimen for IPTp. Similarly, no safety concerns in pregnancy have been reported in the three years since DP was introduced as first line treatment policy in Indonesia. Any adverse events will be managed according to the standard MOH referral system.

The costing analysis will require information on salary details of different health workers. However, no-one will be asked for this information. Instead, we will obtain their job title and seniority and use the mid-point salary for each grade/role from official government pay scales to estimate salary costs.

Pregnant women will not be asked about their income directly, instead, we will use an asset-based tool to establish the relative wealth/poverty and socio-economic status of the women.

9.1.2. Benefits

There will be no direct benefit for the study participants. The information gained throughout this study will provide insight on any issues regarding health provider prescribing practices for IPTp-DP to pregnant women, which will help inform gaps to be addressed by the pilot programme e.g. need for better training and dissemination of guidelines, need for more job aids or addition to the training curriculum of healthcare providers. Findings from the study could therefore help improve prescribing practices; safer prescribing practices would benefit the community and minimise the risk of exposure to malaria in pregnancy, as well as to raise general awareness of the importance of preventing malaria in pregnancy.

9.1.3. Individual consent

Written informed consent will be obtained from participants in the health facility and health manager in-depth interviews, and the exit interviews and home visits with pregnant women. This consent will be sought after written or verbal information about the purpose and procedures of the study is given in the vernacular language for each site. We will explain that participation will be voluntary and can be withdrawn at any time during the survey and that access to health care is not dependent on participation. Only participants who provide written informed consent will be enrolled. Women interviewed during home visits will be consented at the time of the interview. In the event of an illiterate participant during IDIs, an impartial witness shall attest to voluntary participation by the participant(s) so concerned. The informed consent forms will be translated to Indonesian.

Health facility cost data should be considered as public information and will not require informed consent. We will however inform health facilities in detail about the study and its purpose.

All recordings and transcriptions will only be identified by an ID number and be stored only on password-protected computers to which only project researchers have access. Throughout the data collection and analysis period, printed transcripts and any other written information will

remain in locked cabinets when not in usage. Data after the study completion will be stored in accordance of the rules of TRF and LSTM.

Anonymised health facility cost data will be obtained from the central Ministry of Health and will not require informed consent. Information on salary costs by staff grade/cadre will also be obtained from the MoH to avoid asking individuals to disclose their earnings. All other cost and resource use data obtained from participants (staff and pregnant women) will be obtained and treated in line with the informed consent process described above.

9.1.4. Consenting of emancipated minors

Young married pregnant women aged less than 18 years are considered emancipated minors in Indonesia and will sign their own consent form; parental permission will not be required for them to participate. These minors are already making their own life decisions and have the right to decline enrolment. However, an assent form will be used for unmarried pregnant women aged less than 18 years; parental permission will be required. In both situations. It is culturally accepted norm in Indonesia that an accompanying adult or husband be part of the consent and in keeping with it, they will be allowed to witness the consent. It is important to include young women in the study as adolescents are known to be particularly susceptible to malaria and are therefore one of the groups that may benefit from improvements in preventing adverse outcomes associated with malaria in pregnancy.

9.1.5. Confidentiality

No study participant will be identified by name in any report or publication derived from information collected for the study. All personal identifiers will be removed from the data when it is entered into the computer. Data collection forms will be kept a GCP compliant storage area. When reporting qualitative findings we will use respondent type e.g. health worker from sub-health centre (pustu) to give context and meaning.

9.1.6. Ethical approval

Ethical approval will be sought from LSTM’s Research Ethics Committee and the ethics committee of Universitas Gadjah Mada, Yogyakarta, Indonesia.

10. Timeline

The pilot implementation will begin in January 2022 for a duration of 16 months. The QI intervention will be introduced in Q1 2022. Data collection and processing for the evaluation will be undertaken in the last ten months of the pilot (Q4 2022-Q3 2023). Analysis and reporting will be completed by Q4 2023 to Q1 2024.

A more detailed timeline is provided in the Gannt chart below.

ACTIVITY	2021												2022												2023												2024		
Calendar Month	1	2	3	4	5	6	7	8	9	10	11	12	1	2	3	4	5	6	7	8	9	10	11	12	1	2	3	4	5	6	7	8	9	10	11	12	1	2	3
Study month	1	2	3	4	5	6	7	8	9	10	11	12	13	14	15	16	17	18	19	20	21	22	23	24	25	26	27	28	29	30	31	32	33	34	35	36	37	38	39
**Study Prep**																																							
PDRA recruitment and orientation																																							
Ethical clearance																																							
Procurement																																							
MOH training																																							
Community mobilisation																																							
Preparation of study tools, training materials and job aids																																							
Pilot Implementation duration (16 months)																																							
**QI stages**																																							
Workshop for work improvement teams																																							
Workshop to priotritise and plan QI interventions																																							
QI implementation																																							
Workshop to review impact of QI interventions (Final CQI)																																							
**Safety monitoring**																																							
**Develop health economics costing spreadsheet/model**																																							
**Health systems cost data collection**																																							
**End of project evaluation**																																							
Exit interviews (n=1440)																																							
Home visits (n=1040) - economic evaluation																																							
IDIs health providers (n=65, 32-33 per round)																																							
IDIs pregnant women (n=100, 50 per round)																																							
FGD community leaders (n=10)																																							
IDIs hubands of pregnant women (n=25)																																							
IDIs community health workers (n=10)																																							
Data entry and processing																																							
Analysis and Manuscript preparation																																							
Stock out																																							
**Dissemination**																																							
National dissemination meeting for stakeholders																																							
**Reporting and closeout**																																							

11. References

1. Dellicour S, Tatem AJ, Guerra CA, Snow RW, ter Kuile FO. Quantifying the number of pregnancies at risk of malaria in 2007: a demographic study. *PLoS Med* 2010; **7**(1): e1000221.
2. Moore KA, Fowkes FJI, Wiladphaingern J, et al. Mediation of the effect of malaria in pregnancy on stillbirth and neonatal death in an area of low transmission: observational data analysis. *BMC medicine* 2017; **15**(1): 98.
3. Moore KA, Simpson JA, Scoullar MJL, McGready R, Fowkes FJI. Quantification of the association between malaria in pregnancy and stillbirth: a systematic review and meta-analysis. *The Lancet Global health* 2017; **5**(11): e1101-e12.
4. Rijken MJ, McGready R, Boel ME, et al. Malaria in pregnancy in the Asia-Pacific region. *The Lancet Infectious diseases* 2012; **12**(1): 75-88.
5. Webster J, Ansariadi, Burdam FH, et al. Evaluation of the implementation of single screening and treatment for the control of malaria in pregnancy in Eastern Indonesia: a systems effectiveness analysis. *Malaria journal* 2018; **17**(1): 310.
6. Hill J, Landuwulang CUR, Ansariadi, et al. Evaluation of the national policy of single screening and treatment for the prevention of malaria in pregnancy in two districts in Eastern Indonesia: health provider perceptions. *Malar J* 2018; **17**(1): 309.
7. Sibley CH, Hyde JE, Sims PF, et al. Pyrimethamine-sulfadoxine resistance in Plasmodium falciparum: what next? *Trends Parasitol* 2001; **17**(12): 582-8.
8. Organization. WH. Intermittent preventive treatmentn pregnancy (IPTp). . 2018 (accessed (accessed 02 October, 2019).
9. Ahmed R, Poespoprodjo JR, Syafruddin D, et al. Efficacy and safety of intermittent preventive treatment and intermittent screening and treatment versus single screening and treatment with dihydroartemisinin-piperaquine for the control of malaria in pregnancy in Indonesia: a cluster-randomised, open-label, superiority trial. *The Lancet Infectious diseases* 2019.
10. Paintain L, Hill J, Ahmed R, et al. Cost-effectiveness of intermittent preventive treatment with dihydroartemisinin-piperaquine versus single screening and treatment for the control of malaria in pregnancy in Papua, Indonesia: a provider perspective analysis from a cluster-randomised trial. *The Lancet Global health* 2020; **8**(12): e1524-e33.
11. Kajubi R, Ochieng T, Kakuru A, et al. Monthly sulfadoxine-pyrimethamine versus dihydroartemisinin-piperaquine for intermittent preventive treatment of malaria in pregnancy: a double-blind, randomised, controlled, superiority trial. *Lancet* 2019; **393**(10179): 1428-39.
12. Kakuru A, Jagannathan P, Muhindo MK, et al. Dihydroartemisinin-Piperaquine for the Prevention of Malaria in Pregnancy. *N Engl J Med* 2016; **374**(10): 928-39.
13. Desai M, Gutman J, L'lanziva A, et al. Intermittent screening and treatment or intermittent preventive treatment with dihydroartemisinin-piperaquine versus intermittent preventive treatment with sulphadoxine-pyrimethamine for the control of malaria during pregnancy in western Kenya: an open-label, three-group, randomised controlled superiority trial. *Lancet* 2015; **http://dx.doi.org/10.1016/S0140-6736(15)00310-4**
14. Desai M, Hill J, Fernandes S, et al. Prevention of malaria in pregnancy. *The Lancet Infectious diseases* 2018; **18**(4): e119-e32.

15. Saito M, Briand V, Min AM, McGready R. Deleterious effects of malaria in pregnancy on the developing fetus: a review on prevention and treatment with antimalarial drugs. *Lancet Child Adolesc Health* 2020; **4**(10): 761-74.
16. White MT, Conteh L, Cibulskis R, Ghani AC. Costs and cost-effectiveness of malaria control interventions--a systematic review. *Malaria journal* 2011; **10**: 337.
17. Hoyt J, Landuwulang CUR, Ansariadi, et al. Intermittent screening and treatment or intermittent preventive treatment compared to current policy of single screening and treatment for the prevention of malaria in pregnancy in Eastern Indonesia: acceptability among health providers and pregnant women. *Malaria journal* 2018; **17**(1): 341.
18. Ahmed R, Levy EI, Maratina SS, et al. Performance of four HRP-2/pLDH combination rapid diagnostic tests and field microscopy as screening tests for malaria in pregnancy in Indonesia: a cross-sectional study. *Malaria journal* 2015; **14**: 420.
19. Poespoprodjo JR, Fobia W, Kenangalem E, et al. Adverse pregnancy outcomes in an area where multidrug-resistant plasmodium vivax and Plasmodium falciparum infections are endemic. *Clin Infect Dis* 2008; **46**(9): 1374-81.
20. Permala J, Tarning J, Nosten F, White NJ, Karlsson MO, Bergstrand M. Prediction of Improved Antimalarial Chemoprevention with Weekly Dosing of Dihydroartemisinin-Piperaquine. *Antimicrob Agents Chemother* 2017; **61**(5).
21. Dellicour S, Hill J, Bruce J, et al. Effectiveness of the delivery of interventions to prevent malaria in pregnancy in Kenya. *Malaria journal* 2016; **15**(1): 221.
22. Webster J, Kayentao K, Bruce J, et al. Prevention of malaria in pregnancy with intermittent preventive treatment and insecticide treated nets in mali: a quantitative health systems effectiveness analysis. *PloS one* 2013; **8**(6): e67520.
23. Hill J, Hoyt J, van Eijk AM, et al. Factors affecting the delivery, access, and use of interventions to prevent malaria in pregnancy in sub-saharan Africa: a systematic review and meta-analysis. *PLoS medicine* 2013; **10**(7): e1001488.
24. Banek K, Lalani M, Staedke SG, Chandramohan D. Adherence to artemisinin-based combination therapy for the treatment of malaria: a systematic review of the evidence. *Malaria journal* 2014; **13**: 7.
25. Moore GF, Audrey S, Barker M, et al. Process evaluation of complex interventions: Medical Research Council guidance. *BMJ* 2015; **350**: h1258.
26. Chan XHS, Win YN, Mawer LJ, Tan JY, Brugada J, White NJ. Risk of sudden unexplained death after use of dihydroartemisinin-piperaquine for malaria: a systematic review and Bayesian meta-analysis. *Lancet Infect Dis* 2018; **18**(8): 913-23.
27. World Health Organisation. Everybody's Business: Stratengthening Health Systems to Improve Health Outcomes: Framework for Action. *Geneva, WHO, ISBN 978 92 4 159607 7, http://wwwwhoint/healthsystems/strategy/everybodys_businesspdf accessed 7 June 2014* 2007.
28. Sekhon M, Cartwright M, Francis JJ. Acceptability of healthcare interventions: an overview of reviews and development of a theoretical framework. *BMC Health Serv Res* 2017; **17**(1): 88.
29. Peters DH, Adam T, Alonge O, Agyepong IA, Tran N. Implementation research: what it is and how to do it. *BMJ* 2013; **347**: f6753.
30. Ahmed R, Poespoprodjo JR, Syafruddin D, et al. Efficacy and safety of intermittent preventive treatment and intermittent screening and treatment versus single screening and treatment with dihydroartemisinin-piperaquine for the control of

malaria in pregnancy in Indonesia: a cluster-randomised, open-label, superiority trial. *The Lancet Infectious diseases* 2019; **19**(9): 973-87.

Data sharing statement

**Will individual participant data be available?**	**Yes**
**What data in particular will be shared?**	Individual participant data that underlie the results reported in this article, after de-identification
**What other documents will be available?**	The study protocol is provided in Appendix 5 and 6.
**When will data be available (start and end dates)?**	Immediately following publication and ending 36 months following article publication
**With whom?**	Researchers who provide a methodologically sound proposal
**For what types of analyses?**	To achieve aims in the approved proposal
**By what mechanism will data be made available?**	Proposals should be directed to jenny.hill@lstmed.ac.uk; to gain access, data requestors will need to sign a data access agreement

STOPMiP-2 statistical analysis plan

Statistical Analysis Plan details			
Version & date:	2.0 14 March 2023	Superseded version & date	1.0 10 May 2022

Document development details		
Author:	(Name)<br>(Signature)<br>(Date)	Jenny Hill<br>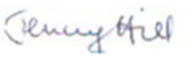<br>14 March 2023
Co-investigator:	(Name)<br>(Signature)<br>(Date)	Firdaus Hafidz<br>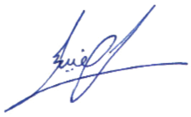<br>14 March 2023
Statistician:	(Name)<br>(Signature)<br>(Date)	Maia Lesosky<br>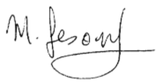<br>14 March 2023

Version History Log			
Version	Effective Date	Revision date	Reason(s) for Change
1.0	10May2022		
2.0	14Mar2023	14Mar2023	• Revised and clarified the two co-primary outcomes (adherence & delivery effectiveness) to align precisely with field procedures and paper definitions, including the special case where one facility delivers subsequent doses at home.<br>• Expanded methodological detail, including the randomisation process, and cofounding variables considered.

Related Documents	
Document code	Title

1. Introduction

This DMP relates to is an evaluation of a pilot of dihydroartemisinin-piperaquine preventive treatment (IPTp-DP) delivered by the MOH pilot programme in Papua, Indonesia.

Funding: Medical Research Council, UK.

Study type: Evaluation of a pilot implementation (Phase IV)

Primary Purpose – Prevention

1.1. Study objectives and endpoints

1.1.1. Overall aim

To assess the programme effectiveness and scalability of monthly doses of dihydroartemisinin-piperaquine preventive treatment (IPTp-DP) delivered by the MOH pilot programme to prevent malaria in pregnancy and improve birth outcomes in Papua, Indonesia.

1.1.2. Research questions

Quantitative research questions:

1. Can IPTp-DP be delivered effectively through the routine health system in Papua i.e., in a setting where antimalarials are currently only given to cases testing positive by microscopy or RDT?
2. Can pregnant women adhere to the full three-day IPTp-DP regimen, including doses to be taken at home (i.e., when given presumptively/without malaria testing)?

Economic research questions:

3. What is the incremental cost-effectiveness of implementing IPTp-DP (second and third trimesters) in addition to the current single screen and treat (SST) strategy (first trimester) in the routine health system?
4. What is the incremental financial cost of implementing IPTp-DP (provider perspective) at scale in Indonesia?

Qualitative research questions:

5. Do health providers accept IPTp-DP and what do they perceive to be the drivers of successful integration and scalability to inform potential policy rollout?
6. Do pregnant women accept IPTp-DP and how could delivery be improved?
7. Can embedded continuous quality improvement (CQI) be used to improve IPTp-DP delivery and uptake? (using routine CQI reports and HMIS data/dashboard)

1.2. Outcomes and definitions

Co-Primary outcome: Delivery effectiveness

Original definition: The proportion of women attending ANC treated appropriately according to the IPTp-DP guidelines, defined as the first dose given by DOT plus adequate doses to take home for days 1 and 2. Their understanding of the treatment regimens given during that ANC visit will also be assessed

Revised definition: The proportion of women attending ANC treated appropriately according to the implementation of IPTp-DP guidelines at facility level as assessed at the time of assessment [any method, any course of IPTp, with or without other treatment].

The standard DP treatment regimen will be used, three tablets per day for three days i.e., a total of nine tablets in total. An appropriate dose of IPTp DP* is defined as the correct dosage of IPTp reported on leaving the facility with the correct number of tablets for subsequent doses (6 tablets with DOT, 9 without DOT). The number of tablets received by each woman is corroborated by pill count at ANC exit.

**Post hoc amendment: At one facility subsequent doses were not dispensed at ANC, rather ANC midwives delivered the tablets for subsequent doses (days 2 and 3) to women at home.*

Delivery effectiveness was categorised into 3 main categories - full, partial and non-effective delivery. Post-hoc amendments were made to include additional sub-categories for each main category based on variations which emerged during implementation, as follows:

1. Full delivery effectiveness (Condition 1)
   - Three tablets administered under directly observed therapy (DOT) for the first dose at the facility, with the remaining six tablets dispensed for self-administration at home (days 2 and 3)
   - Three tablets administered under DOT for the first dose, with the remaining six tablets delivered to the woman's home (e.g. by midwife or outreach worker) for supervised or supported administration.
2. Partial delivery effectiveness (Condition 2)
   - The entire nine-tablet course (three per day for three days) is dispensed at once for self-administration, with no DOT for the first dose.
3. Non-effective delivery (Other conditions)
   - Any dispensing pattern that does not meet the criteria for full or partial effectiveness—for example, fewer than nine tablets issued—indicating inadequate delivery.

Co-Primary Outcome: Adherence

Original definition: The proportion of pregnant women who receive the first dose of IPTp-DP by directly observed therapy (DOT) at ANC and have the correct number of DP tablets for subsequent doses on exit, and when visited at home, have verified that they completed the treatment (adherence).

Revised definition: The proportion of pregnant women who receive correct dosage of IPTp-DP (6 with DOT, 9 without DOT) on exit*, with or without other treatment, and verify they have completed treatment (home visit).

**Post hoc amendment: 1 facility did not give the doses for days 2 and 3 at the facility, rather the midwives delivered the tablets to women in their homes.*

Adherence in this context is related to the extent to which pregnant women follow the prescribed regimen of dihydroartemisinin-piperaquine (DP) as part of a preventive treatment against malaria. It is measured against the following conditions:

1. Full Adherence (Condition 1):
   - The woman completed the entire course as prescribed: six tablets of subsequent doses (if taken with DOT), or nine tablets (if taken without DOT).
2. Partial Adherence (Condition 2):
   - The woman took some but not all of the prescribed tablets, ie. Fewer than six tablets (if taken with DOT), or fewer than nine-tablet doses (if taken without DOT), indicating partial compliance.
3. Not Adhere (Other conditions):
   - The woman did not take any tablets administered by health facility, or did not meet the criteria for full or partial adherence.

2. Study methods

2.1. General study design and plan

Evaluation of a pilot implementation of IPTp-DP in 10 selected health facilities in Papua, Indonesia. The health facilities were selected from 21 health facilities in Papua based on accessibility and functionality to be the pilot study sites. The remaining health facilities have been excluded due to accessibility issues.

2.2. Study population

Inclusion-exclusion criteria of pregnant women (health facility exit interviews, home visits)

Inclusion criteria:

- Pregnant women aged 15-49 years
- Women in 2nd/3rd trimester of pregnancy

- HIV negative (where status is known)

Exclusion criteria:

- Women with communication or language problems
- Pregnant women are unwell during the interview
- Pregnant women who move outside the pilot implementation areas

2.3. Randomisation and blinding

Each morning, the data collectors estimate the day's ANC attendance and prepare a corresponding number of lottery tickets. They then randomly draw tickets to determine which women will be invited to participate. Each drawn number is recorded and handed to the midwife, who cross references the queue numbers at registration. The midwife then informs the corresponding women that they have been selected for the exit interview. Those women are then directed by the midwife to the data collector, who introduces the study, confirms eligibility, and obtains informed consent before proceeding with the exit interview.

2.4. Visit schedule and measurements

Delivery effectiveness will be evaluated using an endline cross-sectional survey of delivery effectiveness of IPTp-DP at ANC assessed through exit interviews with pregnant women, and longitudinal follow-up of adherence to DP assessed through home visits. A health facility audit, together with unstructured observations, will be used to provide context to the findings of the exit interviews.

Women who receive the correct dose of IPTp-DP as assessed in the exit interviews will be followed up at home on day 4 or 5 after their clinic visit (i.e., one to two days after the third dose of 3-day DP) and interviewed about adherence (self-report), and pill counts performed.

3. Sample size

Estimated number of participants

Delivery effectiveness (exit interviews): 1,440 pregnant women

We expect to enrol a total of 1,440 pregnant women for the co-primary outcome of systems effectiveness, measured via exit interviews. Allowing for an expected systems effectiveness of 75%, there will be an estimated n = 1,080 women available for analysis of the co-primary endpoint of adherence rate, measured at home visits.

Adherence (home visits): 1,080 pregnant women

A sample size of 1,080 pregnant women from ten clinics (an average of 108 per clinic) will allow the detection of an estimated 60% of women achieving the primary endpoint

with a precision of +/-6%, 95% CI, and a design effect (DE) of 4.22 based on an Intra-Cluster Correlation Coefficient (ICC) of 0.03.

4. Statistical methods

4.1 General considerations

All tests will be two-sided and unless otherwise defined p-values < 0.05 will be used to define statistical significance for analysis. Adjustment for multiple testing will not be applied unless otherwise specified. Where distributional assumptions are necessary for correct evaluation of statistical results, appropriate descriptive, diagnostic evaluation and (if required) transformation of variables will be carried out. In the event the parametric tests indicated are unable to be used, non-parametric versions will be used. All estimates will be provided with measures of variance or confidence intervals, as appropriate.

4.2 Timing of analyses

The final analyses will occur as soon as possible after the completion of last follow-up and after data cleaning and database closure, and after the finalisation and approval of this SAP document.

4.3 Analysis populations

Intention to treat population
This will include all women completing an exit interview (co-primary outcome 1) and all women completing a home visit (co-primary outcome 2)

Safety population
Not applicable for this study.

4.4 Covariates and subgroups

Subgroups evaluated will be:

- Health facility
- Receive SST or not

4.5 Missing data

Primary data analysis will be carried out without imputation of key endpoint variables. Demographic covariates may be imputed to retain all individuals in the analysis.

4.6 Interim analysis and data monitoring

Not applicable for this study.

4.7 Summary of study data

Continuous data will be summarised using medians and interquartile range, sometimes with mean and range values. Categorical data will be summarised with frequency, and percentages (based on non-missing sample size) using all observed levels. In general, data will be listed, sorted by treatment and visit number where appropriate. Summary tables will be structured with a column for each treatment and will be annotated with the total population size relevant to that table/treatment, including any missing observations. Analysis populations will be as described in Section 4.3.

4.8 Endpoint analyses

Co-Primary endpoint (systems effectiveness): Proportion (95% confidence interval) of women attending ANC meeting the site level treatment definition (section 1.2)

Co-primary endpoint (adherence): Proportion and 95% confidence interval for the proportion of women adhering to treatment regimen.

Secondary analyses:

Regression analysis to estimate factors associated with co-primary endpoint definitions. Univariable (single explanatory covariate) and multivariable models will be fit using a binomial model with logit link function to estimate odds ratios (95% confidence intervals). Covariates will be selected using [directed acyclic graphs] and prior knowledge regarding explanatory and/or confounding factors.

Explanatory variables considered for adjusted regression analysis

Delivery effectiveness (cluster level):

- Health facility characteristics e.g., client: staff ratio, stockouts
- Health provider characteristics e.g., cadre, access to training or job aids
- Pregnant women characteristics e.g., trimester, parity, age

Adherence (individual level):

- Pregnant women characteristics
- Household characteristics e.g., SES

Confounding variables considered for adjusted regression analysis:

Table 1 Table of potential predictors of delivery effectiveness

**Predictors**	**Data source***
Pregnant women and household factors	
Age	1
Education	1

Statistical analysis plan

Predictors	Data source*
Socio-economic status	1
Ethnicity	1
Marital status	1
Religion	1
Gestational age (trimester)	1,2
Gravidity	1,2
ANC visit related factors	
ANC visit no	1,2
Has an illness	1
Previous malaria test within the past 28 days	1,2
Previous IPTp	1,2
Health insurance ownership	1
Side effects to medicines given today	1
Health facility factors	
Location of health facility	3

*1- interview (recorded or self-report); 2-ANC book; 3-health facility audit

Table 2 Table of potential predictors of adherence

Predictors	Data source*
Pregnant women and household factors	
Age	1
Education	1
Socio-economic status	1
Ethic group	1
Marital status	1
Religion	1
Gestational age	1,2
Gravidity	1,2
Acceptability	1
Side effect (from a previous dose)	1
ANC visit related factors	
ANC visit no	1,2
First ANC	1,2
Illness reason for visit	1
Previous malaria test within the past 28 days	1,2
Previous IPTp	1,2
Health insurance ownership	1
Health facility factors	
Location of health facility	3
Full or partial effective delivery	1

*1- interview (recorded or self-report); 2-ANC book; 3-health facility audit
